# Integrative transcriptome analysis suggest processing of a subset of long non-coding RNAs to small RNAs

**DOI:** 10.1186/1745-6150-7-25

**Published:** 2012-08-07

**Authors:** Saakshi Jalali, Gopal Gunanathan Jayaraj, Vinod Scaria

**Affiliations:** 1 GN Ramachandran Knowledge Center for Genome Informatics, CSIR Institute of Genomics and Integrative Biology (CSIR-IGIB), Mall Road, Delhi, 110007, India; 2 Proteomics and Structural Biology Unit, CSIR Institute of Genomics and Integrative Biology (CSIR-IGIB), Mall Road, Delhi, 110007, India

## Abstract

**Background:**

The availability of sequencing technology has enabled understanding of transcriptomes through genome-wide approaches including RNA-sequencing. Contrary to the previous assumption that large tracts of the eukaryotic genomes are not transcriptionally active, recent evidence from transcriptome sequencing approaches have revealed pervasive transcription in many genomes of higher eukaryotes. Many of these loci encode transcripts that have no obvious protein-coding potential and are designated as non-coding RNA (ncRNA). Non-coding RNAs are classified empirically as small and long non-coding RNAs based on the size of the functional RNAs. Each of these classes is further classified into functional subclasses. Although microRNAs (miRNA), one of the major subclass of ncRNAs, have been extensively studied for their roles in regulation of gene expression and involvement in a large number of patho-physiological processes, the functions of a large proportion of long non-coding RNAs (lncRNA) still remains elusive. We hypothesized that some lncRNAs could potentially be processed to small RNA and thus could have a dual regulatory output.

**Results:**

Integration of large-scale independent experimental datasets in public domain revealed that certain well studied lncRNAs harbor small RNA clusters. Expression analysis of the small RNA clusters in different tissue and cell types reveal that they are differentially regulated suggesting a regulated biogenesis mechanism.

**Conclusions:**

Our analysis suggests existence of a potentially novel pathway for lncRNA processing into small RNAs. Expression analysis, further suggests that this pathway is regulated. We argue that this evidence supports our hypothesis, though limitations of the datasets and analysis cannot completely rule out alternate possibilities. Further in-depth experimental verification of the observation could potentially reveal a novel pathway for biogenesis.

**Reviewers:**

This article was reviewed by Dr Rory Johnson (nominated by Fyodor Kondrashov), Dr Raya Khanin (nominated by Dr Yuriy Gusev) and Prof Neil Smalheiser. For full reviews, please go to the Reviewer’s comment section.

## Background

The availability of high-throughput technology including next-generation sequencing to understand the structure and function of the genome offers a new window to understand genome function through precise and high-resolution mapping of transcriptional landscape of the genome. Many recent studies have revealed the presence of a large number of non- protein coding functional transcripts encoded by genomes of higher eukaryotes [1-3]. Many of these functional non-coding transcripts are encoded by regions in the genome that was previously not known to transcribe for protein-coding genes. Apart from the well studied classes of non-coding RNAs like microRNAs (miRNAs) [4], long non-coding RNAs (lncRNAs) form a major class of ncRNAs. By definition, lncRNAs are transcripts which are more than 200 bases in length and does not code for a putative functional protein [5]. The classification presently also encompasses a previously known class of transcribed pseudogenes and antisense transcripts apart from the newly discovered class of large intergenic non-coding RNAs (lincRNAs) [3]. The amenability of technology for deep sequencing of transcriptome [6] and computational tools to understand transcript diversity, structure and expression has led to the discovery of lncRNAs in many organisms spanning the eukaryotic genomes [7,8]. lncRNAs have recently received immense attention, considering their implication in critical biological regulatory functions in cell cycle and involvement in pathological phenomena like neoplasia [9,10]. The present understanding of the molecular mechanisms and functional roles of lncRNAs is limited and based on the studies of a very few lncRNAs. Latest reviews have proposed that emerging molecular and computational biology techniques can act as catalyst in discovering lncRNA-mediated regulation via its interaction with different biomolecules leading to prediction of potential therapeutic targets [11]. Recent catalogs of lncRNAs in humans reveal a wide diversity of functional biological processes in which these RNAs participate [2,3]. Though molecular mechanisms and the regulatory roles of many of the lncRNAs are still unknown, there is one major molecular mechanism known to be involved in recruitment of chromatin modifiers [12]. It has been reported that many of the lncRNAs take part in gene regulation and epigenetic processes [10,12,13]. A few mechanisms also include formation of heterochromatin, telomere maintenance, interaction with other classes of ncRNA including miRNAs [14] which are known to modulate gene expression [15,16]. Our group has recently [17] suggested that lncRNA could operate to titrate proteins via presence of G-quadruplexes along their length that potentially lends a structural scaffold.

Dual regulatory outputs of transcripts are not very well studied in the field of genomics. One of the earliest evidence of dual functional transcripts in biology is from the prokaryote *Escherichia coli*, where a transcript specifies both information, as well as structural functions [18]. Another well studied example for RNA with dual functional output is the SRA1 which codes for a well conserved protein as well as RNA based co-regulator [19]. Recent reports have suggested the role of small nucleolar RNAs (snoRNAs) in having dual functional output by virtue of being processed to smaller RNAs [20,21]. miRNAs originating from the introns of protein-coding genes, otherwise called ‘mirtrons’ have also been shown to be an example of dual transcriptional output [22,23].

Building upon these previous reports, we hypothesized that a subset of the lncRNAs could potentially provide for dual functional or regulatory outputs by acting as host RNA and giving rise to small RNA species. Unlike many other classes of RNAs like miRNAs, which are extremely conserved, earlier computational analyses have suggested that many of the lncRNAs are not very conserved across vertebrates, and conservation at the most is restricted to patches along the ncRNAs suggesting discrete functional domains or regions in the lncRNAs [5,24]. The availability of a number of experimental small RNA deep sequencing datasets in public domain [25,26] motivated us to address the hypothesis that many of the lncRNAs have potential to encode for smaller functional RNAs. We performed our analysis and further tested the hypothesis on two independent lncRNA annotation datasets. One dataset is compiled by integration of manually curated lncRNA annotations while the other is a publically available genome-wide transcriptome database. Our analysis suggests that many of the well annotated lncRNAs are potentially processed to small RNAs. Further analysis indicated that the steady state levels of these small RNAs vary in different tissues or cell types, suggesting a coordinated and regulated process of biogenesis. We propose this could be a novel mechanism of integrating regulatory signals. To the best of our knowledge, this is the first report suggesting a potential dual regulatory output in a subset of lncRNAs. We hope further experimental analyses would reveal enormous mechanistic insights into the function and regulation of lncRNAs.

## Results

### Significant number of small RNA clusters map back to lncRNA

The lncRNAs downloaded from lncRNAdb [27] had an average length of approximately 2 kb with Xist being the largest lncRNA having a sequence length of 59 kb. The distribution of the lncRNAs lengths is summarized as supplementary information (Additional File 1). The small RNA clusters of lengths ranging from 47 nucleotides to around 7 kb were retrieved from deepBase [26] database. The complete list of mappings of small RNA clusters onto lncRNAs with the relative positions of lncRNAs and the small RNA clusters is summarized in Additional File 2. Comprehensive analysis of small RNAs revealed 30 lncRNAs harboring 69 small RNA clusters as derived from clustering of small RNA sequencing reads.

In addition, we also performed similar analysis on an independent dataset of lncRNAs recently made available through Gencode [26]. The dataset had a total of 58857 exons and 41310 introns. We mapped the deepBase small RNA clusters onto the lncRNAs exonic and intronic regions. Analysis revealed 1575 small RNA deepBase clusters mapping onto exons with a length adjusted frequency of 0.093 per kilobase (1575 clusters) while clusters mapped with a frequency of 0.042 per kilobase to the introns (20959 clusters). A similar analysis of protein-coding genes revealed a length adjusted frequency of 0.29 per kilobase for exons (52295 clusters) and 0.059 per kilobase for introns (273771 clusters) (Additional File 3). Thus, our observation reveals that there is a preference of the smallRNAs to map to the exons of lncRNAs.

### Novel, uncharacterized small RNA species originate from lncRNA

We examined the repertoire of small RNA species which potentially arise from these lncRNA regions. Analysis revealed that for the 69 small RNA clusters mapping onto lncRNAs from lncRNAdb, one of the cluster could be annotated as miRNA (hsa-mir-675) when mapped to miRBase [28] and 9 small RNA clusters were catalogued as 41 promoter associated RNAs (pasRNA) (from deepBase). Evaluation of an independent Gencode dataset revealed 12 miRNAs, 695 non-coding associated RNAs (nasRNA) and 1052 pasRNA out of the 1084 small RNA clusters (see Additional File 4) mapping onto Gencode lncRNAs. Thus, suggesting that apart from the known annotated miRNAs, nasRNAs and pasRNAs which comprises of only a small proportion of small RNAs, potentially many other novel classes of small RNAs can be derived from the lncRNA loci. At this stage, it would be too early to discuss the implications of this finding as newer miRNAs and other novel smallRNA classes are still being discovered in multiple tissue types [29,30].

### Large number of small RNA clusters originates from the ends of the lncRNA

We also examined the positional preference of the small RNA clusters across the lncRNA length. The lncRNA transcripts were arbitrarily divided into three equal parts across their length; termed as 5’ end, the 3’ end and the middle segment. Comparative analysis revealed a positional preference of the small RNA clusters mapping with respect to the 3’ end of the lncRNAs (Additional File 5). Similar preference for small RNA clusters at the ends of the lncRNAs was also observed for Gencode dataset.

### Well known lncRNA also seem to harbor small RNA

**Figure 1:**
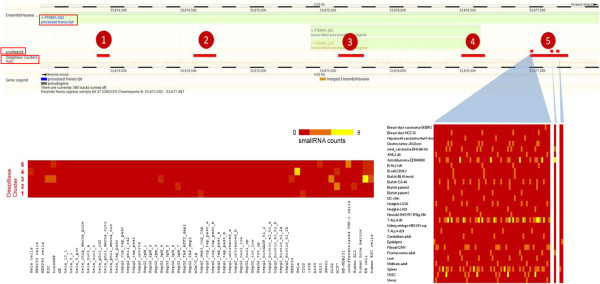
**An example depicting the PTENP1 lncRNA harbouring five small RNA clusters (numbered 1–5) derived from deepBase.** The fifth cluster is shown to have differential expression of the 3 smallRNAs which are mapped on it using the smallRNA cloning data from smiRNAdb database. The subset of the figure also shows the correlation of differential expression of the clusters in different tissues and cell-lines.

Detailed analysis revealed that the small RNA clusters indeed map to many well studied and annotated classes of lncRNAs, such as MALAT1, XIST, TUG1 and PTENP1. PTENP1 is a pseudogene of the PTEN gene, which is a well studied tumor suppressor gene. PTEN is known to regulate Phosphatidylinositol Kinase/AKT pathway [31] and have been shown to be deleted or down regulated in tumors [32]. PTENP1, the pseudogene of PTEN has been shown to biologically regulate the expression of PTEN [16]. Our analysis reveals that PTENP1 harbors five clusters of small RNAs as annotated by deepBase. This observation was also corroborated by an independent dataset of small RNA cloning data from smiRNAdb [33] which revealed that the fifth cluster comprises of three distinct small RNA clusters, having differential expression levels in different tissues (Figure 1). This could lead to a possibility whereby apart from the PTENP1 function, the processed small RNAs could be an additional mechanism for modulating biological processes in the cell and potentially in the pathogenesis of oncogenesis. Although, how this could happen remains an open ended question which we have briefly discussed. 

Similarly Malat1 (Metastasis associated lung adenocarcinoma transcript 1) is a well studied lncRNA now known to be involved in the pathogenesis of cell invasion and metastasis [34,35]. Malat1 has been shown to be up-regulated in a variety of malignancies, mostly invasive malignancies [36]. Malat1, though expressed in many tissues, is highly expressed in neurons where it is thought to regulate neuronal function by influencing synapse formation and density of synapses [37]. Malat1 has also been previously shown to be processed into a smaller transcript of 61 bases in length [38]. This processing has been shown to be mediated through Ribonuclease P (RNAseP/RNaseP) that acts on a smaller transcript at the 3’ end of nascent Malat1 transcript. This processed transcript is further taken up by the enzymes involved in tRNA processing machinery to produce RNA analogous to tRNA’s structure. Our analysis suggests that there may be additional sites of RNA processing in Malat1 lncRNA. A cluster of small RNA of approximately 7000 nucleotides extending between positions 65266515 and 65273645 on chromosome 11, encodes for a large number of small RNAs which are differentially expressed in tissues or cell types. Moreover, it is seen cumulatively, that most number of small RNA clusters from all independent datasets map back to Malat1 significantly (Additional File 2). However, it is difficult to ascribe a specific functional role to this observation without further experimental examination of Malat1 functions in the given context. Extending our analysis to the entire dataset, we found similar patterns in other functionally well annotated lncRNAs like NEAT1, TUG1 (Additional File 6) and Xist suggesting this could be a potentially generalized mechanism operating in lncRNAs. A recent overview of lncRNA function summarized the potential role of lncRNAs as (a) signals (b) decoys (c) guides and (d) scaffolds [39].

## Discussion and conclusion

We argue that a subset of lncRNAs could also potentially function by producing a dual transcriptional output through an additional layer of regulated processing. It is likely that these small RNAs could get incorporated in other regulatory mechanisms and further regulates other transcripts in *trans* such as well known classes of small RNAs like miRNAs. However, it is difficult to ascribe how these small RNA processing events take place. Here, we have considered the following scenarios: 1) these small RNAs could arise from a *cis* phenomenon like self splicing 2) these small RNAs could be products of non-specific lncRNA degradation and 3) certain endoribonucleases could site specifically process them. First, we considered if these were *cis* phenomena analogous to self splicing, however, this seems most unlikely to be the primary mechanism producing small RNA since we scored for clusters mapping back to lncRNA exons alone. We then examined how non-specific degradation could be coordinated across different cell types and tissues, which seemed unlikely. We also noted that the non-specific degradation could not possibly give rise to a positional preference to any region of the lncRNA studied here. We therefore, hypothesized that certain ribonucleases like the aforementioned example could specifically process these lncRNA by certain recognition cues [38]. We could not comment on this conclusively due to unavailability of large genome-wide datasets in public domain on RNA binding proteins and their interactions. We expect that availability of transcriptome-wide assays for RNA binding proteins (which have ribonuclease activity) like high-throughput sequencing of RNA isolated by cross-linking immunoprecipitation (HITS-CLIP) [40] or Photoactivatable Ribonucleoside Enhanced Crosslinking and Immunoprecipitation (PAR-CLIP) [41] would allow one to conclusively address this issue.

**Figure 2:**
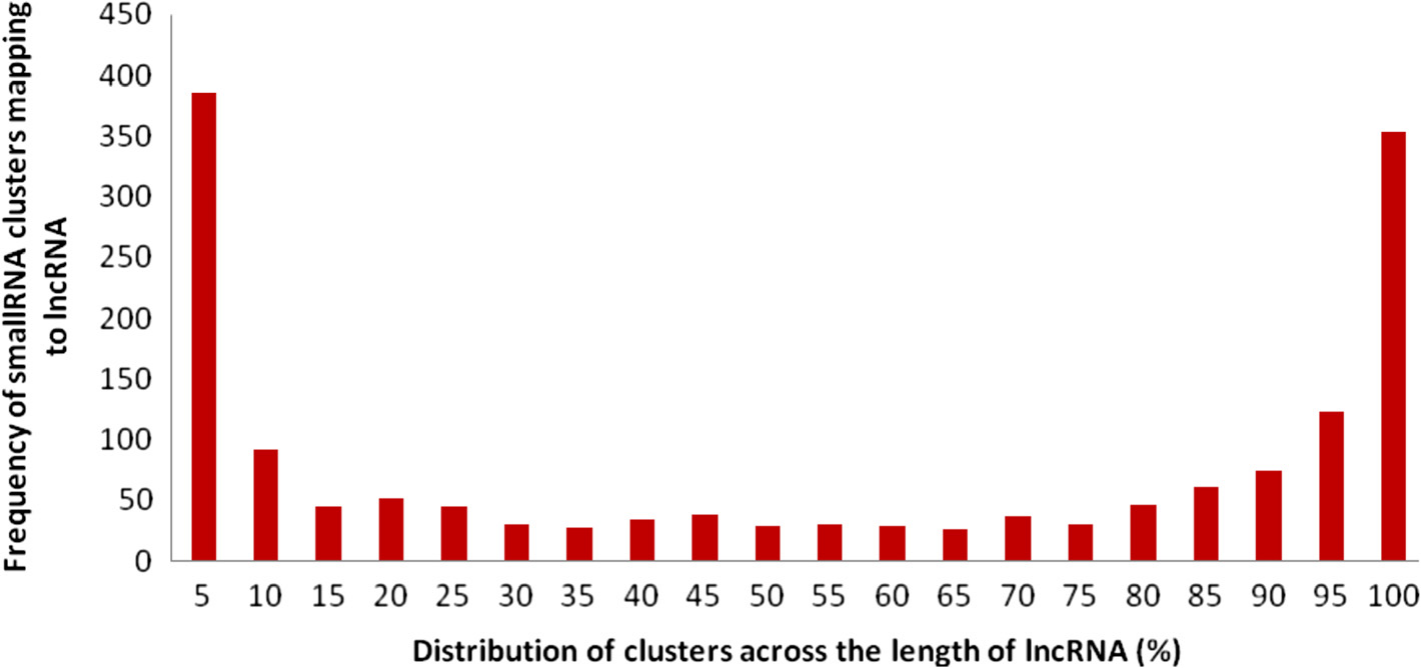
Representation of the entire length of lncRNAs derived from Gencode which was divided into frames of 5 percent each and are plotted against the mapping frequencies of small RNA clusters exhibiting positional preference of the clusters towards ends of lncRNAs.

We also observed a positional preference for small RNA clusters in the lncRNA loci with significant number of the small RNAs originating from the ends of lncRNAs (Figure 2). This potentially suggests a modular mechanism for coordinating the processing of small RNAs at these loci. We have compared and mapped known miRNA loci in lncRNAs. Several other cases of miRNAs now being annotated at lncRNA loci have been recently reported [35] suggesting this could be a more frequent phenomenon than previously known. 

There are many caveats to this analysis, the foremost being that the paucity of genome-scale datasets precludes us from suggesting a potential biological machinery and mechanism by which small RNA processing could happen. Secondly, though our evidence suggests that the small RNAs could be processed in a tissue or cell-type specific fashion, the biological output of these small RNAs and their molecular mechanism of action are largely unknown. The third major caveat is the lack of annotation on lncRNAs in public domain databases. Although thought to be pervasively transcribed, and suggested to be a major class of non-coding functional RNA, there has been paucity of well annotated and curated datasets of lncRNAs which limits the generalization of analysis. Furthermore the paucity of datasets and limitations in computational methods to ascribe functions for lncRNAs and the smallRNAs limits our study to providing circumstantial evidence supporting the hypothesis rather than proving it beyond doubt. We hope this report would also provide a much needed starting dataset for experimental biologists for validating and elucidating potential molecular mechanisms. It has also not escaped our attention that existence of such a mechanism could provide novel insights into elucidating functional variations in the genome at lncRNA loci.

## Methods

### Datasets

The initial lncRNA datasets were derived from the publicly available lncRNAdb database [27]. The database provides sequences and annotations of well studied and experimentally validated lncRNAs in human and mouse. The sequences were downloaded and mapped to hg19 build of the human genome using BLAT [42]. The online BLAT interface available at the UCSC Genome Browser [43] was used with default settings. All mappings which covered more than 90 percent span of the input sequence were compiled. The alignment blocks (henceforth described as lncRNA exons) were further manually verified to annotate the exons. This final mapped dataset encompassed a total of 72 lncRNAs encompassing 341 alignment blocks.

The dataset of small RNAs were derived from deepBase [26], which integrates a number of small RNA experiments and uses an elaborate classification schema to classify small RNA loci. The dataset organizes the small RNA loci as clusters. The small RNA clusters and their respective annotations were downloaded from the website and the dataset comprised of 408,009 small RNA clusters. deepBase also quantifies the reads mapping to each of the clusters and the tissue/cell type libraries from which the data was derived, thus serving as a ready resource to understand tissue-specific differential expression at each of the small RNA cluster loci. In addition, we also downloaded an independent dataset of small RNA cloning data from smiRNAdb [33]. The dataset consisted of 60,355 loci derived from 170 tissues. Further, we obtained 4 small RNA datasets from ENCODE project which contained small RNA cluster tags for 2 cell lines (Gm12878 and K562).

We also performed our additional analysis on an independent dataset of lncRNAs, recently annotated as a part of Gencode [44]. The data was derived from Gencode Version 10 (November 2011 freeze, GRCh37) (http://www.gencodegenes.org/) a publicly available database. The dataset included a total of 28,389 long non-coding transcripts comprising of 58,857 exons and 41,310 introns with annotations from Ensembl (Havana). The small RNA dataset derived from deepBase were mapped onto the lncRNA exonic positions and intronic positions using custom scripts. Similarly mappings were also carried out on the Gencode protein coding exons (n = 761937) and introns (n = 683105).

### Mapping of small RNA clusters

**Figure 3:**
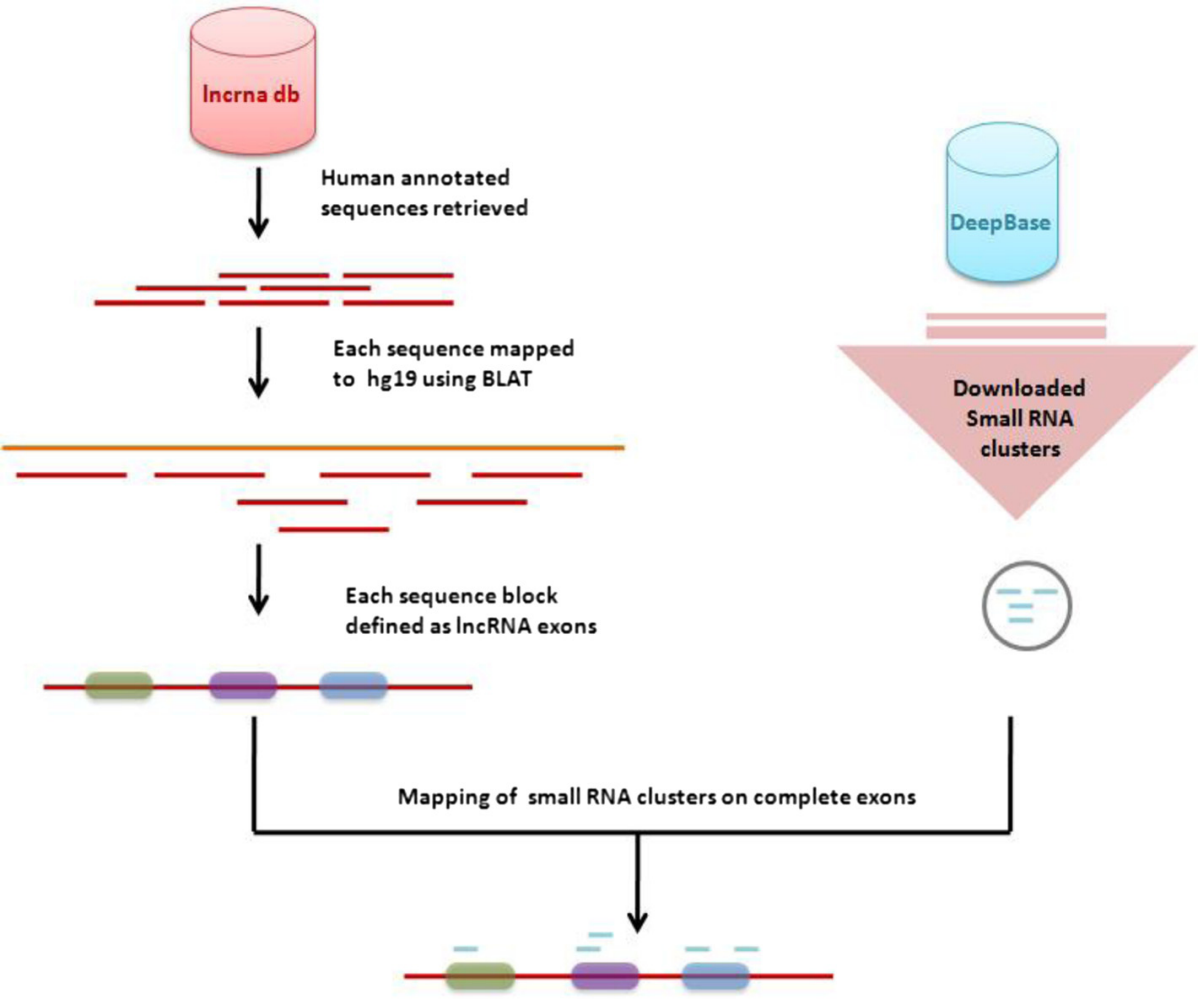
An overview of data mapping and integration for analysis of small RNA clusters in lncRNA loci.

The mapping of small RNAs to lncRNA exons from lncRNAdb was performed using bespoke script written in Perl. Only small RNAs mapping entirely to an lncRNA exons and mapping in the same strand orientation were considered for further analysis. The expression data was retrieved for respective mapped clusters manually for comparison at tissue level. We additionally performed mappings using a similar strategy across the lncRNA exons and introns on the independent dataset of human lncRNA annotations from Gencode. An overview of the computational analysis pipeline is described in Figure 3.

## Reviewers' comments

### Reviewer’s Report

**Title:** Integrative transcriptomes analysis suggest processing of a subset of long non-codingRNAs to small RNAs

**Versions:** 1, 2 & 3 9 January 2012 /12 April 2012/21 June 2012.

**Reviewer Number:** 1.

**Reviewer:** Dr Rory Johnson (nominated by Fyodor Kondrashov).

1. Sample size: The authors carry out their analysis on an extremely limited set 72 of manually curated RNAs from lncrnadb. Therefore, results from such a small set offer us very little insight into whether this is a general phenomenon or not. Large lncRNA annotations with several thousand examples in mouse or human have been available for quite some time (eg Jia et al. PMID 20587619, He et al. PMID 18000000). I am confused why the analysis was not carried out using such a set.

***Author's response:**** Our initial analysis was initially carried out on a smaller dataset of 72 manually curated lncRNAs derived from lncRNAd, a publicly available database. This database contains examples of lncRNAs which have some level of biological function assigned to them based on literature evidence. This analysis revealed that 30 lncRNAs harbor 69 small RNA clusters as derived from clustering of small RNA sequencing reads; results as depicted in Additional File**2**. We have now extended the analysis using a larger dataset of manually curated 28,389 lncRNAs transcripts from Gencode version 10 (November 2011, GRCh37) (http://www.gencodegenes.org/). We followed the similar methodology as depicted in Figure*3* to map the deepBase clusters (of size not more than 100 bp) onto the lncRNAs exonic regions. This additional analysis revealed a total of 1575 mappings where 1259 lncRNAs exons harbor 1084 small RNA clusters (Additional File*3*).*

2. Data presentation: The authors principle analysis is to compare the genomic coordinates of (a) lncRNAs and (b) small RNA clusters. This is not a particularly challenging analysis, although nevertheless important. However, the authors do not make the results available in any useful format. Table 1 shows the genomic coordinates of the lncRNAs in question, but no useful information about the overlapping deepBase clusters. How are future researchers supposed to validate or use this data without the very most basic location information for the overlapping clusters?

***Author's response:**** We thank the reviewer for this suggestion. As suggested by the reviewer Table 1; now Additional File**2** (i.e. the tabular summary of lncRNAs and small RNA clusters mappings to lncRNA exons derived from lncRNAdb.) has been modified with additional information to provide researchers with a ready set of reference to potentially prioritize them for further experiments. We have included two additional columns in Additional File**2** i.e. “Cluster locations” and “Strand” in addition to the existing columns “lncRNA Name”, “Genomic Position”, “Length of lncRNA” and “deepBase Clusters”.*

3. Data validation: Another major problem with this analysis is that the authors make absolutely no effort to understand whether the overlaps that they observe between lncRNAs and small RNA clusters is any different from what you expect by chance. There are many analyses that come to mind to see whether the observed overlap is particularly high or not. It is important to calculate the actual overlap rate, and various negative and positive control overlap rates, and calculate a resulting P value for the differences. The authors do not even mention whether the small RNA clusters originate on the same or opposite strand of the supposed host lncRNA transcript – this is crucial, I cannot find it mentioned in the Materials and Methods. Other important questions: do the small RNAs originate from introns or exons of lncRNAs? Do small RNA clusters overlap protein coding genes at the same rate as lncRNAs? Another useful control analysis would be to specifically select lncRNAs that are known to be small RNA precursors (ie microRNA precursors, or snoRNA precursors) and calculate the rate of overlap here, as a comparison.

***Author's response:**** We thank the reviewers for the suggestion, though we do not subscribe to the assumption that the smallRNAs mapping to lncRNAs should be significantly enriched to assume processing or biological functionality. We have indeed compared the mapping frequencies to exons and introns in protein-coding as well as long non-coding genes. Analysis revealed 1575 small RNA deepBase cluster mapped onto lncRNA exons with a length adjusted frequency of 0.093 per kilobase (1575 clusters) while clusters mapped with a frequency of 0.042 per kilobase to the introns (20959 clusters). A similar analysis of protein-coding genes revealed a length adjusted frequency of 0.29 per kilobase for exons (52295 clusters) and 0.059 per kilobase for introns (273771).*

*We thank the reviewer for pointing out that the Materials section did not contain adequate information on the strand/orientation of the transcript and smallRNA clusters. The small RNA clusters which overlap host lncRNA were mapped keeping into consideration the strand as depicted in Additional File**2** and Additional File**3**. The Materials and Methods section of the manuscript also has been modified accordingly*.

As far as I can see the data in Additional File 2 has at least two important errors, which lead me to doubt the quality of this dataset:

1) BC200 is a human repeat element, with many copies throughout the genome. So how do you specifically overlap this sequence with small RNA clusters at one discrete genomic locus?

***Author's response:**** We agree to the reviewers comment, that BC200 is a human repeat element composed of 200 nucleotides and with huge number of copies present across the genome. In the present analysis, we used the computational method as described in Figure*3*, which involved mapping the lncRNA sequences from lncRNAdb to the Human genome using BLAT (as the genomic positions were not available for the respective lncRNAs in lncRNAdb). The BLAT result showed 204 hits for BC200, but we only considered the hits with exact matches or rather the best match. There was only one entry with exact match for BC200 wherein it mapped to chr2 on positive strand of hg19. In fact this genomic position of the lncRNA is also corroborated by independent annotations for transcript with ID: ENSG00000236824.*

In response to my previous question, the Authors stated that “The small RNA clusters which overlap host lncRNA are originating from the same strand as depicted in Additional File 2 and Additional File 3”. A quick inspection shows that at least in 3 cases (H19, ST7OT1, ZFAS1), the RNA cluster strand information provided in Additional File 2 is on the OPPOSITE strand as the indicated lncRNA. For example, H19 is on the – strand, while the sRNA cluster is indicated to be on the + strand. So, did the authors filter their lncRNA / small RNA cluster overlap in such a way to ONLY include cases where both are on the same strand?

***Author's response:**** This has been corrected in revised manuscript. The small RNAs clusters which exactly fall within the lncRNAs on the same strand were only considered in our analysis.*

4. Downstream analysis: The authors do some expression analysis of their discovered small RNA clusters, but frankly Figure 3 Panel A is very difficult for me to understand. Are the small RNA clusters under significant evolutionary selection? Are the small RNAs arising from the same lncRNA, significantly correlated in expression, with each other AND with the host transcript? Figure 3 contains promising analysis, but it is discussed in such a cursory way in the Legends and in the Results that it is difficult for me to interpret the results.

***Author's response:**** We thank the reviewer for the suggestion. In fact, we did not perform the expression analysis. Rather, in Figure*3*(Figure*1* in revised manuscript), we have plotted the read numbers or tag counts contributing to each of the clusters, which is a correlate for expression level of the small RNA. We could not find the expression level of the host lncRNAs for the same tissues which precludes the expression level comparison of lncRNA with small RNA. There have been known biases in small RNA sequencing (Hafna 2011) which precludes comparison of expression levels between small RNA. This could be circumvented by generating experimental data for small RNA and lncRNAs at same tissue and/or time points. The legend for the figure has been modified in the revised manuscript to make the figure comprehensive.*

Small comments:

1. Abstract: “Sketchy” is a colloquial word that is not suited to scientific articles.

***Author's response:**** The abstract has been modified and improved as suggested by the reviewer.*

2. Throughtout: Probably better to say “Non-protein coding” rather than “non protein coding”.

***Author's response:**** As suggested by the reviewer “non protein coding” has been replaced by “non-protein coding/ non-coding” throughout the manuscript.*

3. Page 3, “majorly anecdotal” – this is not correct English, and furthermore not accurate: scientific results are not “anecdotal”, since they are backed up by experimental results and peer reviewed. Perhaps the authors meant to say conjectural”?

***Author's response:**** As pointed out by the reviewer the language has been modified.*

4. Page 4 “implicated is through recruiting chromatin modifiers”– needs citation.

***Author's response:**** We have modified the manuscript with citations to the statement*.

5. Page 4: “a transcript specified both an informational molecule as well as a structural molecule” – should cite SRA1 (Lanz et al.), the best studied (indeed, only) bifunctional RNA to date.

***Author's response:**** We thank the reviewer for the suggestion. We have included the citation in the revised version.*

6. Page 5: the authors repeat twice about 30 lncRNAs and 69 small RNAs.

***Author's response:**** The repetition has been corrected in the revision.*

7. Page 5: Are any of the small RNAs discovered in this analysis, known RNAs such as catalogued microRNAs or snoRNAs?

***Author's response:**** We thank the reviewers for the suggestion. In our initial analysis, where we considered lncRNAdb data, 9 clusters were catalogued as 41 pasRNAs (from deepBase) and one of the small RNA cluster (chr11_rcluster204) discovered, is catalogued as miRNA (from miRBase) i.e. hsa-mir-675. While in our Gencode dataset we found 12 miRNAs, 695 nasRNAs and 1052 pasRNAs in 12, 9 and 150 small RNA clusters respectively. We have compiled these results in Additional File**4**and also added these results in the revised manuscript.*

8. Table 1, Figure 2, Figure 3 – the data is presented with little useful detail (eg Table 1 – what are the Cluster locations and strand?, Figure 2, what are theactual numbers of RNAs, not %ages?) and the Figure Legends are way to short and lacking also useful details.

***Author's response:**** As suggested by the reviewer Table 1 (Additional File**2**in revised manuscript) has been modified and 2 additional columns are added i.e. Cluster locations and strand in addition to the existing columns “lncRNA Name”, “Genomic Position”, “Length of lncRNA” and “deepBase Clusters”. We hope this would serve as a ready reference and starting point for experimental validation of interesting candidates. We have also revised the figure legends with more details as suggested.*

9. Supplementary Figure 3 seems to be identical to main Figure 3.

***Author’s response:**** In revised manuscript, the Supplementary Figure*3*, is placed as Additional File**6**and Figure*3*, is now Figure*1*.*

10. In general, I do not find the Supplementary Files particularly useful or relevant to the paper.

***Author’s response:**** The Supplementary Files have been modified in the revised manuscript. We have only included Tables and Figures relevant to the paper and removed the redundant ones.*

I thank the Authors for responding comprehensively to my comments. I continue to have doubts about the value of this paper. I have the impression that the analysis and manuscript preparation has been carried in a rather careless way, without consideration of the implications of the work, or for with clarity for the reader. Furthermore, I am concerned by the lack of clarity from the Authors about whether the claimed small RNA overlaps are occurring on the same strand as the claimed host lncRNA - an issue of crucial importance to this manuscript.

***Author's response:**** We have analyzed the smallRNA clusters as annotated by DeepBase and falling in the same orientation as the lncRNA. The manuscript section has been modified appropriately to detail the analysis methodology. We agree with the reviewer that the computational analysis does not provide much insight into the potential biological implications of the observation. In fact, in present situation, our understanding of biological functions for majority of lncRNAs is not known and computational methods to functionally assign roles are still naïve. The present report serves as a starting point and ready reference to a dataset which suggests that a subset of lncRNAs could potentially be processed to smaller RNAs.*

*In the revised manuscript, we detail our observation on a relatively well studied lncRNA. The lncRNA PTENTP1 is a pseudogene of PTEN gene. Our analysis reveals that PTENP1 harbors 5 small RNAs clusters as annotated by deepBase. This observation is also corroborated by independent dataset of small RNA cloning data from smiRNAdb*[33]*which revealed that the fifth cluster comprises of three distinct small RNA clusters, having differential expression levels in different tissues as depicted in Figure*1*. This could lead to a possibility whereby apart from the PTENP1 function; the processed small RNAs could be an additional mechanism for modulating biological processes in the cell and potentially in the pathogenesis of oncogenesis.*

*We have compiled our results in tabular format which is available along with manuscript as additional files (Additional File*2* and Additional File*3*), where we have mentioned that small RNAs are mapped onto the sense strand of lncRNAs.*

While the topic of lncRNA processing into small RNAs is an interesting and timely one, the present manuscript does little to address to me the fundamental questions in the field: Are lncRNAs processed to small RNAs? Do these arise preferentially from exons or introns? Does this process happen at a rate that exceeds background chance? Do these small RNAs show any evidence for function (ie evolutionary selection)? Do these small RNAs have interesting and reasonable tissue expression profiles? Are these small RNAs marked by any kind of epigenetic signal? etc. etc.

Quality of written English: Not suitable for publication unless extensively edited.

***Author's response:**** This manuscript is based on the hypothesis that a subset of lncRNAs could be processed into small RNAs thereby also potentially contributing to independent functions. In our analysis, we were able to computationally map smallRNA datasets and suggest that a subset of lncRNAs could be potentially processed to smallRNAs. The present analysis with its limitations cannot exclude this being an independent event nor can assign potential biological implications to this observation. Our observation also reveals that there is a preference of the smallRNAs to map to the exons of lncRNAs. Additional analysis data suggest this is a regulated process, with differential expression in tissues. This makes us argue that this could suggest a coordinated and regulated process of biogenesis rather than a degradation process, though at this moment we lack hard evidence to prove or disprove this possibility. We report his observation with adequate datasets to support further in-depth analysis and experiments in this direction. We are also aware of epigenetic signals which both mark genes as well as regulate gene expression. We have indeed performed extensive analysis in these lines, but are clearly beyond the scope of this manuscript.*

I thank the authors for putting so much work into addressing my concerns.

Technical Comments:

- The authors have now provided a complete dataset of small RNA clusters overlapping Gencode LncRNAs. This file is Supplementary File 3. There are some issues still:

1) The file is likely to be used by bioinformaticians, so that a text file / tab delimited file would be more practical than a pdf.

2) Even in cases where multiple clusters overlap one lncRNA, a single location for the clusters is given, whereas it is necessary to specify the locations and IDs of ALL the clusters.

3) The location given for the lncRNA is "exon positions". It would more logical to provide thestart and end position of the RNA transcript.

- Figure 1 remains a crude screen shot from Ensembl that is pretty much incomprehensible to me.

- It is nice that the authors estimated the overlap rate of small RNA clusters in lncRNA exons and introns. An additional good control would be counting the rate of overlap of small RNA clusters on the OPPOSITE strand of lncRNA exons and introns.

Overall I still have doubts about the value of this work to the scientific community, as it presently stands - which is unfortunate because the authors have identified a very timely contemporary question in the field: whether and which lncRNAs serve as precursors to small RNAs. However, this paper does still not address this question. The question it addresses is "how many small RNA clusters overlap lncRNAs". We do not know whether the small RNA clusters originate from expression of the long RNA. The authors do not seem to make much effort to check this, which is a shame because with the wealth of available data (including RNAseq from long and small RNA that has been available from ENCODE through the UCSC genome browser for quite some time) this question is eminently answerable.

Quality of written English: Acceptable

### Reviewer’s Report

**Title:** Integrative transcriptomes analysis suggest processing of a subset of long non-codingRNAs to small RNAs

**Versions:** 1 & 2 6 February 2012.

**Reviewer Number:** 2.

**Reviewer:** Dr Raya Khanin (nominated by Dr Yuriy Gusev).

This paper proposed a interesting hypothesis for a novel function of a subset of lncRNAs that are processed into microRNAs. While the idea is plausible, the study requires additional analysis. In particular, considering additional lncRNAs databases and performing statisticalanalysis when making statements about significance.

**Quality of written English:** Acceptable

***Author's response:**** We thank the reviewer for the suggestion. We have performed analysis on an independent and larger dataset of annotated lncRNAs. Please also refer to comments of reviewer 1. We have revised the manuscript with specific suggestions in the same line, as suggested by the other reviewers.*

### Reviewer’s Report

**Title:** Integrative transcriptomes analysis suggest processing of a subset of long non-codingRNAs to small RNAs

**Versions:** 1, 2 & 3 7 February 2012/ 30 March 2012/19 June 2012.

**Reviewer Number:** 3.

**Reviewer:** Prof Neil Smalheiser.

This paper documents that many small RNAs are derived from ncRNAs, including those that are relatively well studied. This is not surprising, given the pervasive transcription of the genome, and given the many classes of small RNAs that have already been described. To optimize the usefulness of this report for other scientists, the current findings are vague and incomplete, and should be presented in far more detail, and characterized much further.

At the simplest level, the composition of the small RNA clusters is not clear. Are "small RNAs" all in the size range of microRNAs, i.e. 18–24 nt,? of the size of piRNAs? or even much larger, up to 50 or 100 nt? Are repeat elements removed from the set, or not? (Note that BC200, one of the listed ncRNAs, is derived from an Alu element.) Would the results be similar, or very different, if each of these classes were examined separately in relation to ncRNAs? Are the small RNAs biased in any fashion (e.g. were adaptor strategies designed to capture RNAseIII products preferentially)? (I looked at the deepBase reference and was still not entirely sure what types of small RNAs were included in clusters and what, if any, were excluded.)

***Author's response:**** We thank the reviewer for the suggestion. We have plotted a size distribution graph for the small RNA clusters derived from deepBase mapping to lncRNA exons from Gencode dataset (Additional File**7**). Analysis reveals the mean footprint of the cluster to be 105.25 bases and median as 86. By definition, this potentially is much lesser than the generally accepted criteria for long non-coding RNAs, which are by definition > 200 bp. deepBase contains 4,08,009 of small RNAs clusters derived from known ncRNAs, protein-coding genes and repeat elements, as well as a large number of un-annotated small RNAs. We have examined the presence of repeat elements in the deepBase small RNA clusters which mapped onto lncRNA exons and analysis revealed 113 small RNA clusters catalogued as 78 repeat elements.*

*As per the deepBase manuscript*[24]*, the data was derived from 9 cell lines. The data was downloaded from the NCBI GEO database and further processed. The reads were clipped for adapters using in-house Perl scripts. Upon removal of adapters, the sequences shorter than 15 nt were discarded.*

The paper should not only have some hypothesis that is being assessed, but some consideration of alternative hypotheses. Is it possible that many or even most of the small RNAs are degradation products of the ncRNAs? The mere observation that small RNA levels vary in different cell types does not imply biological function, since ncRNAs may be expressed at varying levels in different cell types followed by nonspecific degradation. It would be much better to identify other reasons for thinking that some of the small RNAs are functional.

***Author's response:**** In the current study we hypothesise and provide evidence suggesting that a subset of lncRNAs could be potentially processed to smaller RNAs. We agree with the reviewer that the existence of small RNAs or non-uniform processing of the lncRNA to small RNAs do not imply biological function. The datasets available at this point essentially are not sufficient to assign any biological function to the small RNAs, and as a matter of fact, the functions of a large number of long non-coding RNAs are not well understood. Our analysis does not preclude the possibility of the small RNA cluster being formed by degradation products, as differential stability could also contribute to differential tag counts as in Figure*1*, though non-random distribution of the clusters and stringent criteria for calling small RNA clusters by deepBase suggests a possible co-ordinated mechanism of processing. Nevertheless, we hope this analysis would be a starting point to direct further experimental investigation into possible functional roles of this observation.*

The characterization of alignments is also not clear. Saying that "45% of sequences map to the 3'- third of sequences" is not a particularly precise statement and does not give much insight. A much higher-resolution characterization would be helpful. Do the small RNAs arise from regions that have particular secondary structures? Are there any presumptive RNAse cleavage sites that generate them?

***Author's response:**** We have taken into consideration the reviewers’ suggestion in the revised manuscript. We have re-analysed the Gencode data with independent 5% bins across the lncRNA length. The revised Figure*2*depicts the distribution of reads across the Gencode set of lncRNAs. We have checked the existence of a potential sequence or structural motif around the processing site, but could not find any significant hit, which is not surprising, as previous studies have suggested that many features acting in combination mark RNA processing sites (Helvik et al., Bioinformatics (2007) 23 (2): 142–149.)*

Another ambiguity is that a given ncRNA, like any protein-coding RNA, may be the host gene for Drosha and/or Dicer processing to give rise to miRNAs. It is not clear to me whether a miRNA locus within a ncRNA would always be annotated as an “exon” or an “intron”. One well known ncRNA, BIC, is also a pri-miR forhsa-mir-155, and the pre-miR is annotated as lying within an “exon” (on the UCSC Genome Browser). How much do known miRNAs affect your analysis? The same exon of BIC contains a MIR repeat element. How much do known repeat elements within ncRNAs affect your analysis?

***Author’s response:**** We thank the reviewers for the suggestion. In our initial analysis where we considered lncRNAdb data, 9 clusters were catalogued as 41 pasRNAs (from deepBase) and one of the small RNA cluster (chr11_rcluster204) discovered is catalogued as miRNA (from miRBase) i.e. hsa-mir-675. While in our Gencode dataset we found 12 miRNAs, 695 nasRNAs and 1052 pasRNAs in 12, 9 and 150 small RNA clusters respectively. We have compared and mapped known miRNA loci in lncRNAs. Several other cases of miRNAs now being annotated at lncRNA loci has been reported*[45]*suggesting this could be more frequent event then previously known. The results section has been detailed in the revised manuscript with additional data (Additional File**4**).*

To conclude, I found it difficult to get a mental picture of what kinds of small RNAs map to ncRNAs, and how they map. This should be clarified, first, and then more attention should be given to identifying clues that would suggest what they might be doing.

***Author’s response:**** We have revised the manuscript to make it more readable and comprehensive. We have also provided additional analyses in the revised manuscript with a section on analysis of a independent dataset of lncRNAs and potential overlaps with other classes of annotated non-coding small RNA classes.*

Specific comments: Figure 3 is very hard to read, and supplement 3 is missing (Figure 3 is repeated instead) ***Author’s response:****We have modified the legends in the revised manuscript to make the figure more legible. In the revised manuscript, Supplementary Figure*3*, is placed as Additional File*6* and Figure*3*, is now Figure*1*.*

This manuscript is not noticeably improved from the first submission, is no easier to read or understand, and does not provide enough detailed analysis to give useful biological insights. No sequences of specific small RNAs are displayed in the paper, and no specific example is analyzed in any depth. The authors have added Gencode data, but this only adds to the size of the raw data and does not move the ms. forward substantially. The Results section is impossible to understand (for example, small RNA clusters are not defined or characterized) without reading the Methods first. I recommend that the authors carry out further biologically-oriented analysis and not publish the manuscript in its current form.

### Quality of written English: Acceptable

***Author’s response:**** In the present manuscript, we propose that lncRNAs process into small RNAs thereby showing dual regulatory functions. We have tried to provide biological insights by detailing one such candidate in the revised manuscript. We have shown that well known lncRNA (PTENTP1) seems to harbor small RNA, PTENP1 is a pseudogene of PTEN gene. Our analysis shows that PTENP1 harbors 5 small RNAs clusters as annotated by deepBase. We also mapped small RNA cloning data from smiRNAdb*[33]*which revealed that the fifth cluster comprises of three distinct small RNA clusters, having differential expression levels in different tissues as depicted in Figure*1*. This could lead to a possibility whereby apart from the PTENP1 function; the processed small RNAs could be an additional mechanism for modulating biological processes in the cell and potentially in the pathogenesis of oncogenesis.*

We understand the limitations of computational analysis exclusively based on datasets available in public domain and also understand that only a very miniscule number of lncRNAs have been assigned any biological function. Furthermore, the computational tools and methodologies to assign biological functions to lncRNAs are still naïve. Rather than detailing the biological significance of each lncRNA and smallRNA cluster, we would rather like this report to serve as a ready reference and starting point for experimental validation of interesting candidates. We have modified the language of manuscript to make it more readable and comprehensive. To this end, we have included analysis on a larger and independent dataset of lncRNAs available from Gencode. In fact this analysis was performed as suggested by the first reviewer. Please also refer to comments to reviewer 1.

I have looked at this "revised" manuscript and still do not see substantial improvement. I do not care to make any public comments at this point, since the online review form does not allow for comments to the editor only. I stand by my earlier suggestion that the authors defer publication, but of course they can do as they wish.

## Competing interests

The authors declare that they have no conflicting interests.

## Authors’ contributions

SJ, GGJ and VS planned the experiment. SJ and GGJ performed the analysis. All authors contributed to writing the manuscript. All authors read and approved the final manuscript.

## Supplementary Material

Additional file 1 Graph depicting the length distribution of lncRNAs derived from lncRNAdb. Click here for file

Additional file 2 Tabular summary of lncRNAs and small RNA clusters mappings to lncRNA exons derived from lncRNAdb. Click here for file

Additional file 3 Tabular summary of lncRNAs and small RNA clusters mapping to lncRNA exons derived from Gencode database. Click here for file

Additional file 4 Table summarizing small RNA clusters discovered in our analysis, catalogued as miRNA, pasRNA and nasRNA; A) small RNA clusters mapped to lncRNA exons from lncRNAdb catalogued as different types of small RNAs B) small RNA clusters mapped to lncRNA exons from Gencode database catalogued as different types of small RNAs. Click here for file

Additional file 5 Pie chart depicting the distribution of small RNA clusters in the 5’ Region, 3’ Region and Mid Region along the length of lncRNAs: A) Lengths of lncRNAs derived from lncRNAdb B) Lengths of lncRNAs derived from Gencode database. Click here for file

Additional file 6 The DOC file containing the mapping of small RNA clusters in Tug1 lncRNA derived from lncRNAdb. Click here for file

Additional file 7 Graph showing the size distribution of deepBase clusters mapped to lncRNA exons from Gencode dataset. Click here for file
