# Supplementary figures and images for: Integrative transcriptome analysis suggest processing of a subset of long non-coding RNAs to small RNAs
Source: Biol Direct. 2012 Aug 7;7:25. doi: 10.1186/1745-6150-7-25 (PMC3477000; doi:10.1186/1745-6150-7-25)

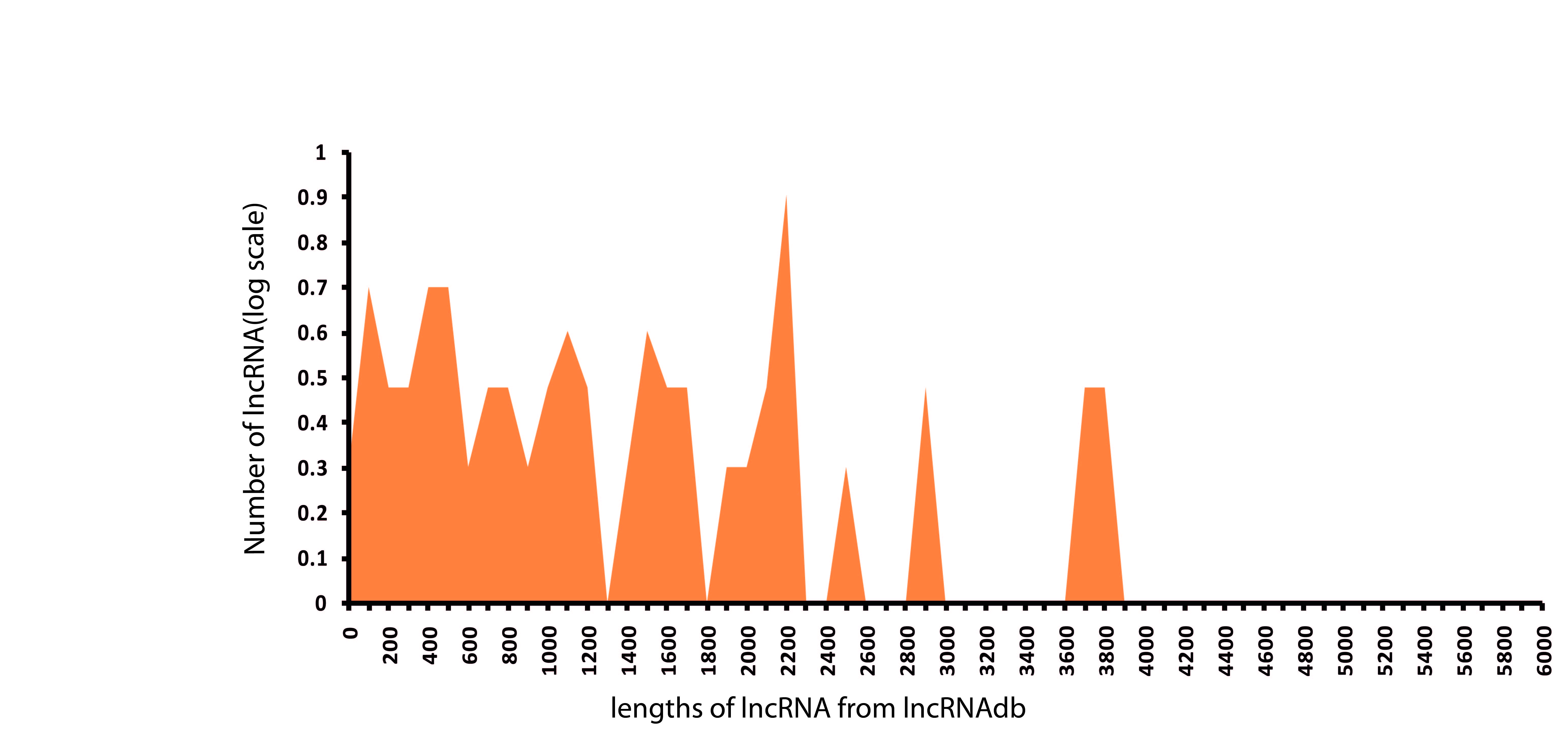

Supplement: Additional file 1 — Graph depicting the length distribution of lncRNAs derived from lncRNAdb. [file 1745-6150-7-25-S1.doc]

**[A] [B]**


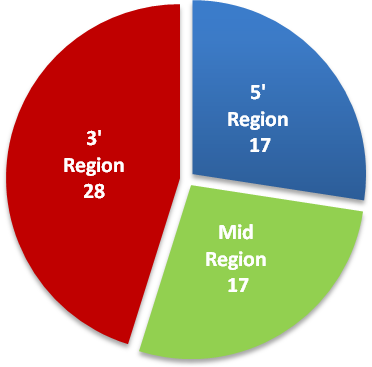

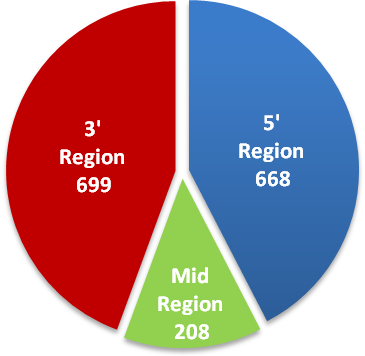

Supplement: Additional file 5 — Pie chart depicting the distribution of small RNA clusters in the 5’ Region, 3’ Region and Mid Region along the length of lncRNAs: A) Lengths of lncRNAs derived from lncRNAdb B) Lengths of lncRNAs derived from Gencode database. [file 1745-6150-7-25-S5.doc]

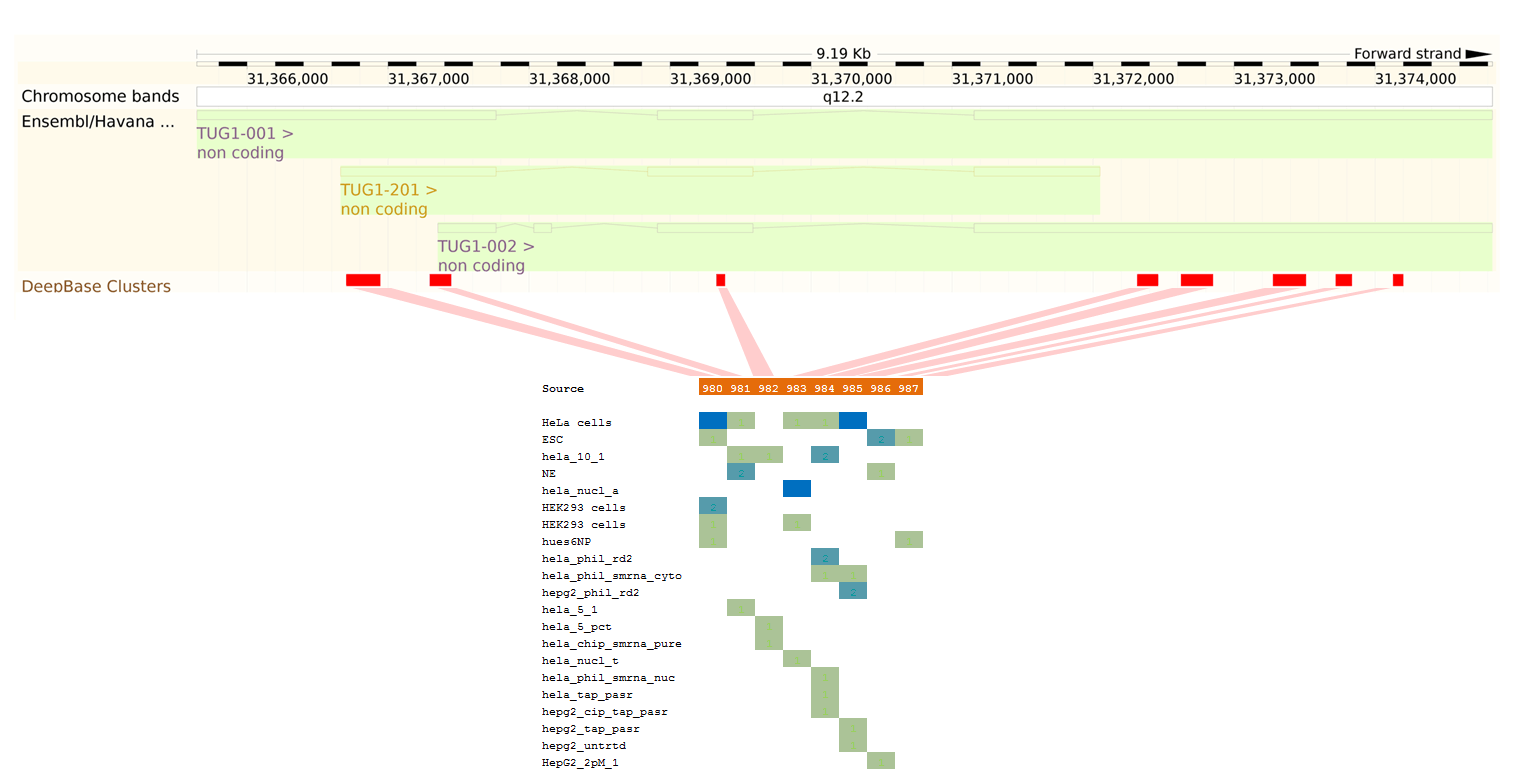

Supplement: Additional file 6 — The DOC file containing the mapping of small RNA clusters in Tug1 lncRNA derived from lncRNAdb. [file 1745-6150-7-25-S6.doc]

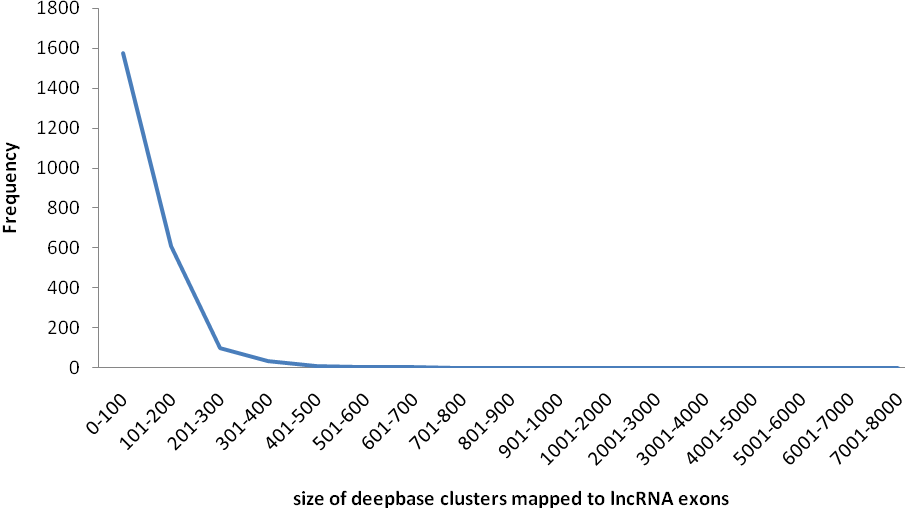

Supplement: Additional file 7 — Graph showing the size distribution of deepBase clusters mapped to lncRNA exons from Gencode dataset. [file 1745-6150-7-25-S7.doc]
