# Supplementary material for: Integrative transcriptome analysis suggest processing of a subset of long non-coding RNAs to small RNAs
Source: Biol Direct. 2012 Aug 7;7:25. doi: 10.1186/1745-6150-7-25 (PMC3477000; doi:10.1186/1745-6150-7-25)
Supplement: Additional file 2 — Tabular summary of lncRNAs and small RNA clusters mappings to lncRNA exons derived from lncRNAdb. [file 1745-6150-7-25-S2.doc]

| **lncRNA  Name** | **Genomic  Position** | **Length of lncRNA** | **DeepBase Clusters** | **Cluster  Location** | **Strand** |
| --- | --- | --- | --- | --- | --- |
| **BC200** | chr2:47562453-47562653 | 200 | 1 | 47562532-47562640 | + |
| **Beta-globin2** | chr11:5304739-5305929 | 410 | 1 | 5305597-5305713 | - |
| **Beta-globin3** | chr11:5304904-5305952 | 1047 | 1 | 5305597-5305713 | - |
| **DHFR** | chr5:79950178-79950765 | 587 | 1 | 79950484-79950566 | + |
| **Dio3os** | chr14:102023640:102026759 | 1746 | 1 | 102025054-102025117 | - |
| **Gomafu** | chr22: 27053445-27072441 | 10193 | 5 | | 27067580-27067633 | | --- | | 27069931-27069991 | | 27069022-27069095 | | 27068382-27068460 | | 27069508-27069587 | | + |
| **H19** | chr11:2016405-2019065 | 2322 | 2 | | 2017995-2018052 | | --- | | 2018197-2018256 | | - |
| **HOTAIRM1** | chr7:27135712-27139585 | 483 | 1 | 27135830-27135883 | + |
| **IPW** | chr15:25361691-25367623 | 4498 | 3 | | 25365321-25365398 | | --- | | 25364818-25364907 | | 25364402-25364530 | | + |
| **Jpx** | chrX:73164158-73290243 | 1696 | 2 | | 73290046-73290119 | | --- | | 73164168-73164283 | | + |
| **KRASP1** | chr6:54635168-54636037 | 865 | 1 | 54635712-54635768 | + |
| **LOC285194** | chr3:116428634-116435887 | 2105 | 2 | | 116431356-116431413 | | --- | | 116428992-116429086 | | + |
| **Malat1** | chr11:65265232-65273940 | 8708 | 3 | | 65273801-65273851 | | --- | | 65265480-65265577 | | 65266515-65273645 | | + |
| **MEG3** | chr14:101292444-101327363 | 1855 | 1 | 101298872-101298947 | + |
| **NEAT1** | chr11: 7590863-65190268 | 3756 | 3 | | 65192146-65192493 | | --- | | 65191426-65192076 | | + |
| **PCGEM1** | chr2:193614570-193641625 | 1603 | 1 | 193641311-193641382 | + |
| **PR** | chr11:100999807-101069641 | 1532 | 1 | 100999884-100999958 | + |
| **PTENP1** | chr9:33673501-33677418 | 3932 | 5 | | 33673901-33673993 | | --- | | 33674594-33674757 | | 33676513-33676683 | | 33675631-33675815 | | 33677005-33677278 | | - |
| **SNHG1** | chr11:62619459-62623360 | 1134 | 1 | 62619731-62619778 | - |
| **SNHG3** | chr1:28832454-28837404 | 2346 | 2 | | 28835883-28835977 | | --- | | 28836971-28837114 | | + |
| **SNHG4** | chr5:138609440-138615317 | 1100 | 1 | 138614909-138614967 | + |
| **SNHG6** | chr8:67834164-67837777 | 472 | 1 | 67834181-67834348 | - |
| **ST7OT1** | chr7:116592499-116594388 | 1908 | 3 | | 116593666-116593717 | | --- | | 116593972-116594053 | | 116594287-116594377 | | - |
| **ST7OT4** | chr7:116752345-116785614 | 1589 | 2 | | 116593960-116594015 | | --- | | 116599424-116599507 | | + |
| **TUG1** | chr22:31365633-31375381 | 7115 | 8 | | 31369328-31369389 | | --- | | 31374127-31374199 | | 31373720-31373835 | | 31372312-31372460 | | 31367295-31367447 | | 31372623-31372849 | | 31373275-31373509 | | 31366703-31366944 | | + |
| **UM9-5** | chr4:21844963-21854811 | 9848 | 1 | 21854696-21854744 | - |
| **Xist** | chrX:73040494-73072588 | 19271 | 10 | | 73071436-73071488 | | --- | | 73044219-73044278 | | 73068927-73068986 | | 73040992-73041057 | | 73069935-73070023 | | 73070984-73071077 | | 73041445-73041542 | | 73071201-73071300 | | 73061551-73061674 | | 73045091-73045245 | | - |
| **Zeb2NAT** | chr2:145275981-145278683 | 1187 | 1 | 145278017-145278192 | + |
| **Zfas1** | chr20:47894714-47905797 | 1020 | 2 | | 47894737-47894841 | | --- | | 47905582-47905768 | | + |
