# Supplementary material for: Integrative transcriptome analysis suggest processing of a subset of long non-coding RNAs to small RNAs
Source: Biol Direct. 2012 Aug 7;7:25. doi: 10.1186/1745-6150-7-25 (PMC3477000; doi:10.1186/1745-6150-7-25)
Supplement: Additional file 3 — Tabular summary of lncRNAs and small RNA clusters mapping to lncRNA exons derived from Gencode database. [file 1745-6150-7-25-S3.doc]

| **lncRNA transcripts** | **Exonic Positions** | **strand** | **Cluster_Name** | **Cluster_location** | **deepbase clusters** |
| --- | --- | --- | --- | --- | --- |
| ENST00000536678.1 | chr1:2113234-2115492 | - | chr1_rcluster313 | 2114762-2114807 | 1 |
| ENST00000381475.3 | chr1:147850201-147851698 | - | chr1_rcluster7407 | 147851091-147851137 | 3 |
| ENST00000413074.1 | chr1:222763305-222763749 | + | chr1_fcluster12040 | 222763410-222763456 | 2 |
| ENST00000445753.1 | chr1:143662699-143664196 | - | chr1_rcluster6982 | 143663589-143663635 | 121 |
| ENST00000480336.1 | chr1:33065689-33066393 | + | chr1_fcluster2346 | 33066114-33066160 | 2 |
| ENST00000504773.1 | chr1:201984015-201984785 | + | chr1_fcluster10935 | 201984652-201984698 | 4 |
| ENST00000417047.1 | chr1:249153364-249153445 | + | chr1_fcluster13491 | 249153397-249153444 | 3 |
| ENST00000428687.1 | chr1:247373994-247374295 | - | chr1_rcluster13204 | 247374082-247374129 | 4 |
| ENST00000443284.1 | chr1:55353237-55353456 | + | chr1_fcluster3829 | 55353277-55353324 | 2 |
| ENST00000473429.1 | chr1:110593579-110593682 | + | chr1_fcluster6279 | 110593629-110593676 | 2 |
| ENST00000369381.2 | chr1:142712962-142714605 | - | chr1_rcluster6936 | 142713843-142713891 | 2 |
| ENST00000412204.1 | chr1:143209431-143211070 | - | chr1_rcluster6956 | 143210312-143210360 | 2 |
| ENST00000428624.1 | chr1:143377282-143378913 | + | chr1_fcluster7079 | 143377987-143378035 | 2 |
| ENST00000445317.1 | chr1:2983902-2984612 | - | chr1_rcluster425 | 2984544-2984592 | 2 |
| ENST00000412962.1 | chr1:16952849-16952994 | - | chr1_rcluster1324 | 16952878-16952927 | 6 |
| ENST00000417659.1 | chr1:700104-700305 | - | chr1_rcluster75 | 700243-700292 | 2 |
| ENST00000421700.1 | chr1:16952849-16952994 | - | chr1_rcluster1324 | 16952878-16952927 | 6 |
| ENST00000428504.1 | chr1:700238-700627 | - | chr1_rcluster75 | 700243-700292 | 2 |
| ENST00000448869.1 | chr1:156616300-156616946 | - | chr1_rcluster8503 | 156616447-156616496 | 4 |
| ENST00000453837.1 | chr1:32307621-32309748 | - | chr1_rcluster2416 | 32308291-32308340 | 2 |
| ENST00000458151.1 | chr1:53704283-53705017 | + | chr1_fcluster3735 | 53704392-53704441 | 3 |
| ENST00000540383.1 | chr1:16952849-16952994 | - | chr1_rcluster1324 | 16952878-16952927 | 6 |
| ENST00000412344.1 | chr1:230846445-230846599 | - | chr1_rcluster12249 | 230846549-230846599 | 3 |
| ENST00000416689.1 | chr1:43439646-43442099 | + | chr1_fcluster3099 | 43441776-43441826 | 2 |
| ENST00000420350.1 | chr1:224415129-224415745 | + | chr1_fcluster12171 | 224415251-224415301 | 2 |
| ENST00000452996.1 | chr1:147816358-147816764 | + | chr1_fcluster7840 | 147816433-147816483 | 10 |
| ENST00000421202.1 | chr1:93802935-93804831 | - | chr1_rcluster5565 | 93803054-93803106 | 3 |
| ENST00000432741.1 | chr1:93802935-93803122 | - | chr1_rcluster5565 | 93803054-93803106 | 3 |
| ENST00000435793.1 | chr1:243260177-243260601 | - | chr1_rcluster12916 | 243260346-243260399 | 5 |
| ENST00000438255.1 | chr1:243242160-243242354 | - | chr1_rcluster12912 | 243242289-243242342 | 11 |
| ENST00000392963.3 | chr1:16888819-16890681 | - | chr1_rcluster1297 | 16890187-16890241 | 3 |
| ENST00000401007.3 | chr1:16888924-16890681 | - | chr1_rcluster1297 | 16890187-16890241 | 3 |
| ENST00000443207.1 | chr1:236121180-236121303 | - | chr1_rcluster12578 | 236121240-236121294 | 3 |
| ENST00000325963.7 | chr1:149261317-149265510 | + | chr1_fcluster7988 | 149263328-149263383 | 4 |
| ENST00000415338.1 | chr1:149378587-149379646 | - | chr1_rcluster7661 | 149378935-149378990 | 2 |
| ENST00000428289.1 | chr1:149763515-149764576 | - | chr1_rcluster7702 | 149763863-149763918 | 2 |
| ENST00000445225.1 | chr1:149646919-149651107 | + | chr1_fcluster8030 | 149648925-149648980 | 4 |
| ENST00000457996.1 | chr1:120925635-120926696 | + | chr1_fcluster6870 | 120926291-120926346 | 2 |
| ENST00000537401.1 | chr1:120924842-120926874 | + | chr1_fcluster6870 | 120926291-120926346 | 2 |
| ENST00000417084.1 | chr1:207481813-207482466 | + | chr1_fcluster11320 | 207482351-207482408 | 2 |
| ENST00000452971.1 | chr1:38940869-38941496 | + | chr1_fcluster2710 | 38941367-38941425 | 3 |
| ENST00000468030.1 | chr1:145281370-145281704 | + | chr1_fcluster7384 | 145281646-145281704 | 4 |
| ENST00000416512.1 | chr1:32827332-32829824 | - | chr1_rcluster2468 | 32827696-32827755 | 8 |
| ENST00000432622.1 | chr1:32827560-32827844 | - | chr1_rcluster2468 | 32827696-32827755 | 8 |
| ENST00000439341.1 | chr1:231014539-231014761 | + | chr1_fcluster12664 | 231014698-231014758 | 4 |
| ENST00000453618.1 | chr1:145115736-145116922 | + | chr1_fcluster7369 | 145116573-145116634 | 2 |
| ENST00000411941.1 | chr1:144506278-144506436 | - | chr1_rcluster7053 | 144506282-144506344 | 2 |
| ENST00000412194.1 | chr1:144325840-144326185 | - | chr1_rcluster7031 | 144326031-144326093 | 2 |
| ENST00000424528.1 | chr1:98511565-98511952 | - | chr1_rcluster5754 | 98511643-98511705 | 2404 |
| ENST00000433598.1 | chr1:144326027-144326185 | - | chr1_rcluster7031 | 144326031-144326093 | 2 |
| ENST00000435404.1 | chr1:144506278-144506436 | - | chr1_rcluster7053 | 144506282-144506344 | 2 |
| ENST00000440719.1 | chr1:144326027-144326185 | - | chr1_rcluster7031 | 144326031-144326093 | 2 |
| ENST00000444883.1 | chr1:144506092-144506436 | - | chr1_rcluster7053 | 144506282-144506344 | 2 |
| ENST00000445225.1 | chr1:149646919-149651107 | + | chr1_fcluster8031 | 149650175-149650237 | 3 |
| ENST00000452356.1 | chr1:149591079-149591237 | + | chr1_fcluster8021 | 149591169-149591231 | 2 |
| ENST00000455295.1 | chr1:149591079-149591238 | + | chr1_fcluster8021 | 149591169-149591231 | 2 |
| ENST00000421703.1 | chr1:183439856-183440881 | - | chr1_rcluster9743 | 183440220-183440283 | 4 |
| ENST00000430580.1 | chr1:16939834-16940083 | - | chr1_rcluster1320 | 16939986-16940049 | 8 |
| ENST00000451992.1 | chr1:167596623-167598884 | + | chr1_fcluster9315 | 167597565-167597628 | 2 |
| ENST00000532582.1 | chr1:183439156-183441400 | - | chr1_rcluster9743 | 183440220-183440283 | 4 |
| ENST00000436991.1 | chr1:25971036-25972911 | - | chr1_rcluster1974 | 25972293-25972358 | 3 |
| ENST00000441724.1 | chr1:6296301-6296396 | + | chr1_fcluster450 | 6296322-6296387 | 2 |
| ENST00000449853.1 | chr1:16931914-16934186 | - | chr1_rcluster1318 | 16933203-16933268 | 2 |
| ENST00000413897.1 | chr1:143188510-143188628 | - | chr1_rcluster6954 | 143188522-143188588 | 2 |
| ENST00000420598.1 | chr1:143188510-143188589 | - | chr1_rcluster6954 | 143188522-143188588 | 2 |
| ENST00000437267.1 | chr1:143188510-143188628 | - | chr1_rcluster6954 | 143188522-143188588 | 2 |
| ENST00000366437.3 | chr1:209602169-209602433 | + | chr1_fcluster11426 | 209602270-209602337 | 2 |
| ENST00000414532.1 | chr1:31191352-31194021 | + | chr1_fcluster2211 | 31191597-31191664 | 2 |
| ENST00000429156.1 | chr1:209602166-209602433 | + | chr1_fcluster11426 | 209602270-209602337 | 2 |
| ENST00000431096.1 | chr1:209602184-209602433 | + | chr1_fcluster11426 | 209602270-209602337 | 2 |
| ENST00000433108.1 | chr1:209602166-209605170 | + | chr1_fcluster11426 | 209602270-209602337 | 2 |
| ENST00000451937.1 | chr1:209602206-209602753 | + | chr1_fcluster11426 | 209602270-209602337 | 2 |
| ENST00000426533.1 | chr1:143687159-143687309 | + | chr1_fcluster7093 | 143687191-143687259 | 5 |
| ENST00000432384.1 | chr1:143687098-143687309 | + | chr1_fcluster7093 | 143687191-143687259 | 5 |
| ENST00000457439.1 | chr1:143687153-143687309 | + | chr1_fcluster7093 | 143687191-143687259 | 5 |
| ENST00000432537.1 | chr1:63624708-63624941 | - | chr1_rcluster4288 | 63624735-63624804 | 2 |
| ENST00000412962.1 | chr1:16944758-16946519 | - | chr1_rcluster1321 | 16945141-16945211 | 2 |
| ENST00000421147.1 | chr1:222141691-222141838 | + | chr1_fcluster12013 | 222141749-222141819 | 7 |
| ENST00000427290.1 | chr1:112719857-112720235 | - | chr1_rcluster6384 | 112720132-112720202 | 2 |
| ENST00000430114.1 | chr1:202830577-202830748 | - | chr1_rcluster10573 | 202830608-202830678 | 5 |
| ENST00000443294.1 | chr1:202830577-202830736 | - | chr1_rcluster10573 | 202830608-202830678 | 5 |
| ENST00000540383.1 | chr1:16944752-16945598 | - | chr1_rcluster1321 | 16945141-16945211 | 2 |
| ENST00000413332.1 | chr1:3816937-3817195 | + | chr1_fcluster337 | 3816956-3817027 | 13 |
| ENST00000435542.1 | chr1:208047141-208047428 | - | chr1_rcluster10891 | 208047294-208047365 | 2 |
| ENST00000480336.1 | chr1:32936186-32937216 | + | chr1_fcluster2334 | 32936609-32936680 | 6 |
| ENST00000452901.1 | chr1:83632388-83632498 | + | chr1_fcluster4977 | 83632394-83632466 | 2 |
| ENST00000457706.1 | chr1:21912966-21913582 | + | chr1_fcluster1506 | 21913199-21913272 | 2 |
| ENST00000424774.1 | chr1:16481707-16482250 | + | chr1_fcluster1141 | 16481724-16481798 | 5 |
| ENST00000432694.1 | chr1:172745046-172745267 | + | chr1_fcluster9568 | 172745059-172745133 | 3 |
| ENST00000445300.1 | chr1:8181535-8182762 | + | chr1_fcluster586 | 8181575-8181650 | 2 |
| ENST00000416385.1 | chr1:682076-685396 | - | chr1_rcluster71 | 682195-682271 | 2 |
| ENST00000484859.1 | chr1:146387-149707 | - | chr1_rcluster31 | 146506-146582 | 2 |
| ENST00000436031.1 | chr1:182517836-182520344 | + | chr1_fcluster10043 | 182520115-182520192 | 2 |
| ENST00000331856.2 | chr1:39989480-39991357 | - | chr1_rcluster2932 | 39991070-39991148 | 2 |
| ENST00000421703.1 | chr1:183439856-183440881 | - | chr1_rcluster9744 | 183440781-183440861 | 5 |
| ENST00000532582.1 | chr1:183439156-183441400 | - | chr1_rcluster9744 | 183440781-183440861 | 5 |
| ENST00000366527.3 | chr1:245003941-245008646 | - | chr1_rcluster13027 | 245006383-245006465 | 2 |
| ENST00000417262.1 | chr1:202780704-202781041 | + | chr1_fcluster10988 | 202780888-202780971 | 2 |
| ENST00000425295.1 | chr1:202780818-202781041 | + | chr1_fcluster10988 | 202780888-202780971 | 2 |
| ENST00000428747.1 | chr1:15653177-15656179 | - | chr1_rcluster1177 | 15654409-15654492 | 2 |
| ENST00000456414.1 | chr1:117035646-117037528 | - | chr1_rcluster6651 | 117035973-117036056 | 3 |
| ENST00000537401.1 | chr1:120924842-120926874 | + | chr1_fcluster6869 | 120925624-120925707 | 5 |
| ENST00000411760.1 | chr1:144481420-144481765 | - | chr1_rcluster7050 | 144481674-144481758 | 4 |
| ENST00000411795.1 | chr1:144301072-144301536 | - | chr1_rcluster7028 | 144301445-144301529 | 4 |
| ENST00000413356.1 | chr1:190768932-190770788 | + | chr1_fcluster10403 | 190769235-190769319 | 2 |
| ENST00000414119.1 | chr1:144301326-144301536 | - | chr1_rcluster7028 | 144301445-144301529 | 4 |
| ENST00000414344.1 | chr1:144481556-144481765 | - | chr1_rcluster7050 | 144481674-144481758 | 4 |
| ENST00000415386.2 | chr1:16860922-16862144 | - | chr1_rcluster1288 | 16861233-16861317 | 6 |
| ENST00000417986.1 | chr1:149615766-149615903 | + | chr1_fcluster8026 | 149615771-149615855 | 4 |
| ENST00000426786.1 | chr1:144481628-144481765 | - | chr1_rcluster7050 | 144481674-144481758 | 4 |
| ENST00000427204.1 | chr1:16861158-16861505 | - | chr1_rcluster1288 | 16861233-16861317 | 6 |
| ENST00000434047.1 | chr1:149615766-149615975 | + | chr1_fcluster8026 | 149615771-149615855 | 4 |
| ENST00000437785.1 | chr1:144481301-144481765 | - | chr1_rcluster7050 | 144481674-144481758 | 4 |
| ENST00000439332.1 | chr1:144300516-144301536 | - | chr1_rcluster7028 | 144301445-144301529 | 4 |
| ENST00000441423.1 | chr1:144301191-144301536 | - | chr1_rcluster7028 | 144301445-144301529 | 4 |
| ENST00000442509.1 | chr1:144480745-144481765 | - | chr1_rcluster7050 | 144481674-144481758 | 4 |
| ENST00000443884.1 | chr1:149615766-149616111 | + | chr1_fcluster8026 | 149615771-149615855 | 4 |
| ENST00000444581.1 | chr1:149615766-149616783 | + | chr1_fcluster8026 | 149615771-149615855 | 4 |
| ENST00000445225.1 | chr1:149615766-149616230 | + | chr1_fcluster8026 | 149615771-149615855 | 4 |
| ENST00000445300.1 | chr1:8086799-8087481 | + | chr1_fcluster577 | 8087165-8087249 | 8 |
| ENST00000457991.1 | chr1:149615766-149616111 | + | chr1_fcluster8026 | 149615771-149615855 | 4 |
| ENST00000453229.1 | chr1:63154154-63154323 | + | chr1_fcluster4144 | 63154192-63154277 | 2 |
| ENST00000426301.1 | chr1:144340527-144341077 | - | chr1_rcluster7040 | 144340812-144340898 | 6 |
| ENST00000451424.1 | chr1:20974809-20978686 | - | chr1_rcluster1616 | 20976924-20977010 | 2 |
| ENST00000453293.1 | chr1:149580747-149581061 | + | chr1_fcluster8018 | 149580928-149581014 | 2 |
| ENST00000458262.1 | chr1:144516420-144516729 | - | chr1_rcluster7056 | 144516460-144516546 | 2 |
| ENST00000442889.1 | chr1:6844719-6844903 | - | chr1_rcluster658 | 6844752-6844839 | 3 |
| ENST00000412092.1 | chr1:142621002-142621532 | + | chr1_fcluster7036 | 142621377-142621465 | 3 |
| ENST00000418238.1 | chr1:114355231-114355447 | + | chr1_fcluster6514 | 114355235-114355323 | 7 |
| ENST00000392963.3 | chr1:16888819-16890681 | - | chr1_rcluster1296 | 16889665-16889754 | 3 |
| ENST00000401007.3 | chr1:16888924-16890681 | - | chr1_rcluster1296 | 16889665-16889754 | 3 |
| ENST00000461448.1 | chr1:28905051-28905192 | - | chr1_rcluster2215 | 28905064-28905153 | 2 |
| ENST00000464612.1 | chr1:28905051-28905192 | - | chr1_rcluster2215 | 28905064-28905153 | 2 |
| ENST00000470977.1 | chr1:28905051-28905192 | - | chr1_rcluster2215 | 28905064-28905153 | 2 |
| ENST00000473798.1 | chr1:761587-762902 | - | chr1_rcluster91 | 762149-762238 | 3 |
| ENST00000474814.1 | chr1:28905052-28905192 | - | chr1_rcluster2215 | 28905064-28905153 | 2 |
| ENST00000475441.1 | chr1:28905051-28905192 | - | chr1_rcluster2215 | 28905064-28905153 | 2 |
| ENST00000481220.1 | chr1:28905054-28905192 | - | chr1_rcluster2215 | 28905064-28905153 | 2 |
| ENST00000481368.1 | chr1:28905053-28905192 | - | chr1_rcluster2215 | 28905064-28905153 | 2 |
| ENST00000483436.1 | chr1:28905059-28905192 | - | chr1_rcluster2215 | 28905064-28905153 | 2 |
| ENST00000488745.1 | chr1:28905054-28905192 | - | chr1_rcluster2215 | 28905064-28905153 | 2 |
| ENST00000531126.1 | chr1:28905051-28905192 | - | chr1_rcluster2215 | 28905064-28905153 | 2 |
| ENST00000536430.1 | chr1:761590-762886 | - | chr1_rcluster91 | 762149-762238 | 3 |
| ENST00000436642.1 | chr1:21059374-21059518 | + | chr1_fcluster1451 | 21059421-21059511 | 2 |
| ENST00000442130.1 | chr1:160232192-160232568 | + | chr1_fcluster8855 | 160232409-160232499 | 6 |
| ENST00000445070.1 | chr1:48284254-48285909 | + | chr1_fcluster3483 | 48285222-48285312 | 2 |
| ENST00000445118.1 | chr1:762989-763155 | + | chr1_fcluster47 | 763038-763128 | 5 |
| ENST00000445551.1 | chr1:47897806-47900313 | - | chr1_rcluster3523 | 47899311-47899401 | 2 |
| ENST00000454651.1 | chr1:201476370-201476547 | + | chr1_fcluster10873 | 201476387-201476477 | 3 |
| ENST00000468030.1 | chr1:145304448-145304652 | + | chr1_fcluster7408 | 145304562-145304652 | 4 |
| ENST00000421703.1 | chr1:183439856-183440881 | - | chr1_rcluster9742 | 183440043-183440134 | 2 |
| ENST00000457348.1 | chr1:203273982-203274437 | - | chr1_rcluster10607 | 203274334-203274425 | 10 |
| ENST00000532582.1 | chr1:183439156-183441400 | - | chr1_rcluster9742 | 183440043-183440134 | 2 |
| ENST00000423403.1 | chr1:61125304-61127142 | - | chr1_rcluster4182 | 61125697-61125789 | 6 |
| ENST00000412228.1 | chr1:1822911-1823290 | + | chr1_fcluster186 | 1823031-1823124 | 2 |
| ENST00000413987.1 | chr1:28835343-28837404 | + | chr1_fcluster2075 | 28835883-28835977 | 2 |
| ENST00000434112.1 | chr1:151512993-151513385 | + | chr1_fcluster8250 | 151513076-151513170 | 10 |
| ENST00000436484.1 | chr1:248884572-248885506 | - | chr1_rcluster13253 | 248884671-248884765 | 2 |
| ENST00000437681.1 | chr1:28835343-28836145 | + | chr1_fcluster2075 | 28835883-28835977 | 2 |
| ENST00000425496.2 | chr1:327553-328453 | + | chr1_fcluster21 | 327786-327881 | 2 |
| ENST00000432521.2 | chr1:2482432-2485589 | - | chr1_rcluster357 | 2485446-2485541 | 3 |
| ENST00000431268.1 | chr1:173833396-173834420 | - | chr1_rcluster9298 | 173833958-173834056 | 37869 |
| ENST00000442067.1 | chr1:173833770-173834685 | - | chr1_rcluster9298 | 173833958-173834056 | 37869 |
| ENST00000449386.1 | chr1:63787988-63788129 | - | chr1_rcluster4298 | 63788023-63788121 | 3 |
| ENST00000449853.1 | chr1:16931914-16934186 | - | chr1_rcluster1317 | 16932653-16932752 | 2 |
| ENST00000356006.3 | chr1:160918442-160919712 | + | chr1_fcluster8903 | 160918642-160918742 | 2 |
| ENST00000464612.1 | chr1:28907073-28907522 | - | chr1_rcluster2220 | 28907119-28907219 | 6 |
| ENST00000474814.1 | chr1:28907073-28908383 | - | chr1_rcluster2220 | 28907119-28907219 | 6 |
| ENST00000481368.1 | chr1:28906425-28907741 | - | chr1_rcluster2220 | 28907119-28907219 | 6 |
| ENST00000416301.1 | chr10:97512964-97516471 | - | chr10_rcluster3728 | 97515791-97515838 | 2 |
| ENST00000438010.1 | chr10:48962530-48962609 | + | chr10_fcluster1927 | 48962548-48962597 | 5 |
| ENST00000527986.1 | chr10:28811582-28813540 | - | chr10_rcluster1110 | 28811898-28811947 | 2 |
| ENST00000528337.1 | chr10:28811615-28813540 | - | chr10_rcluster1110 | 28811898-28811947 | 2 |
| ENST00000412789.1 | chr10:38265754-38265933 | + | chr10_fcluster1512 | 38265760-38265811 | 3 |
| ENST00000413603.1 | chr10:2055442-2055652 | - | chr10_rcluster110 | 2055444-2055497 | 2 |
| ENST00000421806.1 | chr10:98752208-98755716 | + | chr10_fcluster3986 | 98754953-98755006 | 3 |
| ENST00000423687.1 | chr10:38727787-38727850 | + | chr10_fcluster1541 | 38727797-38727850 | 7 |
| ENST00000431840.2 | chr10:47234379-47234518 | - | chr10_rcluster1853 | 47234449-47234502 | 3 |
| ENST00000438372.1 | chr10:2055442-2055699 | - | chr10_rcluster110 | 2055444-2055497 | 2 |
| ENST00000447412.2 | chr10:38727787-38727954 | + | chr10_fcluster1541 | 38727797-38727850 | 7 |
| ENST00000449800.1 | chr10:48943410-48943556 | - | chr10_rcluster1895 | 48943487-48943540 | 3 |
| ENST00000454424.1 | chr10:2055442-2055605 | - | chr10_rcluster110 | 2055444-2055497 | 2 |
| ENST00000415746.1 | chr10:72689683-72691210 | + | chr10_fcluster2771 | 72690496-72690551 | 3 |
| ENST00000418270.1 | chr10:8094399-8094600 | + | chr10_fcluster378 | 8094413-8094470 | 3 |
| ENST00000527595.1 | chr10:102265563-102265958 | - | chr10_rcluster4000 | 102265796-102265853 | 3 |
| ENST00000529568.1 | chr10:102265386-102265958 | - | chr10_rcluster4000 | 102265796-102265853 | 3 |
| ENST00000416301.1 | chr10:97849740-97849971 | - | chr10_rcluster3740 | 97849906-97849964 | 6 |
| ENST00000427846.1 | chr10:97849740-97849995 | - | chr10_rcluster3740 | 97849906-97849964 | 6 |
| ENST00000454638.1 | chr10:97849740-97849969 | - | chr10_rcluster3740 | 97849906-97849964 | 6 |
| ENST00000458228.1 | chr10:97849740-97849982 | - | chr10_rcluster3740 | 97849906-97849964 | 6 |
| ENST00000456514.1 | chr10:128110357-128110448 | - | chr10_rcluster5130 | 128110382-128110441 | 5 |
| ENST00000512032.1 | chr10:15211105-15213418 | + | chr10_fcluster663 | 15211112-15211173 | 10 |
| ENST00000422848.1 | chr10:99160873-99161130 | + | chr10_fcluster4001 | 99160983-99161045 | 2 |
| ENST00000428940.1 | chr10:88281703-88282166 | + | chr10_fcluster3470 | 88281759-88281825 | 2 |
| ENST00000478086.1 | chr10:97893314-97893405 | + | chr10_fcluster3935 | 97893317-97893384 | 2 |
| ENST00000534948.1 | chr10:97893314-97893405 | + | chr10_fcluster3935 | 97893317-97893384 | 2 |
| ENST00000449882.1 | chr10:91460858-91461177 | - | chr10_rcluster3454 | 91460878-91460949 | 2 |
| ENST00000454935.1 | chr10:102133373-102133769 | + | chr10_fcluster4159 | 102133599-102133670 | 2 |
| ENST00000429809.1 | chr10:122938215-122938486 | + | chr10_fcluster5051 | 122938282-122938356 | 2 |
| ENST00000431300.2 | chr10:81586460-81587113 | + | chr10_fcluster3247 | 81586626-81586701 | 5 |
| ENST00000499111.1 | chr10:18941166-18942371 | + | chr10_fcluster804 | 18941929-18942004 | 5 |
| ENST00000398701.2 | chr10:75571233-75571556 | - | chr10_rcluster2853 | 75571301-75571377 | 4 |
| ENST00000500803.2 | chr10:72427260-72428508 | + | chr10_fcluster2748 | 72427800-72427876 | 5 |
| ENST00000443282.1 | chr10:14695834-14695965 | + | chr10_fcluster634 | 14695863-14695941 | 2 |
| ENST00000535221.1 | chr10:7533657-7534658 | + | chr10_fcluster346 | 7533929-7534011 | 2 |
| ENST00000450054.1 | chr10:106376416-106376731 | + | chr10_fcluster4454 | 106376536-106376619 | 3 |
| ENST00000433920.1 | chr10:89102000-89102369 | - | chr10_rcluster3368 | 89102031-89102116 | 5 |
| ENST00000446751.1 | chr10:89102000-89102369 | - | chr10_rcluster3368 | 89102031-89102116 | 5 |
| ENST00000451940.1 | chr10:89102000-89102299 | - | chr10_rcluster3368 | 89102031-89102116 | 5 |
| ENST00000456938.1 | chr10:45497680-45499523 | - | chr10_rcluster1774 | 45497748-45497833 | 2 |
| ENST00000458739.1 | chr10:89102000-89102242 | - | chr10_rcluster3368 | 89102031-89102116 | 5 |
| ENST00000417112.1 | chr10:6780320-6780988 | + | chr10_fcluster327 | 6780398-6780484 | 3 |
| ENST00000456526.1 | chr10:9012089-9012235 | + | chr10_fcluster415 | 9012102-9012189 | 2 |
| ENST00000430651.1 | chr10:102997697-102998605 | + | chr10_fcluster4221 | 102998249-102998340 | 2 |
| ENST00000441365.2 | chr10:134598067-134598269 | - | chr10_rcluster5368 | 134598077-134598169 | 3 |
| ENST00000371192.1 | chr10:97915101-97915804 | + | chr10_fcluster3938 | 97915654-97915748 | 3 |
| ENST00000437930.1 | chr10:90699153-90699731 | + | chr10_fcluster3596 | 90699190-90699285 | 2 |
| ENST00000447412.2 | chr10:38711697-38711926 | + | chr10_fcluster1534 | 38711831-38711926 | 5 |
| ENST00000540599.1 | chr10:90699153-90699731 | + | chr10_fcluster3596 | 90699190-90699285 | 2 |
| ENST00000430295.1 | chr10:29698332-29698587 | + | chr10_fcluster1218 | 29698462-29698559 | 5 |
| ENST00000433920.1 | chr10:89102000-89102369 | - | chr10_rcluster3369 | 89102199-89102296 | 10 |
| ENST00000446751.1 | chr10:89102000-89102369 | - | chr10_rcluster3369 | 89102199-89102296 | 10 |
| ENST00000451940.1 | chr10:89102000-89102299 | - | chr10_rcluster3369 | 89102199-89102296 | 10 |
| ENST00000456967.1 | chr10:51826287-51827563 | - | chr10_rcluster2005 | 51827260-51827357 | 3 |
| ENST00000419697.1 | chr10:81563814-81564283 | - | chr10_rcluster3080 | 81564174-81564273 | 79 |
| ENST00000430963.1 | chr10:81563814-81564283 | - | chr10_rcluster3080 | 81564174-81564273 | 79 |
| ENST00000514385.1 | chr11:117688456-117690134 | - | chr11_rcluster4648 | 117689887-117689932 | 2 |
| ENST00000530595.1 | chr11:57417772-57420263 | + | chr11_fcluster2084 | 57418882-57418927 | 2 |
| ENST00000527970.1 | chr11:3602158-3602441 | - | chr11_rcluster309 | 3602210-3602256 | 2 |
| ENST00000530595.1 | chr11:57405850-57406145 | + | chr11_fcluster2076 | 57405855-57405901 | 4 |
| ENST00000535076.1 | chr11:62619521-62620153 | - | chr11_rcluster2483 | 62619731-62619778 | 3 |
| ENST00000537869.1 | chr11:62619464-62620153 | - | chr11_rcluster2483 | 62619731-62619778 | 3 |
| ENST00000537925.1 | chr11:62619461-62620153 | - | chr11_rcluster2483 | 62619731-62619778 | 3 |
| ENST00000538654.1 | chr11:62619462-62620153 | - | chr11_rcluster2483 | 62619731-62619778 | 3 |
| ENST00000539921.1 | chr11:62619461-62620153 | - | chr11_rcluster2483 | 62619731-62619778 | 3 |
| ENST00000539975.1 | chr11:62619493-62620153 | - | chr11_rcluster2483 | 62619731-62619778 | 3 |
| ENST00000540725.1 | chr11:62619461-62620153 | - | chr11_rcluster2483 | 62619731-62619778 | 3 |
| ENST00000542112.1 | chr11:62619731-62620153 | - | chr11_rcluster2483 | 62619731-62619778 | 3 |
| ENST00000545440.1 | chr11:62619725-62620153 | - | chr11_rcluster2483 | 62619731-62619778 | 3 |
| ENST00000501122.2 | chr11:65190270-65213011 | + | chr11_fcluster2651 | 65196852-65196900 | 2 |
| ENST00000501122.2 | chr11:65190270-65213011 | + | chr11_fcluster2667 | 65203788-65203836 | 3 |
| ENST00000501122.2 | chr11:65190270-65213011 | + | chr11_fcluster2681 | 65210534-65210582 | 3 |
| ENST00000532327.1 | chr11:133909658-133911143 | + | chr11_fcluster5629 | 133910809-133910857 | 2 |
| ENST00000533922.1 | chr11:133909658-133911143 | + | chr11_fcluster5629 | 133910809-133910857 | 2 |
| ENST00000525757.1 | chr11:123329081-123331118 | + | chr11_fcluster5264 | 123330839-123330888 | 2 |
| ENST00000532090.1 | chr11:65543365-65547496 | - | chr11_rcluster2742 | 65543479-65543528 | 1 |
| ENST00000546324.1 | chr11:73018928-73019609 | - | chr11_rcluster3239 | 73019124-73019173 | 2 |
| ENST00000502071.2 | chr11:74022871-74022991 | + | chr11_fcluster3402 | 74022873-74022923 | 2 |
| ENST00000534336.1 | chr11:65265234-65273940 | + | chr11_fcluster2706 | 65273801-65273851 | 6 |
| ENST00000007633.8 | chr11:67795381-67796741 | + | chr11_fcluster3006 | 67796498-67796549 | 3 |
| ENST00000342456.6 | chr11:67795381-67796741 | + | chr11_fcluster3006 | 67796498-67796549 | 3 |
| ENST00000434449.1 | chr11:67795218-67796743 | + | chr11_fcluster3006 | 67796498-67796549 | 3 |
| ENST00000529232.1 | chr11:67795218-67796744 | + | chr11_fcluster3006 | 67796498-67796549 | 3 |
| ENST00000531962.1 | chr11:32058203-32058542 | - | chr11_rcluster1343 | 32058280-32058331 | 2 |
| ENST00000534084.1 | chr11:61111609-61111745 | + | chr11_fcluster2255 | 61111616-61111667 | 2 |
| ENST00000539229.1 | chr11:67795381-67796741 | + | chr11_fcluster3006 | 67796498-67796549 | 3 |
| ENST00000501122.2 | chr11:65190270-65213011 | + | chr11_fcluster2647 | 65195490-65195543 | 3 |
| ENST00000501122.2 | chr11:65190270-65213011 | + | chr11_fcluster2677 | 65208220-65208273 | 4 |
| ENST00000501122.2 | chr11:65190270-65213011 | + | chr11_fcluster2684 | 65211349-65211403 | 3 |
| ENST00000527083.1 | chr11:27655879-27656069 | - | chr11_rcluster1233 | 27655992-27656046 | 7 |
| ENST00000533552.1 | chr11:59333239-59333547 | - | chr11_rcluster2145 | 59333478-59333532 | 2 |
| ENST00000534757.1 | chr11:27655879-27656174 | - | chr11_rcluster1233 | 27655992-27656046 | 7 |
| ENST00000527799.1 | chr11:665911-666490 | + | chr11_fcluster87 | 666396-666451 | 2 |
| ENST00000546324.1 | chr11:73019718-73020406 | - | chr11_rcluster3240 | 73020226-73020281 | 2 |
| ENST00000524555.1 | chr11:18257384-18257477 | - | chr11_rcluster978 | 18257398-18257454 | 2 |
| ENST00000532085.1 | chr11:118895902-118897805 | - | chr11_rcluster4735 | 118896414-118896470 | 2 |
| ENST00000411754.1 | chr11:2017749-2018505 | - | chr11_rcluster204 | 2017995-2018052 | 31 |
| ENST00000411861.1 | chr11:2017749-2018447 | - | chr11_rcluster204 | 2017995-2018052 | 31 |
| ENST00000412788.1 | chr11:2017749-2019044 | - | chr11_rcluster204 | 2017995-2018052 | 31 |
| ENST00000414790.1 | chr11:2017749-2019105 | - | chr11_rcluster204 | 2017995-2018052 | 31 |
| ENST00000417089.1 | chr11:2017745-2018255 | - | chr11_rcluster204 | 2017995-2018052 | 31 |
| ENST00000422826.1 | chr11:2017974-2019027 | - | chr11_rcluster204 | 2017995-2018052 | 31 |
| ENST00000431095.1 | chr11:2017749-2018180 | - | chr11_rcluster204 | 2017995-2018052 | 31 |
| ENST00000439725.1 | chr11:2017749-2018690 | - | chr11_rcluster204 | 2017995-2018052 | 31 |
| ENST00000446406.1 | chr11:2017749-2018056 | - | chr11_rcluster204 | 2017995-2018052 | 31 |
| ENST00000535745.1 | chr11:2017749-2018705 | - | chr11_rcluster204 | 2017995-2018052 | 31 |
| ENST00000557213.1 | chr11:2696557-2721224 | - | chr11_rcluster250 | 2709450-2709507 | 3 |
| ENST00000540312.1 | chr11:111383744-111384610 | + | chr11_fcluster4680 | 111384173-111384231 | 5872 |
| ENST00000411754.1 | chr11:2017749-2018505 | - | chr11_rcluster205 | 2018197-2018256 | 2 |
| ENST00000411861.1 | chr11:2017749-2018447 | - | chr11_rcluster205 | 2018197-2018256 | 2 |
| ENST00000412788.1 | chr11:2017749-2019044 | - | chr11_rcluster205 | 2018197-2018256 | 2 |
| ENST00000414790.1 | chr11:2017749-2019105 | - | chr11_rcluster205 | 2018197-2018256 | 2 |
| ENST00000422826.1 | chr11:2017974-2019027 | - | chr11_rcluster205 | 2018197-2018256 | 2 |
| ENST00000439725.1 | chr11:2017749-2018690 | - | chr11_rcluster205 | 2018197-2018256 | 2 |
| ENST00000501122.2 | chr11:65190270-65213011 | + | chr11_fcluster2648 | 65195652-65195711 | 4 |
| ENST00000525987.1 | chr11:111959592-111959735 | + | chr11_fcluster4716 | 111959613-111959672 | 4 |
| ENST00000531744.1 | chr11:111959592-111959735 | + | chr11_fcluster4716 | 111959613-111959672 | 4 |
| ENST00000532699.1 | chr11:111959592-111959735 | + | chr11_fcluster4716 | 111959613-111959672 | 4 |
| ENST00000535745.1 | chr11:2017749-2018705 | - | chr11_rcluster205 | 2018197-2018256 | 2 |
| ENST00000536683.1 | chr11:94964290-94964737 | + | chr11_fcluster4161 | 94964321-94964380 | 3 |
| ENST00000543150.1 | chr11:94964290-94965340 | + | chr11_fcluster4161 | 94964321-94964380 | 3 |
| ENST00000555531.1 | chr11:14539427-14539527 | - | chr11_rcluster826 | 14539436-14539495 | 2 |
| ENST00000539086.1 | chr11:64072016-64072241 | + | chr11_fcluster2527 | 64072042-64072102 | 8 |
| ENST00000499964.1 | chr11:67776912-67782038 | - | chr11_rcluster2938 | 67777943-67778004 | 4 |
| ENST00000530842.1 | chr11:67897266-67897549 | + | chr11_fcluster3023 | 67897444-67897505 | 5 |
| ENST00000543150.1 | chr11:94964290-94965340 | + | chr11_fcluster4162 | 94964865-94964927 | 11 |
| ENST00000433688.1 | chr11:63531511-63532070 | - | chr11_rcluster2543 | 63531792-63531855 | 20 |
| ENST00000445014.2 | chr11:63531617-63532070 | - | chr11_rcluster2543 | 63531792-63531855 | 20 |
| ENST00000524880.1 | chr11:111708973-111709101 | - | chr11_rcluster4416 | 111708973-111709037 | 2 |
| ENST00000502049.2 | chr11:46867979-46868050 | + | chr11_fcluster1845 | 46867984-46868049 | 4 |
| ENST00000531719.1 | chr11:46867964-46868050 | + | chr11_fcluster1845 | 46867984-46868049 | 4 |
| ENST00000501079.1 | chr11:10899929-10900823 | + | chr11_fcluster652 | 10900223-10900289 | 4 |
| ENST00000531518.1 | chr11:65713128-65713337 | + | chr11_fcluster2788 | 65713137-65713203 | 2 |
| ENST00000532620.1 | chr11:65712917-65713293 | + | chr11_fcluster2788 | 65713137-65713203 | 2 |
| ENST00000533763.1 | chr11:65713116-65713293 | + | chr11_fcluster2788 | 65713137-65713203 | 2 |
| ENST00000533859.1 | chr11:133229925-133232087 | - | chr11_rcluster5261 | 133231452-133231518 | 14 |
| ENST00000534740.1 | chr11:65713116-65713293 | + | chr11_fcluster2788 | 65713137-65713203 | 2 |
| ENST00000538111.1 | chr11:72477013-72477338 | + | chr11_fcluster3316 | 72477094-72477160 | 171 |
| ENST00000389919.4 | chr11:58346646-58347095 | + | chr11_fcluster2152 | 58346697-58346765 | 3 |
| ENST00000389919.4 | chr11:58384670-58385041 | + | chr11_fcluster2154 | 58384789-58384858 | 2 |
| ENST00000422974.1 | chr11:58384670-58385041 | + | chr11_fcluster2154 | 58384789-58384858 | 2 |
| ENST00000545079.1 | chr11:67612674-67615460 | - | chr11_rcluster2926 | 67613905-67613974 | 2 |
| ENST00000389919.4 | chr11:58391508-58392039 | + | chr11_fcluster2159 | 58391745-58391815 | 3 |
| ENST00000422974.1 | chr11:58391508-58392110 | + | chr11_fcluster2159 | 58391745-58391815 | 3 |
| ENST00000433688.1 | chr11:63527365-63531341 | - | chr11_rcluster2541 | 63530838-63530909 | 3 |
| ENST00000530621.1 | chr11:133667936-133670210 | - | chr11_rcluster5279 | 133669586-133669657 | 2 |
| ENST00000496634.2 | chr11:62339013-62339154 | - | chr11_rcluster2399 | 62339077-62339149 | 10 |
| ENST00000525548.1 | chr11:107992024-107992411 | - | chr11_rcluster4284 | 107992061-107992133 | 5 |
| ENST00000526409.1 | chr11:62339013-62339154 | - | chr11_rcluster2399 | 62339077-62339149 | 10 |
| ENST00000530583.1 | chr11:129872834-129874251 | + | chr11_fcluster5508 | 129872867-129872941 | 4 |
| ENST00000433688.1 | chr11:63527365-63531341 | - | chr11_rcluster2540 | 63527443-63527519 | 3 |
| ENST00000499027.1 | chr11:45234531-45236289 | - | chr11_rcluster1678 | 45235875-45235951 | 2 |
| ENST00000500185.2 | chr11:70244205-70244594 | - | chr11_rcluster3080 | 70244441-70244518 | 5 |
| ENST00000534120.1 | chr11:59436786-59437015 | + | chr11_fcluster2201 | 59436809-59436889 | 4 |
| ENST00000535307.1 | chr11:61564395-61564704 | + | chr11_fcluster2289 | 61564567-61564648 | 3 |
| ENST00000500185.2 | chr11:70219517-70221726 | - | chr11_rcluster3078 | 70221425-70221507 | 2 |
| ENST00000501122.2 | chr11:65190270-65213011 | + | chr11_fcluster2671 | 65205061-65205143 | 7 |
| ENST00000502284.1 | chr11:71725338-71727933 | + | chr11_fcluster3266 | 71725499-71725581 | 2 |
| ENST00000399269.2 | chr11:45168195-45168302 | + | chr11_fcluster1757 | 45168205-45168288 | 2 |
| ENST00000501122.2 | chr11:65190270-65213011 | + | chr11_fcluster2680 | 65209453-65209536 | 4 |
| ENST00000530583.1 | chr11:129872834-129874251 | + | chr11_fcluster5509 | 129873595-129873678 | 9 |
| ENST00000531858.1 | chr11:22524206-22524392 | + | chr11_fcluster1078 | 22524266-22524349 | 4 |
| ENST00000534904.1 | chr11:114231358-114234889 | - | chr11_rcluster4516 | 114231458-114231541 | 2 |
| ENST00000541416.1 | chr11:62621553-62622023 | - | chr11_rcluster2487 | 62621648-62621731 | 2 |
| ENST00000545920.1 | chr11:62621274-62621814 | - | chr11_rcluster2487 | 62621648-62621731 | 2 |
| ENST00000530759.1 | chr11:76155323-76155618 | - | chr11_rcluster3389 | 76155324-76155408 | 3 |
| ENST00000458348.2 | chr11:63531313-63531504 | - | chr11_rcluster2542 | 63531387-63531472 | 6 |
| ENST00000501122.2 | chr11:65190270-65213011 | + | chr11_fcluster2678 | 65208386-65208472 | 2 |
| ENST00000403734.2 | chr11:62474582-62474796 | - | chr11_rcluster2444 | 62474665-62474753 | 3 |
| ENST00000499964.1 | chr11:67776912-67782038 | - | chr11_rcluster2937 | 67777516-67777604 | 10 |
| ENST00000532350.1 | chr11:122073473-122073770 | - | chr11_rcluster4873 | 122073556-122073644 | 2 |
| ENST00000534496.1 | chr11:122073473-122073754 | - | chr11_rcluster4873 | 122073556-122073644 | 2 |
| ENST00000534782.1 | chr11:122073473-122073770 | - | chr11_rcluster4873 | 122073556-122073644 | 2 |
| ENST00000524376.1 | chr11:121959819-121962427 | - | chr11_rcluster4865 | 121959982-121960071 | 2 |
| ENST00000528986.1 | chr11:121959819-121962448 | - | chr11_rcluster4865 | 121959982-121960071 | 2 |
| ENST00000529823.1 | chr11:121959815-121962427 | - | chr11_rcluster4865 | 121959982-121960071 | 2 |
| ENST00000531381.1 | chr11:121959812-121962448 | - | chr11_rcluster4865 | 121959982-121960071 | 2 |
| ENST00000499027.1 | chr11:45234531-45236289 | - | chr11_rcluster1677 | 45234733-45234824 | 3 |
| ENST00000524880.1 | chr11:111711379-111711532 | - | chr11_rcluster4417 | 111711397-111711490 | 4 |
| ENST00000543494.1 | chr11:67119044-67119490 | - | chr11_rcluster2874 | 67119071-67119164 | 2 |
| ENST00000540375.1 | chr11:127116-128016 | - | chr11_rcluster6 | 127686-127781 | 2 |
| ENST00000531263.1 | chr11:75525509-75525842 | - | chr11_rcluster3362 | 75525623-75525719 | 2 |
| ENST00000496634.2 | chr11:62340057-62342380 | - | chr11_rcluster2402 | 62340483-62340580 | 2 |
| ENST00000501122.2 | chr11:65190270-65213011 | + | chr11_fcluster2660 | 65200176-65200273 | 6 |
| ENST00000534336.1 | chr11:65265234-65273940 | + | chr11_fcluster2704 | 65265480-65265577 | 2 |
| ENST00000547559.1 | chr12:55407803-55408031 | + | chr12_fcluster4367 | 55407884-55407929 | 8 |
| ENST00000537269.1 | chr12:7073365-7073610 | + | chr12_fcluster715 | 7073501-7073547 | 5 |
| ENST00000544657.1 | chr12:7282310-7282848 | - | chr12_rcluster647 | 7282734-7282781 | 6 |
| ENST00000500632.1 | chr12:27124520-27125478 | + | chr12_fcluster2308 | 27125093-27125141 | 8 |
| ENST00000546227.1 | chr12:122237754-122238583 | - | chr12_rcluster9509 | 122238203-122238251 | 2 |
| ENST00000549914.1 | chr12:93397192-93397749 | + | chr12_fcluster7123 | 93397203-93397251 | 2 |
| ENST00000551032.1 | chr12:79935987-79936190 | + | chr12_fcluster6345 | 79936074-79936124 | 11 |
| ENST00000536397.1 | chr12:31742858-31743053 | + | chr12_fcluster2641 | 31742882-31742937 | 3 |
| ENST00000547712.1 | chr12:49524955-49525164 | + | chr12_fcluster3610 | 49525082-49525138 | 2 |
| ENST00000539450.1 | chr12:123463301-123466426 | + | chr12_fcluster9883 | 123466272-123466330 | 6 |
| ENST00000535844.1 | chr12:122748120-122748246 | - | chr12_rcluster9570 | 122748179-122748238 | 4 |
| ENST00000537157.1 | chr12:122238755-122238913 | - | chr12_rcluster9510 | 122238848-122238907 | 8 |
| ENST00000546793.1 | chr12:53436974-53439437 | - | chr12_rcluster3943 | 53438123-53438182 | 2 |
| ENST00000499137.2 | chr12:93936240-93939134 | - | chr12_rcluster7002 | 93936484-93936544 | 2 |
| ENST00000500986.1 | chr12:93936241-93937081 | - | chr12_rcluster7002 | 93936484-93936544 | 2 |
| ENST00000508564.1 | chr12:54525775-54526627 | + | chr12_fcluster4268 | 54526025-54526086 | 2 |
| ENST00000538559.2 | chr12:97856443-97856650 | + | chr12_fcluster7482 | 97856483-97856544 | 2 |
| ENST00000549023.1 | chr12:49627353-49627921 | - | chr12_rcluster3584 | 49627794-49627855 | 2 |
| ENST00000423999.2 | chr12:122502264-122502588 | + | chr12_fcluster9733 | 122502276-122502338 | 10 |
| ENST00000500527.1 | chr12:31173698-31174963 | - | chr12_rcluster2375 | 31174363-31174425 | 2 |
| ENST00000536864.1 | chr12:8549020-8549399 | - | chr12_rcluster817 | 8549325-8549387 | 2 |
| ENST00000540968.1 | chr12:122502046-122502339 | + | chr12_fcluster9733 | 122502276-122502338 | 10 |
| ENST00000544461.1 | chr12:8549020-8549399 | - | chr12_rcluster817 | 8549325-8549387 | 2 |
| ENST00000447687.2 | chr12:6556997-6557316 | - | chr12_rcluster505 | 6557126-6557190 | 19 |
| ENST00000504270.2 | chr12:6556783-6557316 | - | chr12_rcluster505 | 6557126-6557190 | 19 |
| ENST00000536388.1 | chr12:6556940-6557316 | - | chr12_rcluster505 | 6557126-6557190 | 19 |
| ENST00000514568.2 | chr12:8946902-8948385 | + | chr12_fcluster918 | 8947392-8947457 | 2 |
| ENST00000547717.1 | chr12:53858545-53858636 | + | chr12_fcluster4195 | 53858565-53858630 | 5 |
| ENST00000545339.1 | chr12:6560636-6560733 | - | chr12_rcluster507 | 6560637-6560703 | 2 |
| ENST00000538790.1 | chr12:122501213-122501437 | + | chr12_fcluster9731 | 122501317-122501385 | 2 |
| ENST00000540968.1 | chr12:122501256-122501437 | + | chr12_fcluster9731 | 122501317-122501385 | 2 |
| ENST00000515617.1 | chr12:54512389-54516018 | + | chr12_fcluster4266 | 54513995-54514064 | 2 |
| ENST00000550029.1 | chr12:104157091-104157596 | - | chr12_rcluster7790 | 104157456-104157525 | 3 |
| ENST00000553259.1 | chr12:49686415-49687679 | - | chr12_rcluster3591 | 49686704-49686773 | 4 |
| ENST00000547179.1 | chr12:103203059-103206394 | - | chr12_rcluster7729 | 103204574-103204644 | 3 |
| ENST00000547717.1 | chr12:53861006-53861312 | + | chr12_fcluster4198 | 53861008-53861078 | 13 |
| ENST00000547834.1 | chr12:2921729-2922068 | - | chr12_rcluster203 | 2921989-2922059 | 3 |
| ENST00000550380.1 | chr12:76687594-76687785 | + | chr12_fcluster6172 | 76687651-76687723 | 5 |
| ENST00000553247.1 | chr12:76687594-76687785 | + | chr12_fcluster6172 | 76687651-76687723 | 5 |
| ENST00000547395.1 | chr12:49412020-49412502 | + | chr12_fcluster3597 | 49412041-49412114 | 2 |
| ENST00000547866.1 | chr12:49411187-49412980 | + | chr12_fcluster3597 | 49412041-49412114 | 2 |
| ENST00000552284.1 | chr12:49412020-49412563 | + | chr12_fcluster3597 | 49412041-49412114 | 2 |
| ENST00000552933.1 | chr12:49412020-49412988 | + | chr12_fcluster3597 | 49412041-49412114 | 2 |
| ENST00000535614.1 | chr12:122237338-122237563 | - | chr12_rcluster9508 | 122237474-122237548 | 3 |
| ENST00000535643.1 | chr12:122237079-122237563 | - | chr12_rcluster9508 | 122237474-122237548 | 3 |
| ENST00000536662.1 | chr12:122237460-122237563 | - | chr12_rcluster9508 | 122237474-122237548 | 3 |
| ENST00000543334.1 | chr12:122237291-122237563 | - | chr12_rcluster9508 | 122237474-122237548 | 3 |
| ENST00000547717.1 | chr12:53853057-53853187 | + | chr12_fcluster4191 | 53853099-53853176 | 5 |
| ENST00000504738.2 | chr12:6437934-6438788 | - | chr12_rcluster478 | 6438095-6438173 | 2 |
| ENST00000547721.1 | chr12:76427045-76427677 | + | chr12_fcluster6152 | 76427113-76427191 | 2 |
| ENST00000500527.1 | chr12:31226130-31226781 | - | chr12_rcluster2383 | 31226608-31226687 | 6 |
| ENST00000548172.1 | chr12:90687101-90687337 | + | chr12_fcluster6965 | 90687258-90687337 | 4 |
| ENST00000549470.1 | chr12:90687096-90687337 | + | chr12_fcluster6965 | 90687258-90687337 | 4 |
| ENST00000539450.1 | chr12:123463301-123466426 | + | chr12_fcluster9882 | 123465227-123465308 | 4 |
| ENST00000541871.1 | chr12:2158301-2158629 | + | chr12_fcluster227 | 2158425-2158506 | 3 |
| ENST00000534843.1 | chr12:12982358-12982909 | + | chr12_fcluster1347 | 12982700-12982783 | 2 |
| ENST00000550042.1 | chr12:77718422-77718994 | + | chr12_fcluster6249 | 77718672-77718755 | 2 |
| ENST00000500632.1 | chr12:27130928-27132515 | + | chr12_fcluster2309 | 27131696-27131780 | 2 |
| ENST00000539450.1 | chr12:123463301-123466426 | + | chr12_fcluster9881 | 123464779-123464863 | 3 |
| ENST00000552576.1 | chr12:56122889-56123139 | + | chr12_fcluster4409 | 56122921-56123005 | 38 |
| ENST00000505661.1 | chr12:54519545-54519776 | - | chr12_rcluster4080 | 54519636-54519722 | 2 |
| ENST00000551894.1 | chr12:52599369-52599963 | - | chr12_rcluster3867 | 52599550-52599639 | 2 |
| ENST00000546821.1 | chr12:50315710-50317464 | + | chr12_fcluster3726 | 50317185-50317275 | 3 |
| ENST00000428207.1 | chr12:112279132-112280706 | - | chr12_rcluster8522 | 112279640-112279731 | 11 |
| ENST00000456429.1 | chr12:112279132-112280706 | - | chr12_rcluster8522 | 112279640-112279731 | 11 |
| ENST00000539450.1 | chr12:123463301-123466426 | + | chr12_fcluster9880 | 123464413-123464504 | 3 |
| ENST00000553202.1 | chr12:44112797-44117803 | - | chr12_rcluster3080 | 44115618-44115709 | 3 |
| ENST00000539009.1 | chr12:10490645-10490891 | - | chr12_rcluster1020 | 10490693-10490785 | 8 |
| ENST00000547717.1 | chr12:53848511-53848653 | + | chr12_fcluster4188 | 53848561-53848653 | 17 |
| ENST00000548359.1 | chr12:79933984-79934226 | + | chr12_fcluster6339 | 79934082-79934176 | 7 |
| ENST00000539163.1 | chr12:121407642-121410095 | - | chr12_rcluster9395 | 121408799-121408894 | 2 |
| ENST00000538062.1 | chr12:7282105-7282613 | - | chr12_rcluster646 | 7282502-7282598 | 2 |
| ENST00000544657.1 | chr12:7282310-7282848 | - | chr12_rcluster646 | 7282502-7282598 | 2 |
| ENST00000504738.2 | chr12:6437934-6438788 | - | chr12_rcluster479 | 6438365-6438462 | 3 |
| ENST00000552576.1 | chr12:56123447-56124467 | + | chr12_fcluster4411 | 56123603-56123701 | 4 |
| ENST00000500076.2 | chr12:30908009-30908205 | + | chr12_fcluster2563 | 30908032-30908131 | 7 |
| ENST00000504738.2 | chr12:6437934-6438788 | - | chr12_rcluster480 | 6438543-6438642 | 2 |
| ENST00000535844.1 | chr12:122692327-122693124 | - | chr12_rcluster9561 | 122692770-122692869 | 2 |
| ENST00000547021.1 | chr12:112037633-112038060 | + | chr12_fcluster8706 | 112037754-112037853 | 3 |
| ENST00000537616.1 | chr12:9812238-9814681 | + | chr12_fcluster1018 | 9812336-9812436 | 3 |
| ENST00000423869.1 | chr13:48575526-48575958 | + | chr13_fcluster1593 | 48575570-48575615 | 3 |
| ENST00000433569.1 | chr13:95353520-95355116 | - | chr13_rcluster3812 | 95353992-95354038 | 3 |
| ENST00000428783.1 | chr13:32526633-32527609 | + | chr13_fcluster804 | 32527323-32527370 | 2 |
| ENST00000490410.1 | chr13:32524297-32527578 | + | chr13_fcluster804 | 32527323-32527370 | 2 |
| ENST00000423442.1 | chr13:54701416-54701564 | - | chr13_rcluster2095 | 54701486-54701535 | 14 |
| ENST00000427299.1 | chr13:54701416-54701564 | - | chr13_rcluster2095 | 54701486-54701535 | 14 |
| ENST00000451744.1 | chr13:54700998-54701564 | - | chr13_rcluster2095 | 54701486-54701535 | 14 |
| ENST00000560584.1 | chr13:79189811-79193239 | + | chr13_fcluster2960 | 79192722-79192773 | 2 |
| ENST00000444442.1 | chr13:44120670-44122192 | + | chr13_fcluster1371 | 44120706-44120758 | 3 |
| ENST00000427299.1 | chr13:54706498-54706748 | - | chr13_rcluster2099 | 54706573-54706627 | 8 |
| ENST00000437721.1 | chr13:30727172-30733758 | - | chr13_rcluster745 | 30732580-30732634 | 2 |
| ENST00000440633.1 | chr13:31457232-31457532 | + | chr13_fcluster745 | 31457471-31457526 | 5 |
| ENST00000499499.2 | chr13:96328107-96329179 | - | chr13_rcluster3881 | 96328773-96328830 | 3 |
| ENST00000413646.2 | chr13:22615115-22615523 | + | chr13_fcluster239 | 22615434-22615492 | 2 |
| ENST00000417989.1 | chr13:53720675-53721037 | - | chr13_rcluster2060 | 53720890-53720951 | 3 |
| ENST00000442500.1 | chr13:114581674-114582305 | - | chr13_rcluster4788 | 114581797-114581859 | 2 |
| ENST00000434849.1 | chr13:45949070-45951350 | + | chr13_fcluster1491 | 45950216-45950279 | 2 |
| ENST00000424106.1 | chr13:50351805-50354609 | - | chr13_rcluster1837 | 50352762-50352836 | 3 |
| ENST00000500111.1 | chr13:21872481-21875028 | - | chr13_rcluster221 | 21872823-21872897 | 4 |
| ENST00000428783.1 | chr13:32526633-32527609 | + | chr13_fcluster805 | 32527526-32527603 | 3 |
| ENST00000441924.2 | chr13:100139217-100140070 | - | chr13_rcluster4080 | 100139650-100139728 | 2 |
| ENST00000420219.1 | chr13:30950965-30951282 | - | chr13_rcluster755 | 30951039-30951120 | 3 |
| ENST00000499499.2 | chr13:96328107-96329179 | - | chr13_rcluster3880 | 96328444-96328526 | 2 |
| ENST00000330825.3 | chr13:45961721-45963955 | + | chr13_fcluster1497 | 45962759-45962842 | 2 |
| ENST00000330825.3 | chr13:45965168-45965610 | + | chr13_fcluster1498 | 45965395-45965478 | 2 |
| ENST00000379050.2 | chr13:45965168-45965683 | + | chr13_fcluster1498 | 45965395-45965478 | 2 |
| ENST00000517509.1 | chr13:45965168-45965618 | + | chr13_fcluster1498 | 45965395-45965478 | 2 |
| ENST00000520432.1 | chr13:45965168-45965487 | + | chr13_fcluster1498 | 45965395-45965478 | 2 |
| ENST00000522673.1 | chr13:45965168-45965857 | + | chr13_fcluster1498 | 45965395-45965478 | 2 |
| ENST00000524062.1 | chr13:45965168-45965872 | + | chr13_fcluster1498 | 45965395-45965478 | 2 |
| ENST00000344312.4 | chr13:33859608-33859873 | - | chr13_rcluster918 | 33859778-33859863 | 6 |
| ENST00000413591.1 | chr13:30677316-30679832 | - | chr13_rcluster740 | 30679200-30679285 | 2 |
| ENST00000422609.1 | chr13:19246968-19247248 | + | chr13_fcluster22 | 19247151-19247237 | 2 |
| ENST00000411690.1 | chr13:110707121-110707299 | + | chr13_fcluster4430 | 110707144-110707232 | 2 |
| ENST00000454605.1 | chr13:51484620-51484848 | - | chr13_rcluster1917 | 51484656-51484745 | 2 |
| ENST00000423869.1 | chr13:48575526-48575958 | + | chr13_fcluster1594 | 48575712-48575806 | 2 |
| ENST00000437721.1 | chr13:30727172-30733758 | - | chr13_rcluster744 | 30727699-30727797 | 3 |
| ENST00000555918.1 | chr14:36294030-36295971 | - | chr14_rcluster1086 | 36295893-36295939 | 7 |
| ENST00000418499.2 | chr14:19655759-19655871 | + | chr14_fcluster61 | 19655823-19655871 | 8 |
| ENST00000441500.1 | chr14:19655735-19655871 | + | chr14_fcluster61 | 19655823-19655871 | 8 |
| ENST00000546389.1 | chr14:19655783-19655871 | + | chr14_fcluster61 | 19655823-19655871 | 8 |
| ENST00000547220.1 | chr14:19655735-19655871 | + | chr14_fcluster61 | 19655823-19655871 | 8 |
| ENST00000549484.1 | chr14:19655735-19655871 | + | chr14_fcluster61 | 19655823-19655871 | 8 |
| ENST00000551881.2 | chr14:19655735-19655871 | + | chr14_fcluster61 | 19655823-19655871 | 8 |
| ENST00000553119.1 | chr14:19655735-19655871 | + | chr14_fcluster61 | 19655823-19655871 | 8 |
| ENST00000455232.1 | chr14:76044121-76045931 | - | chr14_rcluster3274 | 76044773-76044823 | 2 |
| ENST00000508827.1 | chr14:62027462-62031959 | - | chr14_rcluster2358 | 62029343-62029394 | 2 |
| ENST00000548109.1 | chr14:19859458-19859970 | + | chr14_fcluster87 | 19859855-19859906 | 2 |
| ENST00000557409.1 | chr14:69658614-69658809 | - | chr14_rcluster2825 | 69658639-69658690 | 2 |
| ENST00000547644.2 | chr14:96178085-96178235 | + | chr14_fcluster4147 | 96178140-96178192 | 2 |
| ENST00000553445.1 | chr14:96178095-96178362 | + | chr14_fcluster4147 | 96178140-96178192 | 2 |
| ENST00000553946.1 | chr14:61346433-61346553 | + | chr14_fcluster2141 | 61346496-61346548 | 2 |
| ENST00000558575.1 | chr14:75762824-75763341 | + | chr14_fcluster3136 | 75763264-75763316 | 1 |
| ENST00000553657.1 | chr14:58740733-58740853 | - | chr14_rcluster2177 | 58740788-58740843 | 26 |
| ENST00000555198.1 | chr14:70696736-70696898 | + | chr14_fcluster2737 | 70696830-70696885 | 2 |
| ENST00000555275.1 | chr14:58740698-58740853 | - | chr14_rcluster2177 | 58740788-58740843 | 26 |
| ENST00000555480.1 | chr14:70696736-70696904 | + | chr14_fcluster2737 | 70696830-70696885 | 2 |
| ENST00000555707.1 | chr14:58739120-58740853 | - | chr14_rcluster2177 | 58740788-58740843 | 26 |
| ENST00000556964.1 | chr14:70935599-70938309 | - | chr14_rcluster2913 | 70937993-70938048 | 2 |
| ENST00000436530.1 | chr14:54080372-54080744 | - | chr14_rcluster1908 | 54080642-54080699 | 2 |
| ENST00000555771.1 | chr14:71788392-71788531 | - | chr14_rcluster2964 | 71788444-71788501 | 2 |
| ENST00000557412.1 | chr14:58753359-58753741 | - | chr14_rcluster2181 | 58753385-58753442 | 2 |
| ENST00000559615.1 | chr14:24403387-24403777 | - | chr14_rcluster494 | 24403528-24403585 | 2 |
| ENST00000508827.1 | chr14:62027462-62031959 | - | chr14_rcluster2359 | 62029579-62029638 | 4 |
| ENST00000553860.1 | chr14:100715579-100715854 | - | chr14_rcluster4466 | 100715697-100715756 | 2 |
| ENST00000508827.1 | chr14:62027462-62031959 | - | chr14_rcluster2360 | 62031314-62031375 | 2 |
| ENST00000555379.1 | chr14:21668239-21673662 | - | chr14_rcluster234 | 21668439-21668501 | 3 |
| ENST00000465991.1 | chr14:24683517-24683601 | - | chr14_rcluster537 | 24683531-24683594 | 3 |
| ENST00000524914.1 | chr14:67666245-67667277 | + | chr14_fcluster2518 | 67667205-67667268 | 8 |
| ENST00000551565.1 | chr14:19505872-19506169 | + | chr14_fcluster33 | 19505874-19505937 | 14 |
| ENST00000553596.1 | chr14:32544626-32545999 | - | chr14_rcluster861 | 32545610-32545673 | 7 |
| ENST00000554441.1 | chr14:102024971-102025171 | - | chr14_rcluster4543 | 102025054-102025117 | 2 |
| ENST00000554694.1 | chr14:102024971-102025171 | - | chr14_rcluster4543 | 102025054-102025117 | 2 |
| ENST00000554735.1 | chr14:102023642-102026748 | - | chr14_rcluster4543 | 102025054-102025117 | 2 |
| ENST00000555174.1 | chr14:102023642-102025171 | - | chr14_rcluster4543 | 102025054-102025117 | 2 |
| ENST00000555882.1 | chr14:102024891-102025171 | - | chr14_rcluster4543 | 102025054-102025117 | 2 |
| ENST00000556328.1 | chr14:89017942-89018163 | + | chr14_fcluster3739 | 89018027-89018090 | 2 |
| ENST00000557532.1 | chr14:102024971-102025171 | - | chr14_rcluster4543 | 102025054-102025117 | 2 |
| ENST00000557661.1 | chr14:102023642-102025171 | - | chr14_rcluster4543 | 102025054-102025117 | 2 |
| ENST00000553932.1 | chr14:39304747-39308408 | - | chr14_rcluster1205 | 39305542-39305606 | 2 |
| ENST00000554732.1 | chr14:39304747-39308408 | - | chr14_rcluster1205 | 39305542-39305606 | 2 |
| ENST00000557440.1 | chr14:39304747-39307662 | - | chr14_rcluster1205 | 39305542-39305606 | 2 |
| ENST00000503525.2 | chr14:96389119-96391908 | + | chr14_fcluster4155 | 96390958-96391024 | 4 |
| ENST00000504119.1 | chr14:96389119-96391899 | + | chr14_fcluster4155 | 96390958-96391024 | 4 |
| ENST00000557565.1 | chr14:35778121-35778201 | + | chr14_fcluster915 | 35778135-35778201 | 5 |
| ENST00000554360.1 | chr14:58732084-58734111 | - | chr14_rcluster2175 | 58732620-58732688 | 2 |
| ENST00000555294.1 | chr14:23452108-23452290 | + | chr14_fcluster321 | 23452121-23452189 | 8 |
| ENST00000556503.1 | chr14:23451908-23452290 | + | chr14_fcluster321 | 23452121-23452189 | 8 |
| ENST00000557615.1 | chr14:23451908-23452290 | + | chr14_fcluster321 | 23452121-23452189 | 8 |
| ENST00000554235.1 | chr14:53620073-53620449 | + | chr14_fcluster1706 | 53620175-53620244 | 2 |
| ENST00000557565.1 | chr14:35592159-35593437 | + | chr14_fcluster901 | 35592167-35592236 | 3 |
| ENST00000554169.1 | chr14:96000031-96000582 | - | chr14_rcluster4249 | 96000142-96000212 | 34 |
| ENST00000555379.1 | chr14:21668239-21673662 | - | chr14_rcluster236 | 21670311-21670382 | 2 |
| ENST00000418499.2 | chr14:19683693-19684834 | + | chr14_fcluster70 | 19684236-19684310 | 3 |
| ENST00000500370.2 | chr14:96000373-96001137 | - | chr14_rcluster4250 | 96000874-96000948 | 6 |
| ENST00000398474.3 | chr14:101298850-101298978 | + | chr14_fcluster4439 | 101298872-101298947 | 3 |
| ENST00000398518.2 | chr14:101298850-101298978 | + | chr14_fcluster4439 | 101298872-101298947 | 3 |
| ENST00000423456.1 | chr14:101298850-101298978 | + | chr14_fcluster4439 | 101298872-101298947 | 3 |
| ENST00000429159.2 | chr14:101298850-101298978 | + | chr14_fcluster4439 | 101298872-101298947 | 3 |
| ENST00000431094.2 | chr14:19691356-19695180 | + | chr14_fcluster73 | 19691646-19691721 | 3 |
| ENST00000432001.1 | chr14:19691356-19691922 | + | chr14_fcluster73 | 19691646-19691721 | 3 |
| ENST00000441500.1 | chr14:19691356-19692921 | + | chr14_fcluster73 | 19691646-19691721 | 3 |
| ENST00000452120.2 | chr14:101295372-101299665 | + | chr14_fcluster4439 | 101298872-101298947 | 3 |
| ENST00000455088.2 | chr14:19882465-19884029 | - | chr14_rcluster74 | 19883662-19883737 | 3 |
| ENST00000521812.1 | chr14:101298402-101298978 | + | chr14_fcluster4439 | 101298872-101298947 | 3 |
| ENST00000549813.1 | chr14:19883544-19884029 | - | chr14_rcluster74 | 19883662-19883737 | 3 |
| ENST00000552602.1 | chr14:19880060-19884029 | - | chr14_rcluster74 | 19883662-19883737 | 3 |
| ENST00000556736.1 | chr14:101298850-101298978 | + | chr14_fcluster4439 | 101298872-101298947 | 3 |
| ENST00000547779.1 | chr14:19662180-19662608 | + | chr14_fcluster64 | 19662249-19662325 | 5 |
| ENST00000548057.2 | chr14:19912016-19913438 | - | chr14_rcluster84 | 19913037-19913113 | 5 |
| ENST00000548107.1 | chr14:19912756-19913184 | - | chr14_rcluster84 | 19913037-19913113 | 5 |
| ENST00000551881.2 | chr14:19661926-19663469 | + | chr14_fcluster64 | 19662249-19662325 | 5 |
| ENST00000555045.1 | chr14:24408823-24410367 | - | chr14_rcluster495 | 24408911-24408990 | 2 |
| ENST00000556182.1 | chr14:69658010-69658137 | - | chr14_rcluster2824 | 69658049-69658128 | 3 |
| ENST00000556379.1 | chr14:24407941-24410367 | - | chr14_rcluster495 | 24408911-24408990 | 2 |
| ENST00000557409.1 | chr14:69658010-69658183 | - | chr14_rcluster2824 | 69658049-69658128 | 3 |
| ENST00000529171.1 | chr14:60706846-60707172 | - | chr14_rcluster2290 | 60706936-60707016 | 2 |
| ENST00000532515.1 | chr14:60706840-60709588 | - | chr14_rcluster2290 | 60706936-60707016 | 2 |
| ENST00000553269.1 | chr14:60706851-60707172 | - | chr14_rcluster2290 | 60706936-60707016 | 2 |
| ENST00000553775.1 | chr14:60706861-60707172 | - | chr14_rcluster2290 | 60706936-60707016 | 2 |
| ENST00000531973.1 | chr14:73957670-73960096 | + | chr14_fcluster2946 | 73957684-73957765 | 12 |
| ENST00000524914.1 | chr14:67666245-67667277 | + | chr14_fcluster2517 | 67666342-67666427 | 2 |
| ENST00000452053.1 | chr14:106437783-106438358 | - | chr14_rcluster4965 | 106438150-106438236 | 2 |
| ENST00000419459.1 | chr14:90924941-90925248 | + | chr14_fcluster3864 | 90925113-90925201 | 2 |
| ENST00000442515.1 | chr14:90924941-90925210 | + | chr14_fcluster3864 | 90925113-90925201 | 2 |
| ENST00000444942.1 | chr14:90924941-90925249 | + | chr14_fcluster3864 | 90925113-90925201 | 2 |
| ENST00000551334.1 | chr14:19854099-19857070 | - | chr14_rcluster69 | 19856838-19856927 | 5 |
| ENST00000553119.1 | chr14:19718327-19718563 | + | chr14_fcluster79 | 19718468-19718557 | 5 |
| ENST00000558468.1 | chr14:24633824-24634164 | + | chr14_fcluster431 | 24633853-24633943 | 2 |
| ENST00000557467.1 | chr14:57359827-57360428 | - | chr14_rcluster2102 | 57360294-57360385 | 3 |
| ENST00000554568.1 | chr14:21511516-21514097 | - | chr14_rcluster217 | 21512245-21512337 | 67 |
| ENST00000556602.1 | chr14:57822165-57826128 | + | chr14_fcluster1946 | 57825874-57825966 | 12 |
| ENST00000556919.1 | chr14:69152089-69152282 | - | chr14_rcluster2740 | 69152089-69152181 | 5 |
| ENST00000558468.1 | chr14:24629730-24630547 | + | chr14_fcluster429 | 24630454-24630547 | 3 |
| ENST00000560931.1 | chr14:103399912-103400502 | + | chr14_fcluster4703 | 103400406-103400502 | 2 |
| ENST00000555937.1 | chr14:62200857-62201003 | + | chr14_fcluster2205 | 62200860-62200959 | 7 |
| ENST00000556072.1 | chr14:77252429-77253067 | - | chr14_rcluster3319 | 77252610-77252709 | 2 |
| ENST00000534909.2 | chr14:57364259-57364366 | + | chr14_fcluster1933 | 57364262-57364362 | 2 |
| ENST00000555655.1 | chr14:38671988-38672128 | + | chr14_fcluster1044 | 38672018-38672118 | 2 |
| ENST00000561122.1 | chr15:57178369-57180122 | - | chr15_rcluster1649 | 57179840-57179886 | 3 |
| ENST00000560198.1 | chr15:38364220-38365160 | - | chr15_rcluster796 | 38364926-38364974 | 2 |
| ENST00000559781.1 | chr15:81616719-81616902 | + | chr15_fcluster2994 | 81616807-81616857 | 3 |
| ENST00000560973.1 | chr15:81616780-81616902 | + | chr15_fcluster2994 | 81616807-81616857 | 3 |
| ENST00000500949.2 | chr15:41590240-41598732 | + | chr15_fcluster1058 | 41597350-41597402 | 2 |
| ENST00000561463.1 | chr15:102292670-102305134 | + | chr15_fcluster3969 | 102295623-102295675 | 2 |
| ENST00000558342.1 | chr15:83418049-83419041 | + | chr15_fcluster3097 | 83418470-83418523 | 4 |
| ENST00000506090.1 | chr15:89128803-89130293 | - | chr15_rcluster3179 | 89129944-89129998 | 2 |
| ENST00000399971.3 | chr15:30772946-30773643 | + | chr15_fcluster537 | 30773027-30773082 | 2 |
| ENST00000500949.2 | chr15:41590240-41598732 | + | chr15_fcluster1052 | 41595257-41595312 | 2 |
| ENST00000508764.1 | chr15:28992857-28993021 | + | chr15_fcluster459 | 28992938-28992993 | 2 |
| ENST00000512149.1 | chr15:28992857-28993021 | + | chr15_fcluster459 | 28992938-28992993 | 2 |
| ENST00000515318.1 | chr15:28992857-28993021 | + | chr15_fcluster459 | 28992938-28992993 | 2 |
| ENST00000560962.1 | chr15:63892462-63893008 | - | chr15_rcluster1958 | 63892930-63892992 | 2 |
| ENST00000561191.1 | chr15:63892789-63892996 | - | chr15_rcluster1958 | 63892930-63892992 | 2 |
| ENST00000500949.2 | chr15:41590240-41598732 | + | chr15_fcluster1056 | 41596701-41596765 | 8 |
| ENST00000500949.2 | chr15:41590240-41598732 | + | chr15_fcluster1059 | 41597752-41597816 | 5 |
| ENST00000500949.2 | chr15:41590240-41598732 | + | chr15_fcluster1048 | 41593527-41593593 | 2 |
| ENST00000499326.1 | chr15:50648298-50648633 | + | chr15_fcluster1492 | 50648385-50648454 | 7 |
| ENST00000499624.2 | chr15:50648298-50650527 | + | chr15_fcluster1492 | 50648385-50648454 | 7 |
| ENST00000560187.1 | chr15:50647157-50648634 | + | chr15_fcluster1492 | 50648385-50648454 | 7 |
| ENST00000560359.1 | chr15:50648298-50648567 | + | chr15_fcluster1492 | 50648385-50648454 | 7 |
| ENST00000561289.1 | chr15:50648298-50648611 | + | chr15_fcluster1492 | 50648385-50648454 | 7 |
| ENST00000558876.1 | chr15:98535019-98535455 | - | chr15_rcluster3552 | 98535157-98535227 | 2 |
| ENST00000561463.1 | chr15:102292670-102305134 | + | chr15_fcluster3966 | 102294534-102294605 | 4 |
| ENST00000561463.1 | chr15:102292670-102305134 | + | chr15_fcluster3968 | 102294969-102295040 | 4 |
| ENST00000561463.1 | chr15:102292670-102305134 | + | chr15_fcluster3972 | 102296973-102297044 | 4 |
| ENST00000561463.1 | chr15:102292670-102305134 | + | chr15_fcluster3975 | 102298363-102298434 | 4 |
| ENST00000561463.1 | chr15:102292670-102305134 | + | chr15_fcluster3976 | 102298799-102298870 | 4 |
| ENST00000561463.1 | chr15:102292670-102305134 | + | chr15_fcluster3982 | 102301241-102301312 | 4 |
| ENST00000561463.1 | chr15:102292670-102305134 | + | chr15_fcluster3983 | 102301676-102301747 | 4 |
| ENST00000561463.1 | chr15:102292670-102305134 | + | chr15_fcluster3987 | 102303007-102303078 | 4 |
| ENST00000561463.1 | chr15:102292670-102305134 | + | chr15_fcluster3989 | 102303624-102303695 | 4 |
| ENST00000313807.4 | chr15:44828242-44829121 | - | chr15_rcluster1144 | 44829014-44829087 | 19 |
| ENST00000559356.1 | chr15:44829000-44829092 | - | chr15_rcluster1144 | 44829014-44829087 | 19 |
| ENST00000560049.1 | chr15:44829000-44829098 | - | chr15_rcluster1144 | 44829014-44829087 | 19 |
| ENST00000500949.2 | chr15:41590240-41598732 | + | chr15_fcluster1057 | 41597159-41597233 | 4 |
| ENST00000560153.1 | chr15:88121307-88122917 | + | chr15_fcluster3289 | 88121782-88121857 | 3 |
| ENST00000436697.2 | chr15:55611063-55611243 | - | chr15_rcluster1563 | 55611070-55611147 | 7 |
| ENST00000500949.2 | chr15:41590240-41598732 | + | chr15_fcluster1041 | 41590537-41590614 | 3 |
| ENST00000500949.2 | chr15:41590240-41598732 | + | chr15_fcluster1043 | 41591889-41591966 | 2 |
| ENST00000543286.1 | chr15:93855787-93856206 | + | chr15_fcluster3648 | 93856121-93856198 | 2 |
| ENST00000549804.1 | chr15:25364213-25367623 | + | chr15_fcluster287 | 25365321-25365398 | 3 |
| ENST00000552334.1 | chr15:25277021-25281637 | + | chr15_fcluster238 | 25277113-25277190 | 3 |
| ENST00000554466.1 | chr15:93855787-93856206 | + | chr15_fcluster3648 | 93856121-93856198 | 2 |
| ENST00000558675.1 | chr15:40213272-40218081 | + | chr15_fcluster936 | 40217865-40217942 | 3 |
| ENST00000558945.1 | chr15:41590240-41590979 | + | chr15_fcluster1041 | 41590537-41590614 | 3 |
| ENST00000500691.1 | chr15:51183054-51183274 | - | chr15_rcluster1382 | 51183181-51183262 | 2 |
| ENST00000500949.2 | chr15:41590240-41598732 | + | chr15_fcluster1060 | 41598424-41598505 | 2 |
| ENST00000414175.1 | chr15:25438281-25438636 | + | chr15_fcluster304 | 25438467-25438549 | 70 |
| ENST00000424208.1 | chr15:25438281-25438556 | + | chr15_fcluster304 | 25438467-25438549 | 70 |
| ENST00000456576.1 | chr15:25438461-25438838 | + | chr15_fcluster304 | 25438467-25438549 | 70 |
| ENST00000500191.1 | chr15:57598617-57599266 | + | chr15_fcluster1777 | 57599150-57599233 | 2 |
| ENST00000501726.1 | chr15:57597518-57599266 | + | chr15_fcluster1777 | 57599150-57599233 | 2 |
| ENST00000560153.1 | chr15:88120161-88120278 | + | chr15_fcluster3288 | 88120162-88120245 | 6 |
| ENST00000504245.1 | chr15:40357423-40359491 | + | chr15_fcluster949 | 40357605-40357689 | 2 |
| ENST00000557790.1 | chr15:79575992-79576287 | - | chr15_rcluster2784 | 79576172-79576256 | 4 |
| ENST00000558297.1 | chr15:79575992-79576287 | - | chr15_rcluster2784 | 79576172-79576256 | 4 |
| ENST00000559012.1 | chr15:40357423-40357906 | + | chr15_fcluster949 | 40357605-40357689 | 2 |
| ENST00000500941.1 | chr15:32906639-32907361 | - | chr15_rcluster565 | 32907035-32907121 | 8 |
| ENST00000501830.1 | chr15:30917255-30917976 | - | chr15_rcluster470 | 30917651-30917737 | 8 |
| ENST00000560068.1 | chr15:101453409-101456185 | - | chr15_rcluster3668 | 101454549-101454635 | 3 |
| ENST00000313807.4 | chr15:44828242-44829121 | - | chr15_rcluster1142 | 44828612-44828699 | 2 |
| ENST00000560750.1 | chr15:44828242-44828838 | - | chr15_rcluster1142 | 44828612-44828699 | 2 |
| ENST00000549804.1 | chr15:25364213-25367623 | + | chr15_fcluster286 | 25364818-25364907 | 2 |
| ENST00000559555.1 | chr15:64983634-64983790 | - | chr15_rcluster2027 | 64983656-64983746 | 2 |
| ENST00000560837.1 | chr15:64983634-64983790 | - | chr15_rcluster2027 | 64983656-64983746 | 2 |
| ENST00000454486.2 | chr15:32818482-32819179 | - | chr15_rcluster558 | 32819005-32819096 | 3 |
| ENST00000500949.2 | chr15:41590240-41598732 | + | chr15_fcluster1045 | 41592391-41592483 | 3 |
| ENST00000546682.1 | chr15:25354703-25359074 | + | chr15_fcluster284 | 25354845-25354937 | 2 |
| ENST00000458245.4 | chr15:45694098-45694525 | - | chr15_rcluster1201 | 45694352-45694446 | 4 |
| ENST00000313807.4 | chr15:44828242-44829121 | - | chr15_rcluster1141 | 44828310-44828405 | 3 |
| ENST00000558963.1 | chr15:36733258-36733895 | - | chr15_rcluster745 | 36733797-36733892 | 5 |
| ENST00000560049.1 | chr15:44828242-44828556 | - | chr15_rcluster1141 | 44828310-44828405 | 3 |
| ENST00000560750.1 | chr15:44828242-44828838 | - | chr15_rcluster1141 | 44828310-44828405 | 3 |
| ENST00000561463.1 | chr15:102292670-102305134 | + | chr15_fcluster3980 | 102300161-102300260 | 6 |
| ENST00000560743.1 | chr15:39480640-39482367 | - | chr15_rcluster823 | 39481120-39481220 | 4 |
| ENST00000339021.2 | chr16:1358432-1359414 | - | chr16_rcluster253 | 1358778-1358824 | 2 |
| ENST00000500351.1 | chr16:4303649-4303790 | - | chr16_rcluster854 | 4303708-4303756 | 9 |
| ENST00000507031.1 | chr16:28827458-28829149 | + | chr16_fcluster4069 | 28827990-28828038 | 2 |
| ENST00000380148.2 | chr16:32265143-32265308 | + | chr16_fcluster4742 | 32265172-32265223 | 12 |
| ENST00000329244.5 | chr16:72911-73085 | + | chr16_fcluster2 | 72965-73017 | 3 |
| ENST00000527434.1 | chr16:72911-73085 | + | chr16_fcluster2 | 72965-73017 | 3 |
| ENST00000557792.1 | chr16:54962683-54963101 | - | chr16_rcluster5819 | 54962720-54962774 | 2 |
| ENST00000558952.1 | chr16:54962683-54962798 | - | chr16_rcluster5819 | 54962720-54962774 | 2 |
| ENST00000560208.1 | chr16:54962683-54963061 | - | chr16_rcluster5819 | 54962720-54962774 | 2 |
| ENST00000450909.2 | chr16:29938658-29939003 | + | chr16_fcluster4298 | 29938944-29939000 | 4 |
| ENST00000552015.1 | chr16:15248761-15250877 | + | chr16_fcluster2297 | 15248806-15248865 | 4 |
| ENST00000546674.1 | chr16:14995357-14996031 | + | chr16_fcluster2200 | 14995377-14995437 | 68 |
| ENST00000547549.1 | chr16:16394008-16394683 | + | chr16_fcluster2489 | 16394028-16394088 | 68 |
| ENST00000537498.1 | chr16:89118870-89119373 | - | chr16_rcluster9754 | 89119293-89119355 | 3 |
| ENST00000450909.2 | chr16:29938658-29939003 | + | chr16_fcluster4297 | 29938703-29938770 | 3 |
| ENST00000546612.1 | chr16:16402400-16403857 | - | chr16_rcluster2334 | 16403562-16403632 | 6 |
| ENST00000548268.1 | chr16:15003745-15005202 | - | chr16_rcluster2062 | 15004907-15004977 | 6 |
| ENST00000549796.1 | chr16:18496004-18497461 | + | chr16_fcluster2744 | 18496227-18496297 | 6 |
| ENST00000501143.1 | chr16:66785656-66786803 | + | chr16_fcluster6940 | 66785834-66785907 | 7 |
| ENST00000501801.1 | chr16:29759298-29761151 | + | chr16_fcluster4249 | 29760915-29760988 | 2 |
| ENST00000546612.1 | chr16:16402400-16403857 | - | chr16_rcluster2335 | 16403751-16403824 | 6 |
| ENST00000548268.1 | chr16:15003745-15005202 | - | chr16_rcluster2063 | 15005096-15005169 | 6 |
| ENST00000549796.1 | chr16:18496004-18497461 | + | chr16_fcluster2743 | 18496035-18496108 | 6 |
| ENST00000486926.1 | chr16:30593046-30593469 | + | chr16_fcluster4444 | 30593131-30593212 | 2 |
| ENST00000254109.5 | chr16:31711912-31712059 | + | chr16_fcluster4680 | 31711944-31712028 | 3 |
| ENST00000525610.1 | chr16:31711917-31712059 | + | chr16_fcluster4680 | 31711944-31712028 | 3 |
| ENST00000500351.1 | chr16:4301324-4302035 | - | chr16_rcluster852 | 4301357-4301443 | 9 |
| ENST00000530512.2 | chr16:75529159-75529305 | + | chr16_fcluster8351 | 75529176-75529262 | 23 |
| ENST00000499966.1 | chr16:66442428-66442717 | + | chr16_fcluster6876 | 66442442-66442536 | 4 |
| ENST00000546612.1 | chr16:16402400-16403857 | - | chr16_rcluster2332 | 16402855-16402949 | 3 |
| ENST00000548268.1 | chr16:15003745-15005202 | - | chr16_rcluster2060 | 15004200-15004294 | 3 |
| ENST00000549796.1 | chr16:18496004-18497461 | + | chr16_fcluster2746 | 18496910-18497004 | 3 |
| ENST00000552015.1 | chr16:15248761-15250877 | + | chr16_fcluster2299 | 15249667-15249761 | 3 |
| ENST00000501311.1 | chr16:86508136-86510805 | - | chr16_rcluster9405 | 86509711-86509807 | 3 |
| ENST00000339021.2 | chr16:1358432-1359414 | - | chr16_rcluster254 | 1358897-1358994 | 2 |
| ENST00000468219.1 | chr16:19320327-19322269 | + | chr16_fcluster2858 | 19321752-19321851 | 2 |
| ENST00000546612.1 | chr16:16402400-16403857 | - | chr16_rcluster2333 | 16403372-16403471 | 3 |
| ENST00000548268.1 | chr16:15003745-15005202 | - | chr16_rcluster2061 | 15004717-15004816 | 3 |
| ENST00000549796.1 | chr16:18496004-18497461 | + | chr16_fcluster2745 | 18496388-18496487 | 3 |
| ENST00000552015.1 | chr16:15248761-15250877 | + | chr16_fcluster2298 | 15249145-15249244 | 3 |
| ENST00000412260.1 | chr17:18414577-18416772 | - | chr17_rcluster9346 | 18416191-18416236 | 2 |
| ENST00000425211.1 | chr17:18414536-18416772 | - | chr17_rcluster9346 | 18416191-18416236 | 2 |
| ENST00000533232.1 | chr17:70076080-70077542 | - | chr17_rcluster32687 | 70077304-70077349 | 6 |
| ENST00000421796.1 | chr17:19351429-19351880 | - | chr17_rcluster9762 | 19351824-19351870 | 2 |
| ENST00000458392.1 | chr17:44636197-44638051 | - | chr17_rcluster18679 | 44636504-44636550 | 3 |
| ENST00000313495.1 | chr17:12538319-12540504 | + | chr17_fcluster6990 | 12539705-12539752 | 3 |
| ENST00000399011.2 | chr17:21147420-21147537 | - | chr17_rcluster10611 | 21147480-21147527 | 2 |
| ENST00000430983.1 | chr17:62962669-62964499 | - | chr17_rcluster28402 | 62963888-62963935 | 2 |
| ENST00000468196.1 | chr17:21147420-21147537 | - | chr17_rcluster10611 | 21147480-21147527 | 2 |
| ENST00000499972.1 | chr17:45132468-45134119 | - | chr17_rcluster18814 | 45133384-45133431 | 3 |
| ENST00000560400.1 | chr17:40004771-40007699 | - | chr17_rcluster16623 | 40006512-40006559 | 2 |
| ENST00000501190.1 | chr17:58072982-58074333 | + | chr17_fcluster23975 | 58073869-58073917 | 4 |
| ENST00000500767.2 | chr17:65871896-65874297 | - | chr17_rcluster30590 | 65873976-65874025 | 2 |
| ENST00000501718.2 | chr17:49020759-49022074 | - | chr17_rcluster20100 | 49021930-49021979 | 6 |
| ENST00000397713.1 | chr17:62970674-62971694 | - | chr17_rcluster28412 | 62971518-62971568 | 6 |
| ENST00000430983.1 | chr17:62970674-62971686 | - | chr17_rcluster28412 | 62971518-62971568 | 6 |
| ENST00000397713.1 | chr17:62964157-62964499 | - | chr17_rcluster28403 | 62964238-62964292 | 2 |
| ENST00000430983.1 | chr17:62962669-62964499 | - | chr17_rcluster28403 | 62964238-62964292 | 2 |
| ENST00000456090.2 | chr17:17579525-17579685 | - | chr17_rcluster9003 | 17579564-17579618 | 2 |
| ENST00000514358.1 | chr17:48986198-48986317 | + | chr17_fcluster19883 | 48986198-48986252 | 3 |
| ENST00000519432.1 | chr17:48986040-48986317 | + | chr17_fcluster19883 | 48986198-48986252 | 3 |
| ENST00000523470.1 | chr17:48986198-48986284 | + | chr17_fcluster19883 | 48986198-48986252 | 3 |
| ENST00000434411.1 | chr17:75089327-75091068 | + | chr17_fcluster36725 | 75089588-75089644 | 2 |
| ENST00000499745.1 | chr17:80247923-80249439 | - | chr17_rcluster39619 | 80248923-80248980 | 3 |
| ENST00000548801.1 | chr17:46656993-46659621 | - | chr17_rcluster19338 | 46659341-46659398 | 3 |
| ENST00000438772.2 | chr17:46710877-46714135 | - | chr17_rcluster19357 | 46711641-46711699 | 2 |
| ENST00000449363.1 | chr17:13932610-13933888 | - | chr17_rcluster7577 | 13933513-13933571 | 2 |
| ENST00000499670.2 | chr17:72206120-72207821 | - | chr17_rcluster33904 | 72206240-72206298 | 6 |
| ENST00000321800.7 | chr17:71746470-71746873 | - | chr17_rcluster33712 | 71746690-71746750 | 2 |
| ENST00000425081.1 | chr17:1421013-1421389 | + | chr17_fcluster953 | 1421297-1421357 | 4 |
| ENST00000444747.2 | chr17:71745410-71746776 | - | chr17_rcluster33712 | 71746690-71746750 | 2 |
| ENST00000508570.1 | chr17:80172104-80173498 | - | chr17_rcluster39575 | 80172547-80172607 | 5 |
| ENST00000457958.1 | chr17:70399470-70400957 | - | chr17_rcluster32816 | 70399990-70400051 | 2 |
| ENST00000293215.6 | chr17:73996223-73997622 | + | chr17_fcluster35631 | 73997392-73997454 | 6 |
| ENST00000426803.1 | chr17:73996223-73997622 | + | chr17_fcluster35631 | 73997392-73997454 | 6 |
| ENST00000433702.1 | chr17:41380821-41381062 | - | chr17_rcluster17326 | 41380839-41380901 | 3 |
| ENST00000307229.3 | chr17:5403352-5404465 | - | chr17_rcluster3941 | 5403845-5403908 | 3 |
| ENST00000434411.1 | chr17:75089327-75091068 | + | chr17_fcluster36726 | 75090278-75090342 | 7 |
| ENST00000413077.1 | chr17:5015228-5015413 | + | chr17_fcluster3550 | 5015296-5015362 | 5 |
| ENST00000307229.3 | chr17:5403352-5404465 | - | chr17_rcluster3942 | 5404103-5404171 | 4 |
| ENST00000499710.1 | chr17:56156724-56159977 | - | chr17_rcluster22230 | 56159182-56159250 | 6 |
| ENST00000444070.1 | chr17:18323283-18323497 | + | chr17_fcluster9135 | 18323364-18323433 | 7 |
| ENST00000499745.1 | chr17:80250400-80250690 | - | chr17_rcluster39621 | 80250603-80250673 | 72 |
| ENST00000412360.1 | chr17:50939482-50939831 | + | chr17_fcluster20328 | 50939604-50939676 | 2 |
| ENST00000422730.2 | chr17:46801691-46802819 | + | chr17_fcluster19217 | 46802634-46802707 | 3 |
| ENST00000500767.2 | chr17:65871896-65874297 | - | chr17_rcluster30589 | 65873791-65873864 | 6 |
| ENST00000514726.1 | chr17:49411708-49412526 | - | chr17_rcluster20283 | 49412087-49412160 | 2 |
| ENST00000311434.9 | chr17:8731885-8732307 | - | chr17_rcluster5728 | 8732124-8732198 | 7 |
| ENST00000399376.3 | chr17:8731885-8732307 | - | chr17_rcluster5728 | 8732124-8732198 | 7 |
| ENST00000434064.2 | chr17:8731885-8732307 | - | chr17_rcluster5728 | 8732124-8732198 | 7 |
| ENST00000452122.1 | chr17:8731885-8732307 | - | chr17_rcluster5728 | 8732124-8732198 | 7 |
| ENST00000560400.1 | chr17:40004771-40007699 | - | chr17_rcluster16624 | 40006637-40006714 | 8 |
| ENST00000439041.1 | chr17:76340047-76340342 | + | chr17_fcluster37659 | 76340052-76340130 | 4 |
| ENST00000500767.2 | chr17:65880526-65881544 | - | chr17_rcluster30598 | 65880724-65880802 | 4 |
| ENST00000507040.1 | chr17:77889985-77893635 | - | chr17_rcluster38264 | 77891635-77891713 | 3 |
| ENST00000426803.1 | chr17:73998593-73999479 | + | chr17_fcluster35633 | 73999014-73999093 | 10 |
| ENST00000285176.8 | chr17:18486656-18486837 | - | chr17_rcluster9373 | 18486686-18486766 | 2 |
| ENST00000315707.3 | chr17:8123961-8124827 | - | chr17_rcluster5396 | 8124717-8124797 | 3 |
| ENST00000414432.1 | chr17:18417387-18419084 | - | chr17_rcluster9348 | 18418325-18418405 | 6 |
| ENST00000457330.1 | chr17:18486656-18486837 | - | chr17_rcluster9373 | 18486686-18486766 | 2 |
| ENST00000501501.2 | chr17:48226509-48227991 | - | chr17_rcluster19850 | 48227882-48227963 | 5 |
| ENST00000546578.1 | chr17:46709838-46709938 | - | chr17_rcluster19356 | 46709855-46709936 | 12285 |
| ENST00000414744.1 | chr17:26925809-26926857 | + | chr17_fcluster11494 | 26926191-26926273 | 12 |
| ENST00000429292.2 | chr17:30770535-30772564 | - | chr17_rcluster13457 | 30771696-30771778 | 2 |
| ENST00000311434.9 | chr17:8726705-8726814 | - | chr17_rcluster5725 | 8726719-8726802 | 2 |
| ENST00000399376.3 | chr17:8726705-8726814 | - | chr17_rcluster5725 | 8726719-8726802 | 2 |
| ENST00000426803.1 | chr17:73998593-73999479 | + | chr17_fcluster35632 | 73998816-73998899 | 19 |
| ENST00000434064.2 | chr17:8726705-8726814 | - | chr17_rcluster5725 | 8726719-8726802 | 2 |
| ENST00000452122.1 | chr17:8726705-8726814 | - | chr17_rcluster5725 | 8726719-8726802 | 2 |
| ENST00000421108.1 | chr17:18419840-18419931 | - | chr17_rcluster9350 | 18419846-18419930 | 2 |
| ENST00000444070.1 | chr17:18323648-18324330 | + | chr17_fcluster9136 | 18324096-18324180 | 2 |
| ENST00000445433.1 | chr17:18323648-18324330 | + | chr17_fcluster9136 | 18324096-18324180 | 2 |
| ENST00000334146.3 | chr17:1616998-1617308 | - | chr17_rcluster1223 | 1617203-1617288 | 148108 |
| ENST00000543040.1 | chr17:60885862-60886312 | + | chr17_fcluster26480 | 60885895-60885980 | 2 |
| ENST00000502258.1 | chr17:43357853-43359584 | + | chr17_fcluster18108 | 43358522-43358609 | 3 |
| ENST00000548801.1 | chr17:46656993-46659621 | - | chr17_rcluster19337 | 46657224-46657311 | 145348 |
| ENST00000414432.1 | chr17:18417387-18419084 | - | chr17_rcluster9349 | 18418637-18418726 | 4 |
| ENST00000515692.1 | chr17:47977900-47978257 | + | chr17_fcluster19621 | 47978133-47978223 | 3 |
| ENST00000293215.6 | chr17:73975342-73975592 | + | chr17_fcluster35594 | 73975383-73975474 | 2 |
| ENST00000426803.1 | chr17:73975313-73975592 | + | chr17_fcluster35594 | 73975383-73975474 | 2 |
| ENST00000433856.1 | chr17:67621648-67624492 | + | chr17_fcluster31682 | 67622733-67622824 | 9 |
| ENST00000341745.3 | chr17:16705574-16707769 | + | chr17_fcluster8444 | 16706661-16706753 | 2 |
| ENST00000393005.2 | chr17:16705574-16707769 | + | chr17_fcluster8444 | 16706661-16706753 | 2 |
| ENST00000437217.1 | chr17:16705574-16707769 | + | chr17_fcluster8444 | 16706661-16706753 | 2 |
| ENST00000476204.1 | chr17:46667783-46667951 | + | chr17_fcluster19188 | 46667785-46667877 | 9 |
| ENST00000545206.1 | chr17:78940040-78942745 | - | chr17_rcluster38811 | 78942320-78942413 | 2 |
| ENST00000285176.8 | chr17:18457288-18457580 | - | chr17_rcluster9367 | 18457386-18457481 | 4 |
| ENST00000457330.1 | chr17:18457288-18457580 | - | chr17_rcluster9367 | 18457386-18457481 | 4 |
| ENST00000560400.1 | chr17:40004771-40007699 | - | chr17_rcluster16622 | 40006344-40006440 | 3 |
| ENST00000433856.1 | chr17:67621648-67624492 | + | chr17_fcluster31683 | 67623188-67623285 | 15 |
| ENST00000540200.1 | chr17:26673660-26675257 | - | chr17_rcluster11498 | 26673663-26673760 | 2 |
| ENST00000470491.1 | chr17:16342374-16342728 | + | chr17_fcluster8304 | 16342612-16342710 | 9 |
| ENST00000472293.1 | chr17:16342353-16342728 | + | chr17_fcluster8304 | 16342612-16342710 | 9 |
| ENST00000475953.1 | chr17:16342360-16342728 | + | chr17_fcluster8304 | 16342612-16342710 | 9 |
| ENST00000499503.1 | chr17:80200544-80202800 | + | chr17_fcluster39986 | 80202365-80202464 | 3 |
| ENST00000382649.2 | chr18:72263665-72265071 | - | chr18_rcluster2273 | 72264336-72264381 | 2 |
| ENST00000382649.2 | chr18:72263665-72265071 | - | chr18_rcluster2274 | 72264578-72264628 | 4 |
| ENST00000445581.2 | chr18:3606553-3608312 | + | chr18_fcluster179 | 3608047-3608097 | 2 |
| ENST00000501730.1 | chr18:19748629-19748929 | - | chr18_rcluster628 | 19748821-19748898 | 2 |
| ENST00000424522.1 | chr19:36106130-36106288 | + | chr19_fcluster2567 | 36106239-36106288 | 2 |
| ENST00000428854.1 | chr19:36106130-36106288 | + | chr19_fcluster2567 | 36106239-36106288 | 2 |
| ENST00000448051.1 | chr19:37957987-37958192 | - | chr19_rcluster2654 | 37958056-37958109 | 4 |
| ENST00000430024.1 | chr19:12267372-12267546 | - | chr19_rcluster1220 | 12267481-12267536 | 7 |
| ENST00000434822.1 | chr19:12267372-12267546 | - | chr19_rcluster1220 | 12267481-12267536 | 7 |
| ENST00000498944.1 | chr19:44405190-44405957 | - | chr19_rcluster3125 | 44405887-44405946 | 2 |
| ENST00000422045.1 | chr19:54369189-54369394 | + | chr19_fcluster4300 | 54369267-54369330 | 2 |
| ENST00000446262.1 | chr19:37019221-37019383 | + | chr19_fcluster2678 | 37019298-37019363 | 4 |
| ENST00000433059.1 | chr19:36281920-36282754 | - | chr19_rcluster2504 | 36282176-36282242 | 2 |
| ENST00000499062.1 | chr19:58789890-58790174 | - | chr19_rcluster4325 | 58790014-58790083 | 3 |
| ENST00000501448.1 | chr19:1394804-1396466 | - | chr19_rcluster204 | 1394974-1395048 | 4 |
| ENST00000515410.1 | chr19:18314707-18314845 | - | chr19_rcluster1733 | 18314741-18314820 | 2 |
| ENST00000376230.3 | chr19:58071025-58071231 | - | chr19_rcluster4269 | 58071144-58071228 | 7 |
| ENST00000447310.1 | chr19:58071025-58071231 | - | chr19_rcluster4269 | 58071144-58071228 | 7 |
| ENST00000457177.1 | chr19:58071025-58071231 | - | chr19_rcluster4269 | 58071144-58071228 | 7 |
| ENST00000334095.4 | chr19:53957905-53961514 | + | chr19_fcluster4210 | 53959572-53959663 | 3 |
| ENST00000429310.1 | chr19:53957905-53961515 | + | chr19_fcluster4210 | 53959572-53959663 | 3 |
| ENST00000432094.2 | chr19:53957902-53959864 | + | chr19_fcluster4210 | 53959572-53959663 | 3 |
| ENST00000454407.1 | chr19:53957902-53961514 | + | chr19_fcluster4210 | 53959572-53959663 | 3 |
| ENST00000515410.1 | chr19:18313149-18313550 | - | chr19_rcluster1732 | 18313330-18313422 | 10 |
| ENST00000334095.4 | chr19:53957905-53961514 | + | chr19_fcluster4211 | 53960643-53960740 | 6 |
| ENST00000429310.1 | chr19:53957905-53961515 | + | chr19_fcluster4211 | 53960643-53960740 | 6 |
| ENST00000454407.1 | chr19:53957902-53961514 | + | chr19_fcluster4211 | 53960643-53960740 | 6 |
| ENST00000500322.1 | chr19:52900716-52900985 | - | chr19_rcluster3862 | 52900883-52900980 | 2 |
| ENST00000501032.1 | chr19:52900716-52901010 | - | chr19_rcluster3862 | 52900883-52900980 | 2 |
| ENST00000420644.1 | chr2:39220552-39220998 | - | chr2_rcluster1842 | 39220593-39220638 | 3 |
| ENST00000447070.1 | chr2:27579114-27579723 | + | chr2_fcluster1205 | 27579145-27579191 | 6 |
| ENST00000435627.1 | chr2:206950247-206950527 | + | chr2_fcluster9129 | 206950404-206950452 | 7 |
| ENST00000451164.1 | chr2:208463805-208464480 | + | chr2_fcluster9207 | 208464270-208464318 | 3 |
| ENST00000414876.1 | chr2:231851700-231852894 | - | chr2_rcluster10220 | 231852788-231852838 | 2 |
| ENST00000418330.1 | chr2:231852695-231852894 | - | chr2_rcluster10220 | 231852788-231852838 | 2 |
| ENST00000422128.1 | chr2:39664544-39664676 | + | chr2_fcluster1771 | 39664587-39664637 | 8 |
| ENST00000422761.1 | chr2:71168958-71169563 | - | chr2_rcluster3365 | 71169091-71169141 | 3 |
| ENST00000426904.1 | chr2:231852761-231852894 | - | chr2_rcluster10220 | 231852788-231852838 | 2 |
| ENST00000434094.1 | chr2:231852509-231852894 | - | chr2_rcluster10220 | 231852788-231852838 | 2 |
| ENST00000443038.1 | chr2:39664511-39664676 | + | chr2_fcluster1771 | 39664587-39664637 | 8 |
| ENST00000445520.1 | chr2:39664573-39664676 | + | chr2_fcluster1771 | 39664587-39664637 | 8 |
| ENST00000446741.1 | chr2:231852358-231852894 | - | chr2_rcluster10220 | 231852788-231852838 | 2 |
| ENST00000447639.1 | chr2:71168948-71169563 | - | chr2_rcluster3365 | 71169091-71169141 | 3 |
| ENST00000449569.1 | chr2:39664558-39664676 | + | chr2_fcluster1771 | 39664587-39664637 | 8 |
| ENST00000421083.1 | chr2:145277422-145277677 | + | chr2_fcluster6626 | 145277424-145277478 | 3 |
| ENST00000436132.1 | chr2:142888748-142889095 | - | chr2_rcluster6634 | 142888909-142888963 | 2 |
| ENST00000449714.1 | chr2:150443711-150444364 | + | chr2_fcluster6804 | 150443975-150444030 | 5 |
| ENST00000458314.1 | chr2:173328991-173329302 | - | chr2_rcluster7775 | 173329193-173329248 | 2 |
| ENST00000457694.1 | chr2:217277149-217277419 | - | chr2_rcluster9546 | 217277313-217277370 | 2 |
| ENST00000417038.1 | chr2:175352132-175352257 | + | chr2_fcluster7792 | 175352150-175352209 | 5 |
| ENST00000444196.1 | chr2:175352118-175352555 | + | chr2_fcluster7792 | 175352150-175352209 | 5 |
| ENST00000478468.1 | chr2:9788982-9789568 | + | chr2_fcluster405 | 9789445-9789505 | 2 |
| ENST00000455121.3 | chr2:86116404-86116926 | + | chr2_fcluster3903 | 86116609-86116670 | 2 |
| ENST00000502627.1 | chr2:87757774-87758508 | + | chr2_fcluster4037 | 87757950-87758012 | 3 |
| ENST00000366278.2 | chr2:86042254-86042478 | + | chr2_fcluster3901 | 86042406-86042469 | 4 |
| ENST00000441749.1 | chr2:219232315-219232558 | - | chr2_rcluster9654 | 219232491-219232554 | 9 |
| ENST00000457647.1 | chr2:108439520-108440641 | - | chr2_rcluster5068 | 108440097-108440161 | 2 |
| ENST00000457813.1 | chr2:2734549-2734729 | - | chr2_rcluster141 | 2734553-2734617 | 3 |
| ENST00000447070.1 | chr2:27579114-27579723 | + | chr2_fcluster1206 | 27579392-27579457 | 6 |
| ENST00000421534.1 | chr2:96986875-96987469 | - | chr2_rcluster4532 | 96986963-96987029 | 2 |
| ENST00000453008.2 | chr2:88927058-88928250 | + | chr2_fcluster4109 | 88927751-88927820 | 2 |
| ENST00000449451.2 | chr2:47713154-47714936 | - | chr2_rcluster2276 | 47714756-47714827 | 3 |
| ENST00000454040.1 | chr2:193640305-193641625 | + | chr2_fcluster8529 | 193641311-193641382 | 2 |
| ENST00000303002.5 | chr2:110674085-110674233 | + | chr2_fcluster5109 | 110674137-110674209 | 3 |
| ENST00000438697.2 | chr2:110674085-110674233 | + | chr2_fcluster5109 | 110674137-110674209 | 3 |
| ENST00000457336.1 | chr2:113401677-113403267 | - | chr2_rcluster5385 | 113403167-113403239 | 2 |
| ENST00000478627.1 | chr2:111212429-111212577 | - | chr2_rcluster5180 | 111212451-111212523 | 3 |
| ENST00000537472.1 | chr2:110674085-110674233 | + | chr2_fcluster5109 | 110674137-110674209 | 3 |
| ENST00000547957.1 | chr2:111212429-111212577 | - | chr2_rcluster5180 | 111212451-111212523 | 3 |
| ENST00000412065.1 | chr2:211036243-211036553 | + | chr2_fcluster9340 | 211036251-211036324 | 3 |
| ENST00000424116.1 | chr2:192711280-192711497 | + | chr2_fcluster8508 | 192711288-192711361 | 2 |
| ENST00000393525.3 | chr2:89100617-89104394 | + | chr2_fcluster4122 | 89102850-89102925 | 2 |
| ENST00000428541.1 | chr2:178129088-178129449 | + | chr2_fcluster7932 | 178129338-178129414 | 10 |
| ENST00000414416.2 | chr2:110969793-110970452 | - | chr2_rcluster5170 | 110970021-110970098 | 4 |
| ENST00000426713.1 | chr2:110969793-110970127 | - | chr2_rcluster5170 | 110970021-110970098 | 4 |
| ENST00000500257.1 | chr2:101768123-101769221 | + | chr2_fcluster4680 | 101768165-101768242 | 4 |
| ENST00000417922.1 | chr2:216708050-216708146 | - | chr2_rcluster9513 | 216708058-216708136 | 4 |
| ENST00000429985.1 | chr2:27294195-27294517 | - | chr2_rcluster1267 | 27294241-27294319 | 2 |
| ENST00000445174.1 | chr2:216708050-216708259 | - | chr2_rcluster9513 | 216708058-216708136 | 4 |
| ENST00000447619.1 | chr2:27294195-27294331 | - | chr2_rcluster1267 | 27294241-27294319 | 2 |
| ENST00000456793.1 | chr2:27294195-27294331 | - | chr2_rcluster1267 | 27294241-27294319 | 2 |
| ENST00000457336.1 | chr2:113401677-113403267 | - | chr2_rcluster5384 | 113402809-113402887 | 7 |
| ENST00000414911.1 | chr2:139258890-139259244 | - | chr2_rcluster6527 | 139259132-139259211 | 13 |
| ENST00000431985.1 | chr2:139258890-139259268 | - | chr2_rcluster6527 | 139259132-139259211 | 13 |
| ENST00000438436.1 | chr2:3607039-3607319 | + | chr2_fcluster151 | 3607040-3607119 | 4 |
| ENST00000303002.5 | chr2:110656010-110656487 | + | chr2_fcluster5106 | 110656276-110656357 | 5 |
| ENST00000438697.2 | chr2:110656225-110656487 | + | chr2_fcluster5106 | 110656276-110656357 | 5 |
| ENST00000449891.1 | chr2:238875657-238875774 | + | chr2_fcluster10680 | 238875667-238875748 | 12 |
| ENST00000479168.1 | chr2:110656248-110656487 | + | chr2_fcluster5106 | 110656276-110656357 | 5 |
| ENST00000537472.1 | chr2:110656269-110656487 | + | chr2_fcluster5106 | 110656276-110656357 | 5 |
| ENST00000547957.1 | chr2:111230175-111230652 | - | chr2_rcluster5183 | 111230303-111230384 | 5 |
| ENST00000429985.1 | chr2:27293356-27293589 | - | chr2_rcluster1266 | 27293411-27293493 | 5 |
| ENST00000447619.1 | chr2:27293341-27293610 | - | chr2_rcluster1266 | 27293411-27293493 | 5 |
| ENST00000456793.1 | chr2:27293341-27293589 | - | chr2_rcluster1266 | 27293411-27293493 | 5 |
| ENST00000413452.1 | chr2:74208263-74208488 | - | chr2_rcluster3499 | 74208340-74208423 | 3 |
| ENST00000439192.1 | chr2:74208237-74208560 | - | chr2_rcluster3499 | 74208340-74208423 | 3 |
| ENST00000447070.1 | chr2:27580676-27583123 | + | chr2_fcluster1210 | 27581164-27581247 | 2 |
| ENST00000453103.1 | chr2:74208263-74208568 | - | chr2_rcluster3499 | 74208340-74208423 | 3 |
| ENST00000452525.1 | chr2:70630067-70630268 | + | chr2_fcluster3170 | 70630087-70630171 | 2 |
| ENST00000415640.1 | chr2:39873403-39873530 | + | chr2_fcluster1776 | 39873411-39873496 | 2 |
| ENST00000420509.1 | chr2:206950682-206951288 | + | chr2_fcluster9131 | 206950926-206951013 | 10 |
| ENST00000416437.1 | chr2:134191068-134191468 | - | chr2_rcluster6318 | 134191349-134191437 | 2 |
| ENST00000420672.1 | chr2:179278667-179278840 | + | chr2_fcluster7987 | 179278671-179278759 | 13 |
| ENST00000447070.1 | chr2:27580676-27583123 | + | chr2_fcluster1211 | 27581442-27581533 | 4 |
| ENST00000414141.1 | chr2:70351169-70351557 | - | chr2_rcluster3301 | 70351293-70351385 | 2 |
| ENST00000458252.1 | chr2:111368494-111368772 | + | chr2_fcluster5138 | 111368610-111368702 | 3 |
| ENST00000400768.2 | chr2:242674533-242674913 | - | chr2_rcluster10846 | 242674737-242674830 | 3 |
| ENST00000409819.1 | chr2:202979653-202981781 | - | chr2_rcluster8930 | 202979870-202979963 | 52 |
| ENST00000441997.1 | chr2:47572041-47572213 | - | chr2_rcluster2270 | 47572107-47572200 | 4 |
| ENST00000452787.1 | chr2:201577029-201578853 | - | chr2_rcluster8831 | 201578619-201578712 | 2 |
| ENST00000343987.2 | chr2:208686810-208687493 | - | chr2_rcluster9214 | 208687316-208687411 | 5 |
| ENST00000520651.1 | chr2:190627431-190627718 | + | chr2_fcluster8396 | 190627546-190627641 | 3 |
| ENST00000426725.1 | chr2:3606169-3606588 | + | chr2_fcluster150 | 3606320-3606416 | 10 |
| ENST00000438436.1 | chr2:3606083-3606588 | + | chr2_fcluster150 | 3606320-3606416 | 10 |
| ENST00000432268.1 | chr2:112249521-112250145 | - | chr2_rcluster5263 | 112249992-112250089 | 2 |
| ENST00000449783.1 | chr2:217463360-217463747 | - | chr2_rcluster9554 | 217463529-217463626 | 3 |
| ENST00000452364.1 | chr2:101617557-101618706 | - | chr2_rcluster4776 | 101618513-101618612 | 3 |
| ENST00000429282.1 | chr2:131588552-131590689 | - | chr2_rcluster6166 | 131589543-131589643 | 4 |
| ENST00000500257.1 | chr2:101770498-101771872 | + | chr2_fcluster4681 | 101771499-101771599 | 2 |
| ENST00000505973.1 | chr2:27826008-27826174 | + | chr2_fcluster1233 | 27826065-27826165 | 2 |
| ENST00000450346.1 | chr20:22541192-22545754 | - | chr20_rcluster1045 | 22545142-22545189 | 2 |
| ENST00000435366.1 | chr20:33864149-33865100 | - | chr20_rcluster1590 | 33864741-33864791 | 3 |
| ENST00000449469.1 | chr20:37056324-37057973 | - | chr20_rcluster1879 | 37057782-37057834 | 3 |
| ENST00000500146.2 | chr20:306270-306497 | - | chr20_rcluster22 | 306348-306403 | 9 |
| ENST00000246027.8 | chr20:21106696-21106845 | + | chr20_fcluster1088 | 21106724-21106781 | 18 |
| ENST00000424111.2 | chr20:21106625-21106845 | + | chr20_fcluster1088 | 21106724-21106781 | 18 |
| ENST00000427692.2 | chr20:21106625-21106845 | + | chr20_fcluster1088 | 21106724-21106781 | 18 |
| ENST00000445992.1 | chr20:21106649-21106845 | + | chr20_fcluster1088 | 21106724-21106781 | 18 |
| ENST00000458446.2 | chr20:21106625-21106845 | + | chr20_fcluster1088 | 21106724-21106781 | 18 |
| ENST00000417346.1 | chr20:55967336-55968118 | - | chr20_rcluster3016 | 55967434-55967493 | 2 |
| ENST00000461548.1 | chr20:2448254-2448404 | - | chr20_rcluster150 | 2448270-2448329 | 7 |
| ENST00000411579.1 | chr20:62259623-62260060 | + | chr20_fcluster3615 | 62259862-62259925 | 4 |
| ENST00000449500.1 | chr20:62259544-62260177 | + | chr20_fcluster3615 | 62259862-62259925 | 4 |
| ENST00000431158.1 | chr20:62667491-62669555 | + | chr20_fcluster3694 | 62668329-62668396 | 2 |
| ENST00000444463.1 | chr20:62667491-62669555 | + | chr20_fcluster3694 | 62668329-62668396 | 2 |
| ENST00000246027.8 | chr20:21143492-21143800 | + | chr20_fcluster1093 | 21143544-21143612 | 3 |
| ENST00000411646.1 | chr20:18549881-18550207 | + | chr20_fcluster994 | 18550101-18550169 | 2 |
| ENST00000424111.2 | chr20:21143492-21143800 | + | chr20_fcluster1093 | 21143544-21143612 | 3 |
| ENST00000427692.2 | chr20:21143492-21143800 | + | chr20_fcluster1093 | 21143544-21143612 | 3 |
| ENST00000428699.1 | chr20:21143492-21143800 | + | chr20_fcluster1093 | 21143544-21143612 | 3 |
| ENST00000432487.2 | chr20:21143492-21143800 | + | chr20_fcluster1093 | 21143544-21143612 | 3 |
| ENST00000435844.1 | chr20:18549884-18550207 | + | chr20_fcluster994 | 18550101-18550169 | 2 |
| ENST00000447448.1 | chr20:21143492-21143800 | + | chr20_fcluster1093 | 21143544-21143612 | 3 |
| ENST00000457464.1 | chr20:21143492-21143800 | + | chr20_fcluster1093 | 21143544-21143612 | 3 |
| ENST00000458446.2 | chr20:21143492-21143800 | + | chr20_fcluster1093 | 21143544-21143612 | 3 |
| ENST00000537654.1 | chr20:21143492-21143729 | + | chr20_fcluster1093 | 21143544-21143612 | 3 |
| ENST00000400436.2 | chr20:37075298-37077373 | + | chr20_fcluster2031 | 37075319-37075392 | 5 |
| ENST00000453698.1 | chr20:37075222-37075655 | + | chr20_fcluster2031 | 37075319-37075392 | 5 |
| ENST00000427140.1 | chr20:57090436-57090796 | + | chr20_fcluster3234 | 57090525-57090600 | 4 |
| ENST00000371639.3 | chr20:48929838-48931459 | + | chr20_fcluster2781 | 48930065-48930143 | 2 |
| ENST00000424094.1 | chr20:57425586-57425958 | - | chr20_rcluster3110 | 57425851-57425929 | 4 |
| ENST00000431158.1 | chr20:62667017-62667400 | + | chr20_fcluster3693 | 62667303-62667383 | 2 |
| ENST00000444463.1 | chr20:62665698-62667400 | + | chr20_fcluster3693 | 62667303-62667383 | 2 |
| ENST00000559804.1 | chr20:35201316-35201794 | - | chr20_rcluster1741 | 35201655-35201735 | 4 |
| ENST00000530122.1 | chr20:57243044-57243183 | + | chr20_fcluster3247 | 57243053-57243134 | 3 |
| ENST00000454676.1 | chr20:25128594-25129426 | - | chr20_rcluster1164 | 25128596-25128685 | 3 |
| ENST00000421562.1 | chr20:43706478-43707108 | + | chr20_fcluster2385 | 43706526-43706617 | 2 |
| ENST00000441009.1 | chr21:26217094-26217243 | + | chr21_fcluster418 | 26217102-26217156 | 2 |
| ENST00000447037.1 | chr21:47671464-47671743 | - | chr21_rcluster1373 | 47671507-47671565 | 2 |
| ENST00000455028.1 | chr21:36511035-36511519 | + | chr21_fcluster788 | 36511304-36511376 | 2 |
| ENST00000430247.1 | chr21:29949598-29949702 | - | chr21_rcluster482 | 29949605-29949679 | 2 |
| ENST00000433310.1 | chr21:29949542-29949702 | - | chr21_rcluster482 | 29949605-29949679 | 2 |
| ENST00000429739.1 | chr21:43442377-43442539 | + | chr21_fcluster1084 | 43442423-43442501 | 2 |
| ENST00000456917.1 | chr21:26946206-26947480 | + | chr21_fcluster434 | 26946274-26946355 | 19659 |
| ENST00000499524.1 | chr21:47877998-47878593 | - | chr21_rcluster1396 | 47878137-47878231 | 2 |
| ENST00000397787.1 | chr21:46842328-46842576 | - | chr21_rcluster1327 | 46842390-46842485 | 2 |
| ENST00000485206.1 | chr21:46842328-46842576 | - | chr21_rcluster1327 | 46842390-46842485 | 2 |
| ENST00000433344.1 | chr21:29357360-29357614 | + | chr21_fcluster502 | 29357418-29357514 | 2 |
| ENST00000504335.1 | chr22:31498260-31500743 | + | chr22_fcluster994 | 31500339-31500386 | 2 |
| ENST00000416037.1 | chr22:42520053-42520567 | + | chr22_fcluster1817 | 42520116-42520164 | 2 |
| ENST00000416037.1 | chr22:42532209-42532723 | + | chr22_fcluster1818 | 42532272-42532320 | 2 |
| ENST00000417327.1 | chr22:42520053-42520567 | + | chr22_fcluster1817 | 42520116-42520164 | 2 |
| ENST00000439129.1 | chr22:42520053-42520567 | + | chr22_fcluster1817 | 42520116-42520164 | 2 |
| ENST00000536447.1 | chr22:42532209-42532723 | + | chr22_fcluster1818 | 42532272-42532320 | 2 |
| ENST00000456099.1 | chr22:38054263-38054384 | - | chr22_rcluster1382 | 38054267-38054318 | 3 |
| ENST00000400593.2 | chr22:17092549-17092783 | + | chr22_fcluster58 | 17092643-17092696 | 5 |
| ENST00000421151.1 | chr22:27064122-27072438 | + | chr22_fcluster736 | 27067580-27067633 | 2 |
| ENST00000423278.1 | chr22:27064122-27072438 | + | chr22_fcluster736 | 27067580-27067633 | 2 |
| ENST00000430080.1 | chr22:27064122-27072438 | + | chr22_fcluster736 | 27067580-27067633 | 2 |
| ENST00000456129.1 | chr22:27064122-27072438 | + | chr22_fcluster736 | 27067580-27067633 | 2 |
| ENST00000454636.1 | chr22:20341854-20343893 | + | chr22_fcluster265 | 20342100-20342158 | 3 |
| ENST00000421151.1 | chr22:27064122-27072438 | + | chr22_fcluster740 | 27069931-27069991 | 4 |
| ENST00000423278.1 | chr22:27064122-27072438 | + | chr22_fcluster740 | 27069931-27069991 | 4 |
| ENST00000430080.1 | chr22:27064122-27072438 | + | chr22_fcluster740 | 27069931-27069991 | 4 |
| ENST00000456129.1 | chr22:27064122-27072438 | + | chr22_fcluster740 | 27069931-27069991 | 4 |
| ENST00000519077.1 | chr22:31368911-31369587 | + | chr22_fcluster982 | 31369328-31369389 | 3 |
| ENST00000521091.1 | chr22:31368911-31369587 | + | chr22_fcluster982 | 31369328-31369389 | 3 |
| ENST00000540687.1 | chr22:31368842-31369587 | + | chr22_fcluster982 | 31369328-31369389 | 3 |
| ENST00000357802.2 | chr22:42977936-42978044 | - | chr22_rcluster1824 | 42977960-42978025 | 5 |
| ENST00000438850.1 | chr22:17097637-17098004 | + | chr22_fcluster61 | 17097698-17097769 | 3 |
| ENST00000458178.1 | chr22:22292666-22293985 | + | chr22_fcluster417 | 22292673-22292744 | 2 |
| ENST00000538634.1 | chr22:22292610-22294263 | + | chr22_fcluster417 | 22292673-22292744 | 2 |
| ENST00000519077.1 | chr22:31371157-31374831 | + | chr22_fcluster987 | 31374127-31374199 | 2 |
| ENST00000521091.1 | chr22:31371157-31374831 | + | chr22_fcluster987 | 31374127-31374199 | 2 |
| ENST00000421151.1 | chr22:27064122-27072438 | + | chr22_fcluster738 | 27069022-27069095 | 4 |
| ENST00000423278.1 | chr22:27064122-27072438 | + | chr22_fcluster738 | 27069022-27069095 | 4 |
| ENST00000430080.1 | chr22:27064122-27072438 | + | chr22_fcluster738 | 27069022-27069095 | 4 |
| ENST00000456129.1 | chr22:27064122-27072438 | + | chr22_fcluster738 | 27069022-27069095 | 4 |
| ENST00000404603.1 | chr22:24967885-24967985 | + | chr22_fcluster599 | 24967894-24967969 | 3 |
| ENST00000430449.1 | chr22:32365974-32366387 | + | chr22_fcluster1071 | 32366259-32366334 | 2 |
| ENST00000434868.1 | chr22:27254598-27256985 | + | chr22_fcluster746 | 27256866-27256942 | 2 |
| ENST00000429962.1 | chr22:21356212-21357118 | + | chr22_fcluster350 | 21356497-21356574 | 4 |
| ENST00000436079.1 | chr22:21356176-21357118 | + | chr22_fcluster350 | 21356497-21356574 | 4 |
| ENST00000421151.1 | chr22:27064122-27072438 | + | chr22_fcluster737 | 27068382-27068460 | 3 |
| ENST00000423278.1 | chr22:27064122-27072438 | + | chr22_fcluster737 | 27068382-27068460 | 3 |
| ENST00000430080.1 | chr22:27064122-27072438 | + | chr22_fcluster737 | 27068382-27068460 | 3 |
| ENST00000456129.1 | chr22:27064122-27072438 | + | chr22_fcluster737 | 27068382-27068460 | 3 |
| ENST00000421151.1 | chr22:27064122-27072438 | + | chr22_fcluster739 | 27069508-27069587 | 3 |
| ENST00000423278.1 | chr22:27064122-27072438 | + | chr22_fcluster739 | 27069508-27069587 | 3 |
| ENST00000430080.1 | chr22:27064122-27072438 | + | chr22_fcluster739 | 27069508-27069587 | 3 |
| ENST00000433014.1 | chr22:21537950-21539876 | + | chr22_fcluster361 | 21538002-21538081 | 2 |
| ENST00000451257.1 | chr22:21537561-21539876 | + | chr22_fcluster361 | 21538002-21538081 | 2 |
| ENST00000456129.1 | chr22:27064122-27072438 | + | chr22_fcluster739 | 27069508-27069587 | 3 |
| ENST00000450750.1 | chr22:43805093-43805687 | + | chr22_fcluster1906 | 43805492-43805573 | 3 |
| ENST00000415764.1 | chr22:21821552-21821903 | + | chr22_fcluster372 | 21821693-21821776 | 6 |
| ENST00000426653.1 | chr22:20377670-20378143 | + | chr22_fcluster266 | 20377933-20378016 | 6 |
| ENST00000449424.1 | chr22:21821681-21821903 | + | chr22_fcluster372 | 21821693-21821776 | 6 |
| ENST00000450925.2 | chr22:21055403-21056594 | + | chr22_fcluster325 | 21056380-21056463 | 6 |
| ENST00000456153.1 | chr22:21821682-21822057 | + | chr22_fcluster372 | 21821693-21821776 | 6 |
| ENST00000456303.1 | chr22:21820713-21821903 | + | chr22_fcluster372 | 21821693-21821776 | 6 |
| ENST00000428858.1 | chr22:28315461-28315954 | + | chr22_fcluster792 | 28315491-28315586 | 13 |
| ENST00000404603.1 | chr22:24963953-24964144 | + | chr22_fcluster598 | 24964031-24964129 | 4 |
| ENST00000458544.1 | chr22:24963953-24964144 | + | chr22_fcluster598 | 24964031-24964129 | 4 |
| ENST00000273083.3 | chr3:14530620-14535334 | - | chr3_rcluster532 | 14533890-14533935 | 83 |
| ENST00000430219.1 | chr3:14532679-14535334 | - | chr3_rcluster532 | 14533890-14533935 | 83 |
| ENST00000443613.2 | chr3:14530620-14535334 | - | chr3_rcluster532 | 14533890-14533935 | 83 |
| ENST00000541007.1 | chr3:187009012-187009324 | + | chr3_fcluster6957 | 187009140-187009186 | 3 |
| ENST00000495287.1 | chr3:138654032-138657278 | - | chr3_rcluster5344 | 138654721-138654768 | 5 |
| ENST00000460574.1 | chr3:159483177-159483532 | - | chr3_rcluster6143 | 159483415-159483463 | 2 |
| ENST00000472046.1 | chr3:63989699-63991744 | + | chr3_fcluster2636 | 63990888-63990936 | 2 |
| ENST00000461943.1 | chr3:150480125-150480325 | + | chr3_fcluster5519 | 150480228-150480277 | 3 |
| ENST00000467240.1 | chr3:108867101-108868970 | + | chr3_fcluster3905 | 108868815-108868864 | 2 |
| ENST00000477643.1 | chr3:108868653-108868973 | + | chr3_fcluster3905 | 108868815-108868864 | 2 |
| ENST00000479039.1 | chr3:108868567-108868960 | + | chr3_fcluster3905 | 108868815-108868864 | 2 |
| ENST00000414604.1 | chr3:50242702-50242781 | + | chr3_fcluster2057 | 50242705-50242755 | 6 |
| ENST00000420502.1 | chr3:50242680-50242781 | + | chr3_fcluster2057 | 50242705-50242755 | 6 |
| ENST00000427428.2 | chr3:50242680-50242781 | + | chr3_fcluster2057 | 50242705-50242755 | 6 |
| ENST00000541861.1 | chr3:50242693-50242781 | + | chr3_fcluster2057 | 50242705-50242755 | 6 |
| ENST00000545531.1 | chr3:50242702-50242781 | + | chr3_fcluster2057 | 50242705-50242755 | 6 |
| ENST00000473595.1 | chr3:161144216-161145445 | + | chr3_fcluster5951 | 161144978-161145032 | 2 |
| ENST00000479752.1 | chr3:149377166-149378106 | + | chr3_fcluster5450 | 149377828-149377884 | 2 |
| ENST00000417835.1 | chr3:14989247-14989400 | - | chr3_rcluster563 | 14989313-14989370 | 3 |
| ENST00000466156.1 | chr3:116431203-116431446 | + | chr3_fcluster4125 | 116431356-116431413 | 7 |
| ENST00000496242.1 | chr3:116431203-116431704 | + | chr3_fcluster4125 | 116431356-116431413 | 7 |
| ENST00000473756.1 | chr3:98699903-98700230 | + | chr3_fcluster3551 | 98700154-98700212 | 4 |
| ENST00000426200.1 | chr3:14989521-14989931 | - | chr3_rcluster565 | 14989829-14989889 | 3 |
| ENST00000430166.1 | chr3:14989521-14989931 | - | chr3_rcluster565 | 14989829-14989889 | 3 |
| ENST00000440556.1 | chr3:194003759-194005157 | + | chr3_fcluster7220 | 194004824-194004886 | 2 |
| ENST00000463143.1 | chr3:106959540-106959934 | + | chr3_fcluster3846 | 106959567-106959632 | 4 |
| ENST00000466734.1 | chr3:106959540-106959934 | + | chr3_fcluster3846 | 106959567-106959632 | 4 |
| ENST00000490441.1 | chr3:106959540-106959934 | + | chr3_fcluster3846 | 106959567-106959632 | 4 |
| ENST00000475393.1 | chr3:150437657-150437933 | + | chr3_fcluster5515 | 150437723-150437789 | 3 |
| ENST00000431462.2 | chr3:75719172-75721122 | - | chr3_rcluster3077 | 75720048-75720115 | 2 |
| ENST00000460586.1 | chr3:86544942-86546146 | + | chr3_fcluster3289 | 86545613-86545680 | 5 |
| ENST00000476021.1 | chr3:86041334-86043864 | - | chr3_rcluster3354 | 86042083-86042151 | 2 |
| ENST00000447691.2 | chr3:44658621-44659166 | - | chr3_rcluster1588 | 44658676-44658745 | 2 |
| ENST00000475981.1 | chr3:158287961-158288855 | - | chr3_rcluster6105 | 158288760-158288829 | 4 |
| ENST00000496693.1 | chr3:165266627-165266937 | + | chr3_fcluster6056 | 165266730-165266799 | 15 |
| ENST00000468377.1 | chr3:128220453-128221191 | + | chr3_fcluster4600 | 128220536-128220610 | 2 |
| ENST00000462531.1 | chr3:154958735-154959414 | + | chr3_fcluster5666 | 154959094-154959170 | 2 |
| ENST00000420195.1 | chr3:15295692-15299113 | + | chr3_fcluster637 | 15297421-15297499 | 2 |
| ENST00000447709.1 | chr3:173054550-173054755 | + | chr3_fcluster6354 | 173054629-173054707 | 2 |
| ENST00000449586.1 | chr3:37786924-37787450 | - | chr3_rcluster1338 | 37787318-37787396 | 2 |
| ENST00000483525.1 | chr3:70062946-70064469 | + | chr3_fcluster2806 | 70063377-70063456 | 2 |
| ENST00000488545.1 | chr3:72149940-72150108 | - | chr3_rcluster2954 | 72149960-72150039 | 3 |
| ENST00000432518.1 | chr3:28617765-28617987 | + | chr3_fcluster1014 | 28617829-28617910 | 2 |
| ENST00000464958.1 | chr3:52274195-52275113 | + | chr3_fcluster2167 | 52274290-52274371 | 2 |
| ENST00000483834.1 | chr3:52274192-52275113 | + | chr3_fcluster2167 | 52274290-52274371 | 2 |
| ENST00000484892.1 | chr3:84715552-84719007 | - | chr3_rcluster3320 | 84716333-84716414 | 8 |
| ENST00000417835.1 | chr3:14986188-14987661 | - | chr3_rcluster560 | 14986697-14986779 | 2 |
| ENST00000424349.1 | chr3:14984292-14987661 | - | chr3_rcluster560 | 14986697-14986779 | 2 |
| ENST00000426200.1 | chr3:14986201-14987661 | - | chr3_rcluster560 | 14986697-14986779 | 2 |
| ENST00000440079.1 | chr3:14986532-14987661 | - | chr3_rcluster560 | 14986697-14986779 | 2 |
| ENST00000466734.1 | chr3:106964856-106967195 | + | chr3_fcluster3847 | 106965778-106965861 | 3 |
| ENST00000424349.1 | chr3:14984292-14987661 | - | chr3_rcluster556 | 14985097-14985181 | 5 |
| ENST00000435578.1 | chr3:48487739-48488060 | - | chr3_rcluster1835 | 48487953-48488037 | 5 |
| ENST00000462931.1 | chr3:149103413-149103587 | - | chr3_rcluster5741 | 149103479-149103563 | 2 |
| ENST00000383834.2 | chr3:9435635-9438752 | - | chr3_rcluster264 | 9438626-9438716 | 4 |
| ENST00000437204.1 | chr3:50193168-50193518 | - | chr3_rcluster2049 | 50193403-50193496 | 2 |
| ENST00000466156.1 | chr3:116428817-116429268 | + | chr3_fcluster4124 | 116428992-116429086 | 3 |
| ENST00000477539.1 | chr3:116428627-116429268 | + | chr3_fcluster4124 | 116428992-116429086 | 3 |
| ENST00000496242.1 | chr3:116428817-116429268 | + | chr3_fcluster4124 | 116428992-116429086 | 3 |
| ENST00000441531.1 | chr3:38495995-38496311 | - | chr3_rcluster1378 | 38496028-38496124 | 2 |
| ENST00000417835.1 | chr3:14986188-14987661 | - | chr3_rcluster561 | 14987508-14987605 | 3 |
| ENST00000424349.1 | chr3:14984292-14987661 | - | chr3_rcluster561 | 14987508-14987605 | 3 |
| ENST00000426200.1 | chr3:14986201-14987661 | - | chr3_rcluster561 | 14987508-14987605 | 3 |
| ENST00000440079.1 | chr3:14986532-14987661 | - | chr3_rcluster561 | 14987508-14987605 | 3 |
| ENST00000412804.1 | chr3:4534469-4534847 | - | chr3_rcluster119 | 4534480-4534578 | 3 |
| ENST00000424349.1 | chr3:14984292-14987661 | - | chr3_rcluster558 | 14985675-14985773 | 2 |
| ENST00000489343.1 | chr3:132440595-132441223 | + | chr3_fcluster4819 | 132440877-132440975 | 3 |
| ENST00000503298.1 | chr3:132440595-132441223 | + | chr3_fcluster4819 | 132440877-132440975 | 3 |
| ENST00000412804.1 | chr3:4534469-4534847 | - | chr3_rcluster120 | 4534662-4534761 | 8 |
| ENST00000508202.1 | chr4:13779002-13783781 | - | chr4_rcluster910 | 13781296-13781341 | 2 |
| ENST00000505537.1 | chr4:183006008-183006237 | - | chr4_rcluster8297 | 183006184-183006231 | 3 |
| ENST00000507869.1 | chr4:183006008-183006289 | - | chr4_rcluster8297 | 183006184-183006231 | 3 |
| ENST00000509399.1 | chr4:104472085-104472474 | - | chr4_rcluster4891 | 104472299-104472346 | 5 |
| ENST00000509873.1 | chr4:144833694-144833791 | + | chr4_fcluster6802 | 144833714-144833761 | 2 |
| ENST00000512652.1 | chr4:161460407-161460992 | + | chr4_fcluster7566 | 161460930-161460977 | 3 |
| ENST00000400172.3 | chr4:154873-156324 | + | chr4_fcluster17 | 155045-155095 | 2 |
| ENST00000502662.1 | chr4:154703-156491 | + | chr4_fcluster17 | 155045-155095 | 2 |
| ENST00000507332.1 | chr4:132712366-132712637 | + | chr4_fcluster6268 | 132712508-132712558 | 9 |
| ENST00000510175.1 | chr4:154703-157779 | + | chr4_fcluster17 | 155045-155095 | 2 |
| ENST00000511079.1 | chr4:154703-156177 | + | chr4_fcluster17 | 155045-155095 | 2 |
| ENST00000514364.1 | chr4:54405261-54405515 | + | chr4_fcluster2521 | 54405443-54405494 | 2 |
| ENST00000443173.1 | chr4:53608720-53612110 | - | chr4_rcluster2496 | 53609444-53609496 | 2 |
| ENST00000500560.1 | chr4:119199915-119200292 | + | chr4_fcluster5612 | 119200116-119200168 | 5 |
| ENST00000505528.2 | chr4:21610434-21610568 | + | chr4_fcluster1215 | 21610454-21610506 | 2 |
| ENST00000508484.1 | chr4:55469379-55471344 | - | chr4_rcluster2583 | 55469764-55469817 | 10 |
| ENST00000507152.1 | chr4:165816569-165818675 | + | chr4_fcluster7742 | 165817717-165817771 | 3 |
| ENST00000515405.1 | chr4:165817617-165818137 | + | chr4_fcluster7742 | 165817717-165817771 | 3 |
| ENST00000499242.2 | chr4:7099796-7101181 | - | chr4_rcluster602 | 7100768-7100823 | 2 |
| ENST00000503051.1 | chr4:53525574-53525843 | + | chr4_fcluster2464 | 53525589-53525645 | 4 |
| ENST00000307533.6 | chr4:6675179-6676433 | + | chr4_fcluster555 | 6675820-6675879 | 10 |
| ENST00000444232.2 | chr4:6675179-6676364 | + | chr4_fcluster555 | 6675820-6675879 | 10 |
| ENST00000505731.1 | chr4:2936627-2937462 | + | chr4_fcluster317 | 2936666-2936725 | 4 |
| ENST00000507152.1 | chr4:165798157-165798559 | + | chr4_fcluster7739 | 165798428-165798488 | 3 |
| ENST00000510062.1 | chr4:165798395-165798559 | + | chr4_fcluster7739 | 165798428-165798488 | 3 |
| ENST00000515275.1 | chr4:165798347-165798559 | + | chr4_fcluster7739 | 165798428-165798488 | 3 |
| ENST00000514879.1 | chr4:106473351-106473512 | - | chr4_rcluster4985 | 106473364-106473425 | 3 |
| ENST00000511497.1 | chr4:129437013-129437153 | + | chr4_fcluster6117 | 129437041-129437105 | 3 |
| ENST00000508572.1 | chr4:66864565-66865026 | + | chr4_fcluster3101 | 66864619-66864684 | 4 |
| ENST00000512428.1 | chr4:108858504-108860277 | - | chr4_rcluster5079 | 108858880-108858945 | 2 |
| ENST00000508362.1 | chr4:120988667-120988756 | + | chr4_fcluster5723 | 120988667-120988737 | 2 |
| ENST00000508601.1 | chr4:6234588-6235663 | + | chr4_fcluster521 | 6235419-6235489 | 2 |
| ENST00000507296.1 | chr4:158558840-158559276 | + | chr4_fcluster7440 | 158558971-158559046 | 5 |
| ENST00000512637.1 | chr4:109093277-109094214 | + | chr4_fcluster5182 | 109093757-109093832 | 2 |
| ENST00000507486.1 | chr4:144105662-144105982 | - | chr4_rcluster6651 | 144105749-144105827 | 8 |
| ENST00000507826.1 | chr4:144105662-144106014 | - | chr4_rcluster6651 | 144105749-144105827 | 8 |
| ENST00000411630.2 | chr4:53578992-53579126 | + | chr4_fcluster2469 | 53578996-53579075 | 3 |
| ENST00000425653.1 | chr4:53578949-53579126 | + | chr4_fcluster2469 | 53578996-53579075 | 3 |
| ENST00000441504.1 | chr4:53578992-53579296 | + | chr4_fcluster2469 | 53578996-53579075 | 3 |
| ENST00000444958.1 | chr4:53578992-53579126 | + | chr4_fcluster2469 | 53578996-53579075 | 3 |
| ENST00000498917.2 | chr4:68566999-68567202 | + | chr4_fcluster3167 | 68567071-68567153 | 14 |
| ENST00000506606.1 | chr4:68567052-68567202 | + | chr4_fcluster3167 | 68567071-68567153 | 14 |
| ENST00000510996.1 | chr4:147164339-147164821 | + | chr4_fcluster6926 | 147164679-147164763 | 7 |
| ENST00000510785.1 | chr4:54368016-54368143 | + | chr4_fcluster2519 | 54368021-54368109 | 3 |
| ENST00000500394.2 | chr4:15003563-15003669 | - | chr4_rcluster952 | 15003571-15003660 | 3 |
| ENST00000509984.1 | chr4:109529680-109529806 | - | chr4_rcluster5114 | 109529681-109529770 | 2 |
| ENST00000499430.2 | chr4:4543859-4544352 | + | chr4_fcluster458 | 4544076-4544166 | 2 |
| ENST00000507244.1 | chr4:4543871-4544202 | + | chr4_fcluster458 | 4544076-4544166 | 2 |
| ENST00000512438.1 | chr4:4543927-4544202 | + | chr4_fcluster458 | 4544076-4544166 | 2 |
| ENST00000500526.1 | chr4:110235734-110237291 | + | chr4_fcluster5232 | 110236045-110236138 | 4 |
| ENST00000505025.1 | chr4:184909430-184909744 | + | chr4_fcluster8538 | 184909434-184909530 | 7 |
| ENST00000499502.2 | chr4:6689176-6690623 | - | chr4_rcluster556 | 6689549-6689649 | 2 |
| ENST00000501280.2 | chr5:153825299-153825410 | - | chr5_rcluster5747 | 153825320-153825365 | 8 |
| ENST00000505955.1 | chr5:72705885-72706336 | + | chr5_fcluster2658 | 72706030-72706075 | 5 |
| ENST00000519727.1 | chr5:153825299-153825379 | - | chr5_rcluster5747 | 153825320-153825365 | 8 |
| ENST00000522312.1 | chr5:153825299-153825382 | - | chr5_rcluster5747 | 153825320-153825365 | 8 |
| ENST00000524264.1 | chr5:153825299-153825373 | - | chr5_rcluster5747 | 153825320-153825365 | 8 |
| ENST00000559112.1 | chr5:50678809-50679494 | - | chr5_rcluster1729 | 50679409-50679454 | 2 |
| ENST00000507434.1 | chr5:180688224-180688385 | + | chr5_fcluster7002 | 180688228-180688274 | 6 |
| ENST00000509252.1 | chr5:180688226-180688385 | + | chr5_fcluster7002 | 180688228-180688274 | 6 |
| ENST00000514146.1 | chr5:180688214-180688385 | + | chr5_fcluster7002 | 180688228-180688274 | 6 |
| ENST00000399760.2 | chr5:17216187-17217156 | - | chr5_rcluster723 | 17216528-17216575 | 2 |
| ENST00000538380.1 | chr5:17216509-17217508 | - | chr5_rcluster723 | 17216528-17216575 | 2 |
| ENST00000509065.1 | chr5:68931795-68932778 | - | chr5_rcluster2405 | 68932052-68932100 | 3 |
| ENST00000413221.2 | chr5:111497849-111499973 | + | chr5_fcluster4018 | 111497919-111497969 | 4 |
| ENST00000427306.2 | chr5:111497401-111497999 | + | chr5_fcluster4018 | 111497919-111497969 | 4 |
| ENST00000442823.2 | chr5:111497849-111498021 | + | chr5_fcluster4018 | 111497919-111497969 | 4 |
| ENST00000505623.1 | chr5:60954302-60956006 | - | chr5_rcluster2162 | 60955566-60955616 | 6 |
| ENST00000508590.1 | chr5:111497849-111498008 | + | chr5_fcluster4018 | 111497919-111497969 | 4 |
| ENST00000501937.2 | chr5:180258366-180258806 | + | chr5_fcluster6961 | 180258674-180258725 | 2 |
| ENST00000504246.1 | chr5:87971266-87972116 | - | chr5_rcluster3195 | 87972057-87972111 | 2 |
| ENST00000509065.1 | chr5:68927363-68928538 | - | chr5_rcluster2404 | 68928354-68928408 | 2 |
| ENST00000512859.1 | chr5:81147421-81148020 | + | chr5_fcluster3035 | 81147884-81147938 | 4 |
| ENST00000500733.2 | chr5:38556889-38559279 | + | chr5_fcluster1382 | 38557257-38557312 | 2 |
| ENST00000499346.2 | chr5:127418428-127418792 | - | chr5_rcluster4489 | 127418460-127418518 | 4 |
| ENST00000501173.2 | chr5:127418428-127418767 | - | chr5_rcluster4489 | 127418460-127418518 | 4 |
| ENST00000501652.1 | chr5:127418428-127418666 | - | chr5_rcluster4489 | 127418460-127418518 | 4 |
| ENST00000508353.1 | chr5:127418428-127418573 | - | chr5_rcluster4489 | 127418460-127418518 | 4 |
| ENST00000508878.1 | chr5:127418428-127418652 | - | chr5_rcluster4489 | 127418460-127418518 | 4 |
| ENST00000534918.1 | chr5:478239-480999 | + | chr5_fcluster57 | 480542-480600 | 3 |
| ENST00000500733.2 | chr5:38556889-38559279 | + | chr5_fcluster1383 | 38557631-38557690 | 2 |
| ENST00000506629.1 | chr5:611563-611868 | - | chr5_rcluster51 | 611792-611853 | 2 |
| ENST00000508458.1 | chr5:41870394-41870484 | + | chr5_fcluster1493 | 41870415-41870476 | 2 |
| ENST00000510509.1 | chr5:41870394-41870818 | + | chr5_fcluster1493 | 41870415-41870476 | 2 |
| ENST00000538380.1 | chr5:17216509-17217508 | - | chr5_rcluster724 | 17217095-17217157 | 2 |
| ENST00000320280.7 | chr5:77771328-77774638 | + | chr5_fcluster2871 | 77771534-77771598 | 4 |
| ENST00000339292.4 | chr5:77771328-77774894 | + | chr5_fcluster2871 | 77771534-77771598 | 4 |
| ENST00000502681.2 | chr5:77771328-77771636 | + | chr5_fcluster2871 | 77771534-77771598 | 4 |
| ENST00000506858.1 | chr5:77771328-77772652 | + | chr5_fcluster2871 | 77771534-77771598 | 4 |
| ENST00000508271.2 | chr5:77771328-77771838 | + | chr5_fcluster2871 | 77771534-77771598 | 4 |
| ENST00000508822.1 | chr5:77771328-77771849 | + | chr5_fcluster2871 | 77771534-77771598 | 4 |
| ENST00000509998.1 | chr5:77771328-77771849 | + | chr5_fcluster2871 | 77771534-77771598 | 4 |
| ENST00000518260.1 | chr5:172246486-172246961 | + | chr5_fcluster6463 | 172246675-172246739 | 2 |
| ENST00000538629.1 | chr5:77771328-77776562 | + | chr5_fcluster2871 | 77771534-77771598 | 4 |
| ENST00000320280.7 | chr5:77771328-77774638 | + | chr5_fcluster2877 | 77774334-77774399 | 2 |
| ENST00000339292.4 | chr5:77771328-77774894 | + | chr5_fcluster2877 | 77774334-77774399 | 2 |
| ENST00000503504.1 | chr5:162864190-162864332 | - | chr5_rcluster6027 | 162864263-162864328 | 11 |
| ENST00000538629.1 | chr5:77771328-77776562 | + | chr5_fcluster2877 | 77774334-77774399 | 2 |
| ENST00000320280.7 | chr5:77771328-77774638 | + | chr5_fcluster2874 | 77772923-77772989 | 3 |
| ENST00000339292.4 | chr5:77771328-77774894 | + | chr5_fcluster2874 | 77772923-77772989 | 3 |
| ENST00000538629.1 | chr5:77771328-77776562 | + | chr5_fcluster2874 | 77772923-77772989 | 3 |
| ENST00000502001.2 | chr5:8460047-8460776 | + | chr5_fcluster372 | 8460131-8460198 | 4 |
| ENST00000512519.1 | chr5:38845771-38845931 | - | chr5_rcluster1371 | 38845801-38845868 | 2 |
| ENST00000513480.1 | chr5:38845771-38845870 | - | chr5_rcluster1371 | 38845801-38845868 | 2 |
| ENST00000504246.1 | chr5:87971266-87972116 | - | chr5_rcluster3194 | 87971427-87971496 | 2 |
| ENST00000314957.3 | chr5:43017177-43018826 | - | chr5_rcluster1547 | 43018022-43018093 | 8 |
| ENST00000499871.2 | chr5:43017177-43018913 | - | chr5_rcluster1547 | 43018022-43018093 | 8 |
| ENST00000518054.1 | chr5:156995030-156996106 | + | chr5_fcluster5965 | 156995504-156995576 | 35 |
| ENST00000513271.1 | chr5:176875054-176878590 | - | chr5_rcluster6584 | 176876323-176876397 | 2 |
| ENST00000499521.2 | chr5:148873878-148878856 | - | chr5_rcluster5525 | 148876315-148876391 | 3 |
| ENST00000506106.1 | chr5:56690888-56691005 | + | chr5_fcluster1919 | 56690890-56690966 | 11 |
| ENST00000458103.2 | chr5:122425768-122425994 | - | chr5_rcluster4325 | 122425816-122425894 | 2 |
| ENST00000503452.1 | chr5:44808745-44808879 | - | chr5_rcluster1634 | 44808787-44808866 | 17 |
| ENST00000453721.1 | chr5:56206004-56206541 | - | chr5_rcluster1935 | 56206250-56206330 | 2 |
| ENST00000510576.2 | chr5:151065944-151067471 | + | chr5_fcluster5724 | 151066366-151066446 | 2 |
| ENST00000512035.1 | chr5:113367-113563 | + | chr5_fcluster9 | 113393-113473 | 2 |
| ENST00000517927.1 | chr5:159912307-159914433 | + | chr5_fcluster6079 | 159912356-159912436 | 54881 |
| ENST00000412431.2 | chr5:148880683-148880816 | - | chr5_rcluster5528 | 148880696-148880779 | 3 |
| ENST00000499521.2 | chr5:148880579-148884233 | - | chr5_rcluster5528 | 148880696-148880779 | 3 |
| ENST00000534918.1 | chr5:478239-480999 | + | chr5_fcluster56 | 480164-480249 | 3 |
| ENST00000520401.1 | chr5:112200319-112200429 | + | chr5_fcluster4047 | 112200320-112200406 | 2 |
| ENST00000510509.1 | chr5:41870394-41870818 | + | chr5_fcluster1494 | 41870644-41870731 | 6 |
| ENST00000505775.1 | chr5:27477747-27477908 | + | chr5_fcluster975 | 27477778-27477866 | 2 |
| ENST00000510165.1 | chr5:27477747-27477908 | + | chr5_fcluster975 | 27477778-27477866 | 2 |
| ENST00000514255.1 | chr5:27477747-27477908 | + | chr5_fcluster975 | 27477778-27477866 | 2 |
| ENST00000320280.7 | chr5:77771328-77774638 | + | chr5_fcluster2873 | 77772042-77772131 | 9 |
| ENST00000339292.4 | chr5:77771328-77774894 | + | chr5_fcluster2873 | 77772042-77772131 | 9 |
| ENST00000506858.1 | chr5:77771328-77772652 | + | chr5_fcluster2873 | 77772042-77772131 | 9 |
| ENST00000538629.1 | chr5:77771328-77776562 | + | chr5_fcluster2873 | 77772042-77772131 | 9 |
| ENST00000320280.7 | chr5:77771328-77774638 | + | chr5_fcluster2876 | 77773861-77773954 | 3 |
| ENST00000339292.4 | chr5:77771328-77774894 | + | chr5_fcluster2876 | 77773861-77773954 | 3 |
| ENST00000412431.2 | chr5:148872950-148874782 | - | chr5_rcluster5518 | 148873285-148873378 | 3 |
| ENST00000413221.2 | chr5:111497849-111499973 | + | chr5_fcluster4019 | 111499257-111499350 | 10 |
| ENST00000502171.2 | chr5:52405673-52405978 | + | chr5_fcluster1750 | 52405691-52405784 | 3 |
| ENST00000538629.1 | chr5:77771328-77776562 | + | chr5_fcluster2876 | 77773861-77773954 | 3 |
| ENST00000545462.1 | chr5:180257673-180258618 | - | chr5_rcluster6845 | 180258282-180258375 | 3 |
| ENST00000320280.7 | chr5:77771328-77774638 | + | chr5_fcluster2875 | 77773390-77773485 | 4 |
| ENST00000339292.4 | chr5:77771328-77774894 | + | chr5_fcluster2875 | 77773390-77773485 | 4 |
| ENST00000501855.2 | chr5:180261313-180262726 | + | chr5_fcluster6962 | 180262088-180262183 | 4 |
| ENST00000501937.2 | chr5:180261313-180262678 | + | chr5_fcluster6962 | 180262088-180262183 | 4 |
| ENST00000502001.2 | chr5:8460047-8460776 | + | chr5_fcluster373 | 8460466-8460561 | 2 |
| ENST00000502162.2 | chr5:180261313-180262726 | + | chr5_fcluster6962 | 180262088-180262183 | 4 |
| ENST00000524295.1 | chr5:151646827-151650009 | + | chr5_fcluster5768 | 151647964-151648059 | 2 |
| ENST00000538629.1 | chr5:77771328-77776562 | + | chr5_fcluster2875 | 77773390-77773485 | 4 |
| ENST00000339292.4 | chr5:77656408-77656552 | + | chr5_fcluster2864 | 77656437-77656533 | 4 |
| ENST00000508271.2 | chr5:77656436-77656552 | + | chr5_fcluster2864 | 77656437-77656533 | 4 |
| ENST00000508822.1 | chr5:77656436-77656552 | + | chr5_fcluster2864 | 77656437-77656533 | 4 |
| ENST00000509998.1 | chr5:77656436-77656552 | + | chr5_fcluster2864 | 77656437-77656533 | 4 |
| ENST00000512693.1 | chr5:108658698-108662070 | - | chr5_rcluster3882 | 108661235-108661331 | 10 |
| ENST00000538629.1 | chr5:77656340-77656552 | + | chr5_fcluster2864 | 77656437-77656533 | 4 |
| ENST00000504474.1 | chr5:92877579-92879730 | - | chr5_rcluster3337 | 92877798-92877895 | 2 |
| ENST00000513626.1 | chr5:90606839-90609046 | - | chr5_rcluster3267 | 90607076-90607174 | 16 |
| ENST00000517927.1 | chr5:159895276-159895447 | + | chr5_fcluster6076 | 159895303-159895401 | 6 |
| ENST00000538380.1 | chr5:17169366-17170610 | - | chr5_rcluster721 | 17169834-17169932 | 3 |
| ENST00000510576.2 | chr5:151065944-151067471 | + | chr5_fcluster5723 | 151066062-151066162 | 2 |
| ENST00000439343.2 | chr6:7891855-7891969 | - | chr6_rcluster404 | 7891911-7891957 | 2 |
| ENST00000481848.2 | chr6:127759552-127765427 | - | chr6_rcluster5388 | 127762004-127762050 | 4 |
| ENST00000500702.1 | chr6:144325475-144329867 | + | chr6_fcluster5925 | 144328039-144328085 | 3 |
| ENST00000399247.2 | chr6:29694379-29694916 | - | chr6_rcluster1481 | 29694851-29694898 | 3 |
| ENST00000501288.1 | chr6:26569553-26569764 | + | chr6_fcluster1281 | 26569607-26569656 | 2 |
| ENST00000415195.1 | chr6:30484044-30484388 | + | chr6_fcluster1652 | 30484093-30484143 | 2 |
| ENST00000411553.1 | chr6:26522077-26526807 | + | chr6_fcluster1266 | 26523919-26523970 | 2 |
| ENST00000421310.1 | chr6:132223105-132223291 | + | chr6_fcluster5462 | 132223149-132223200 | 6 |
| ENST00000454596.1 | chr6:132223104-132223291 | + | chr6_fcluster5462 | 132223149-132223200 | 6 |
| ENST00000421315.1 | chr6:74000257-74001063 | + | chr6_fcluster3560 | 74000832-74000884 | 3 |
| ENST00000437615.1 | chr6:170188887-170191214 | - | chr6_rcluster7189 | 170190861-170190913 | 3 |
| ENST00000359760.5 | chr6:168226084-168227389 | - | chr6_rcluster7099 | 168227119-168227172 | 2 |
| ENST00000412685.1 | chr6:30294132-30294628 | - | chr6_rcluster1510 | 30294384-30294437 | 2 |
| ENST00000426635.1 | chr6:72129770-72130472 | - | chr6_rcluster3357 | 72129929-72129982 | 5 |
| ENST00000426882.1 | chr6:30294132-30294927 | - | chr6_rcluster1510 | 30294384-30294437 | 2 |
| ENST00000429530.1 | chr6:64531134-64532535 | + | chr6_fcluster3300 | 64531184-64531239 | 3 |
| ENST00000367477.3 | chr6:147171962-147172467 | - | chr6_rcluster6221 | 147172242-147172300 | 7 |
| ENST00000427394.1 | chr6:147171962-147172467 | - | chr6_rcluster6221 | 147172242-147172300 | 7 |
| ENST00000512967.1 | chr6:6695028-6697395 | - | chr6_rcluster332 | 6695222-6695281 | 10 |
| ENST00000435295.1 | chr6:155575398-155577858 | - | chr6_rcluster6578 | 155575876-155575936 | 2 |
| ENST00000439343.2 | chr6:8041373-8041501 | - | chr6_rcluster417 | 8041401-8041466 | 6 |
| ENST00000411553.1 | chr6:26522077-26526807 | + | chr6_fcluster1267 | 26524205-26524272 | 3 |
| ENST00000423730.2 | chr6:73972498-73972919 | - | chr6_rcluster3409 | 73972809-73972876 | 2 |
| ENST00000479822.1 | chr6:10434876-10435068 | - | chr6_rcluster485 | 10434963-10435030 | 8 |
| ENST00000487130.1 | chr6:10434876-10435107 | - | chr6_rcluster485 | 10434963-10435030 | 8 |
| ENST00000491317.1 | chr6:10434747-10435050 | - | chr6_rcluster485 | 10434963-10435030 | 8 |
| ENST00000496285.1 | chr6:10434747-10435055 | - | chr6_rcluster485 | 10434963-10435030 | 8 |
| ENST00000411553.1 | chr6:26522077-26526807 | + | chr6_fcluster1262 | 26522106-26522176 | 3 |
| ENST00000438051.2 | chr6:166756133-166756635 | + | chr6_fcluster6844 | 166756167-166756237 | 5 |
| ENST00000477984.1 | chr6:33560775-33561115 | - | chr6_rcluster1901 | 33561027-33561097 | 8 |
| ENST00000445974.1 | chr6:113683734-113683872 | - | chr6_rcluster4901 | 113683780-113683854 | 3 |
| ENST00000357510.6 | chr6:168226198-168227012 | - | chr6_rcluster7098 | 168226898-168226973 | 3 |
| ENST00000359760.5 | chr6:168226084-168227389 | - | chr6_rcluster7098 | 168226898-168226973 | 3 |
| ENST00000411553.1 | chr6:26522077-26526807 | + | chr6_fcluster1269 | 26525917-26525992 | 8 |
| ENST00000414943.1 | chr6:168226372-168227019 | - | chr6_rcluster7098 | 168226898-168226973 | 3 |
| ENST00000500702.1 | chr6:144325475-144329867 | + | chr6_fcluster5926 | 144329174-144329249 | 2 |
| ENST00000499196.1 | chr6:32862501-32862803 | + | chr6_fcluster1891 | 32862536-32862615 | 4 |
| ENST00000423260.1 | chr6:16762584-16762883 | + | chr6_fcluster790 | 16762645-16762726 | 2 |
| ENST00000450930.1 | chr6:16762584-16762849 | + | chr6_fcluster790 | 16762645-16762726 | 2 |
| ENST00000413745.1 | chr6:136365603-136366065 | - | chr6_rcluster5764 | 136365689-136365772 | 3 |
| ENST00000419926.1 | chr6:136364991-136366071 | - | chr6_rcluster5764 | 136365689-136365772 | 3 |
| ENST00000420981.1 | chr6:2854892-2855924 | - | chr6_rcluster130 | 2855492-2855576 | 2 |
| ENST00000545177.1 | chr6:2854893-2855924 | - | chr6_rcluster130 | 2855492-2855576 | 2 |
| ENST00000452647.1 | chr6:99968570-99969023 | + | chr6_fcluster4362 | 99968883-99968968 | 9 |
| ENST00000428903.1 | chr6:25056548-25056892 | + | chr6_fcluster1099 | 25056582-25056668 | 2 |
| ENST00000439343.2 | chr6:7881756-7883499 | - | chr6_rcluster399 | 7882313-7882403 | 5 |
| ENST00000500590.1 | chr6:43190729-43191598 | - | chr6_rcluster2442 | 43191228-43191320 | 4 |
| ENST00000422437.1 | chr6:32138193-32139334 | + | chr6_fcluster1855 | 32139147-32139240 | 5 |
| ENST00000439343.2 | chr6:7899810-7899914 | - | chr6_rcluster406 | 7899812-7899907 | 4 |
| ENST00000427501.1 | chr6:86387143-86387711 | - | chr6_rcluster3916 | 86387295-86387391 | 12929 |
| ENST00000431043.1 | chr6:86386847-86387750 | - | chr6_rcluster3916 | 86387295-86387391 | 12929 |
| ENST00000506048.1 | chr6:157099087-157099659 | + | chr6_fcluster6405 | 157099151-157099250 | 2 |
| ENST00000437621.2 | chr7:1624021-1629262 | + | chr7_fcluster125 | 1624405-1624451 | 2 |
| ENST00000437621.2 | chr7:1624021-1629262 | + | chr7_fcluster126 | 1626459-1626505 | 2 |
| ENST00000437964.1 | chr7:1624021-1626181 | + | chr7_fcluster125 | 1624405-1624451 | 2 |
| ENST00000457484.2 | chr7:1624021-1625253 | + | chr7_fcluster125 | 1624405-1624451 | 2 |
| ENST00000422542.1 | chr7:22900167-22900458 | + | chr7_fcluster952 | 22900193-22900242 | 2 |
| ENST00000452622.1 | chr7:560029-562953 | + | chr7_fcluster26 | 562228-562277 | 3 |
| ENST00000419813.1 | chr7:23140848-23143197 | - | chr7_rcluster967 | 23141988-23142039 | 2 |
| ENST00000456775.1 | chr7:116592501-116594388 | - | chr7_rcluster4682 | 116593666-116593717 | 7 |
| ENST00000439105.1 | chr7:84569065-84569561 | - | chr7_rcluster3360 | 84569413-84569465 | 2 |
| ENST00000451264.1 | chr7:26416052-26416321 | - | chr7_rcluster1110 | 26416099-26416151 | 3 |
| ENST00000340510.4 | chr7:39822335-39822398 | + | chr7_fcluster1589 | 39822345-39822398 | 7 |
| ENST00000429611.3 | chr7:27135744-27136007 | + | chr7_fcluster1121 | 27135830-27135883 | 4 |
| ENST00000434063.3 | chr7:27135714-27136007 | + | chr7_fcluster1121 | 27135830-27135883 | 4 |
| ENST00000440855.2 | chr7:5553486-5553829 | + | chr7_fcluster361 | 5553509-5553562 | 18 |
| ENST00000448955.1 | chr7:39822335-39822398 | + | chr7_fcluster1589 | 39822345-39822398 | 7 |
| ENST00000465466.1 | chr7:140395137-140396620 | - | chr7_rcluster5569 | 140396368-140396421 | 2 |
| ENST00000495032.1 | chr7:27135811-27135946 | + | chr7_fcluster1121 | 27135830-27135883 | 4 |
| ENST00000415652.1 | chr7:158383599-158383812 | + | chr7_fcluster6414 | 158383670-158383724 | 2 |
| ENST00000447430.1 | chr7:130612322-130613408 | + | chr7_fcluster5246 | 130613016-130613070 | 2 |
| ENST00000439234.1 | chr7:81656972-81659271 | + | chr7_fcluster3377 | 81659092-81659147 | 984 |
| ENST00000412266.1 | chr7:2757468-2760269 | - | chr7_rcluster223 | 2759860-2759916 | 94 |
| ENST00000322982.3 | chr7:28992975-28997934 | - | chr7_rcluster1230 | 28995941-28996000 | 2 |
| ENST00000412669.1 | chr7:90226512-90226667 | - | chr7_rcluster3534 | 90226571-90226630 | 2 |
| ENST00000444210.2 | chr7:5517420-5519442 | + | chr7_fcluster360 | 5517931-5517991 | 2 |
| ENST00000466677.1 | chr7:151106358-151106468 | + | chr7_fcluster6108 | 151106404-151106466 | 2 |
| ENST00000480632.1 | chr7:151106323-151106468 | + | chr7_fcluster6108 | 151106404-151106466 | 2 |
| ENST00000489632.1 | chr7:151106248-151106468 | + | chr7_fcluster6108 | 151106404-151106466 | 2 |
| ENST00000359941.5 | chr7:86974360-86974831 | - | chr7_rcluster3439 | 86974567-86974633 | 2 |
| ENST00000432193.1 | chr7:86974551-86974802 | - | chr7_rcluster3439 | 86974567-86974633 | 2 |
| ENST00000492523.1 | chr7:100034007-100034133 | + | chr7_fcluster4088 | 100034044-100034110 | 3 |
| ENST00000439234.1 | chr7:81656972-81659271 | + | chr7_fcluster3376 | 81657381-81657448 | 2 |
| ENST00000445459.1 | chr7:131948834-131949060 | + | chr7_fcluster5322 | 131948954-131949021 | 3 |
| ENST00000454066.1 | chr7:81656972-81657659 | + | chr7_fcluster3376 | 81657381-81657448 | 2 |
| ENST00000451786.1 | chr7:130794497-130794935 | - | chr7_rcluster5146 | 130794575-130794644 | 3 |
| ENST00000452622.1 | chr7:560029-562953 | + | chr7_fcluster25 | 560732-560802 | 2 |
| ENST00000492679.1 | chr7:128502506-128503066 | - | chr7_rcluster5023 | 128502990-128503062 | 2 |
| ENST00000471299.1 | chr7:149779-150132 | + | chr7_fcluster6 | 150013-150088 | 3 |
| ENST00000479592.1 | chr7:149732-150132 | + | chr7_fcluster6 | 150013-150088 | 3 |
| ENST00000484550.1 | chr7:149598-152547 | + | chr7_fcluster6 | 150013-150088 | 3 |
| ENST00000429254.1 | chr7:100942895-100943147 | + | chr7_fcluster4181 | 100942925-100943001 | 12 |
| ENST00000480284.1 | chr7:156803500-156804001 | + | chr7_fcluster6325 | 156803573-156803650 | 3 |
| ENST00000414797.1 | chr7:79088164-79088315 | + | chr7_fcluster3329 | 79088234-79088314 | 10 |
| ENST00000417881.1 | chr7:94784518-94786263 | - | chr7_rcluster3699 | 94785232-94785312 | 2 |
| ENST00000422093.1 | chr7:79088164-79088315 | + | chr7_fcluster3329 | 79088234-79088314 | 10 |
| ENST00000424477.1 | chr7:79088164-79088315 | + | chr7_fcluster3329 | 79088234-79088314 | 10 |
| ENST00000426835.1 | chr7:79088164-79088315 | + | chr7_fcluster3329 | 79088234-79088314 | 10 |
| ENST00000429408.1 | chr7:79088164-79088315 | + | chr7_fcluster3329 | 79088234-79088314 | 10 |
| ENST00000432668.1 | chr7:22895706-22896175 | + | chr7_fcluster950 | 22895910-22895990 | 6 |
| ENST00000446159.1 | chr7:79088164-79088315 | + | chr7_fcluster3329 | 79088234-79088314 | 10 |
| ENST00000447643.1 | chr7:44043646-44044934 | - | chr7_rcluster1744 | 44044567-44044647 | 3 |
| ENST00000448195.1 | chr7:79088164-79088315 | + | chr7_fcluster3329 | 79088234-79088314 | 10 |
| ENST00000448636.1 | chr7:79088164-79088315 | + | chr7_fcluster3329 | 79088234-79088314 | 10 |
| ENST00000451809.1 | chr7:79088164-79088315 | + | chr7_fcluster3329 | 79088234-79088314 | 10 |
| ENST00000452320.1 | chr7:79088022-79088315 | + | chr7_fcluster3329 | 79088234-79088314 | 10 |
| ENST00000456775.1 | chr7:116592501-116594388 | - | chr7_rcluster4683 | 116593972-116594053 | 2 |
| ENST00000479766.1 | chr7:27227439-27227639 | + | chr7_fcluster1141 | 27227459-27227540 | 2 |
| ENST00000413812.1 | chr7:126868428-126869975 | + | chr7_fcluster5022 | 126869558-126869640 | 8 |
| ENST00000489695.1 | chr7:27209087-27209356 | - | chr7_rcluster1143 | 27209109-27209191 | 15955 |
| ENST00000452714.1 | chr7:111459435-111461829 | + | chr7_fcluster4605 | 111461038-111461122 | 2 |
| ENST00000413042.1 | chr7:23245956-23247664 | + | chr7_fcluster968 | 23247072-23247157 | 9 |
| ENST00000429254.1 | chr7:100944051-100944551 | + | chr7_fcluster4182 | 100944457-100944542 | 2 |
| ENST00000517635.2 | chr7:27154968-27155962 | + | chr7_fcluster1123 | 27155137-27155222 | 4 |
| ENST00000437450.1 | chr7:107384184-107384360 | + | chr7_fcluster4493 | 107384245-107384334 | 2 |
| ENST00000437621.2 | chr7:1609710-1609915 | + | chr7_fcluster124 | 1609731-1609820 | 3 |
| ENST00000457484.2 | chr7:1609726-1610169 | + | chr7_fcluster124 | 1609731-1609820 | 3 |
| ENST00000413042.1 | chr7:23245956-23247664 | + | chr7_fcluster967 | 23246175-23246265 | 2 |
| ENST00000433446.1 | chr7:86781067-86781598 | - | chr7_rcluster3428 | 86781232-86781322 | 2 |
| ENST00000456775.1 | chr7:116592501-116594388 | - | chr7_rcluster4684 | 116594287-116594377 | 9 |
| ENST00000513631.1 | chr7:96635696-96637022 | - | chr7_rcluster3777 | 96636124-96636221 | 2 |
| ENST00000433079.1 | chr7:130628922-130629648 | - | chr7_rcluster5137 | 130629084-130629182 | 2 |
| ENST00000443623.1 | chr7:130628928-130629206 | - | chr7_rcluster5137 | 130629084-130629182 | 2 |
| ENST00000451786.1 | chr7:130628927-130630594 | - | chr7_rcluster5137 | 130629084-130629182 | 2 |
| ENST00000366161.2 | chr7:153754519-153756231 | - | chr7_rcluster6042 | 153754778-153754877 | 39 |
| ENST00000425591.1 | chr7:153754519-153756646 | - | chr7_rcluster6042 | 153754778-153754877 | 39 |
| ENST00000512113.1 | chr8:143751351-143751550 | - | chr8_rcluster8364 | 143751357-143751402 | 3 |
| ENST00000517482.1 | chr8:125283925-125284051 | + | chr8_fcluster7239 | 125283955-125284002 | 4 |
| ENST00000521915.1 | chr8:38023531-38023636 | - | chr8_rcluster2228 | 38023549-38023596 | 12 |
| ENST00000500118.2 | chr8:6263228-6264069 | - | chr8_rcluster297 | 6263998-6264047 | 7 |
| ENST00000560295.1 | chr8:141530256-141539600 | - | chr8_rcluster8167 | 141532453-141532502 | 12 |
| ENST00000276681.6 | chr8:120255658-120257909 | + | chr8_fcluster6950 | 120255686-120255736 | 2 |
| ENST00000520820.1 | chr8:103251623-103251928 | + | chr8_fcluster6095 | 103251734-103251784 | 4 |
| ENST00000521748.1 | chr8:120255658-120257913 | + | chr8_fcluster6950 | 120255686-120255736 | 2 |
| ENST00000522112.2 | chr8:120255658-120255768 | + | chr8_fcluster6950 | 120255686-120255736 | 2 |
| ENST00000531508.1 | chr8:120255658-120255888 | + | chr8_fcluster6950 | 120255686-120255736 | 2 |
| ENST00000534619.1 | chr8:120255658-120255762 | + | chr8_fcluster6950 | 120255686-120255736 | 2 |
| ENST00000339066.7 | chr8:64122242-64122725 | + | chr8_fcluster3805 | 64122265-64122317 | 2 |
| ENST00000500410.1 | chr8:12455145-12455546 | - | chr8_rcluster752 | 12455236-12455288 | 33 |
| ENST00000517303.1 | chr8:64122242-64122663 | + | chr8_fcluster3805 | 64122265-64122317 | 2 |
| ENST00000517371.1 | chr8:64122242-64125003 | + | chr8_fcluster3805 | 64122265-64122317 | 2 |
| ENST00000519726.1 | chr8:8084721-8085385 | - | chr8_rcluster451 | 8085078-8085130 | 3 |
| ENST00000520640.1 | chr8:64122242-64122725 | + | chr8_fcluster3805 | 64122265-64122317 | 2 |
| ENST00000521674.1 | chr8:64122242-64125344 | + | chr8_fcluster3805 | 64122265-64122317 | 2 |
| ENST00000524135.1 | chr8:64122242-64125344 | + | chr8_fcluster3805 | 64122265-64122317 | 2 |
| ENST00000539294.1 | chr8:64122242-64125346 | + | chr8_fcluster3805 | 64122265-64122317 | 2 |
| ENST00000542911.1 | chr8:64122242-64123304 | + | chr8_fcluster3805 | 64122265-64122317 | 2 |
| ENST00000276681.6 | chr8:120255658-120257909 | + | chr8_fcluster6953 | 120256428-120256482 | 3 |
| ENST00000276681.6 | chr8:120255658-120257909 | + | chr8_fcluster6955 | 120257031-120257085 | 2 |
| ENST00000518932.1 | chr8:106799678-106799824 | - | chr8_rcluster6197 | 106799756-106799810 | 2 |
| ENST00000520594.1 | chr8:106799678-106799824 | - | chr8_rcluster6197 | 106799756-106799810 | 2 |
| ENST00000521696.1 | chr8:100025098-100025272 | - | chr8_rcluster5703 | 100025180-100025234 | 5 |
| ENST00000521748.1 | chr8:120255658-120257913 | + | chr8_fcluster6953 | 120256428-120256482 | 3 |
| ENST00000521748.1 | chr8:120255658-120257913 | + | chr8_fcluster6955 | 120257031-120257085 | 2 |
| ENST00000524045.1 | chr8:106799678-106799824 | - | chr8_rcluster6197 | 106799756-106799810 | 2 |
| ENST00000500162.1 | chr8:12516640-12517338 | - | chr8_rcluster763 | 12516692-12516747 | 2 |
| ENST00000500162.1 | chr8:12522788-12523097 | - | chr8_rcluster766 | 12522840-12522895 | 2 |
| ENST00000500410.1 | chr8:12513485-12514228 | - | chr8_rcluster761 | 12514083-12514138 | 3 |
| ENST00000500410.1 | chr8:12516640-12516880 | - | chr8_rcluster763 | 12516692-12516747 | 2 |
| ENST00000500955.1 | chr8:12522788-12523137 | - | chr8_rcluster766 | 12522840-12522895 | 2 |
| ENST00000517384.1 | chr8:22423511-22423957 | - | chr8_rcluster1331 | 22423604-22423659 | 15 |
| ENST00000501224.2 | chr8:28553719-28555991 | - | chr8_rcluster1691 | 28554975-28555031 | 2 |
| ENST00000517909.1 | chr8:65486869-65489185 | - | chr8_rcluster3724 | 65488725-65488781 | 2 |
| ENST00000520834.1 | chr8:65486864-65489185 | - | chr8_rcluster3724 | 65488725-65488781 | 2 |
| ENST00000500843.2 | chr8:32901886-32902373 | + | chr8_fcluster2143 | 32901959-32902016 | 2 |
| ENST00000517714.1 | chr8:37483258-37483391 | - | chr8_rcluster2172 | 37483275-37483332 | 3 |
| ENST00000520819.1 | chr8:32901886-32902320 | + | chr8_fcluster2143 | 32901959-32902016 | 2 |
| ENST00000399870.3 | chr8:12310555-12312147 | + | chr8_fcluster796 | 12311740-12311798 | 2 |
| ENST00000501016.2 | chr8:99055964-99056349 | + | chr8_fcluster5806 | 99056104-99056162 | 2 |
| ENST00000517562.1 | chr8:91968168-91971258 | - | chr8_rcluster5185 | 91969493-91969551 | 2 |
| ENST00000507178.1 | chr8:143738875-143747746 | - | chr8_rcluster8360 | 143740483-143740543 | 2 |
| ENST00000514531.1 | chr8:143739807-143745257 | - | chr8_rcluster8360 | 143740483-143740543 | 2 |
| ENST00000518143.1 | chr8:77585063-77585268 | - | chr8_rcluster4435 | 77585182-77585242 | 2 |
| ENST00000524003.1 | chr8:92080140-92082050 | - | chr8_rcluster5197 | 92081543-92081604 | 2 |
| ENST00000500989.2 | chr8:126953374-126958426 | - | chr8_rcluster7291 | 126957715-126957777 | 3 |
| ENST00000501897.1 | chr8:22938762-22941132 | + | chr8_fcluster1429 | 22939849-22939912 | 4 |
| ENST00000339066.7 | chr8:64098707-64100303 | + | chr8_fcluster3803 | 64099802-64099867 | 6 |
| ENST00000434924.2 | chr8:64098733-64100303 | + | chr8_fcluster3803 | 64099802-64099867 | 6 |
| ENST00000517303.1 | chr8:64098707-64100303 | + | chr8_fcluster3803 | 64099802-64099867 | 6 |
| ENST00000520640.1 | chr8:64098707-64100303 | + | chr8_fcluster3803 | 64099802-64099867 | 6 |
| ENST00000521674.1 | chr8:64098707-64100303 | + | chr8_fcluster3803 | 64099802-64099867 | 6 |
| ENST00000524135.1 | chr8:64098707-64100303 | + | chr8_fcluster3803 | 64099802-64099867 | 6 |
| ENST00000539294.1 | chr8:64098733-64100303 | + | chr8_fcluster3803 | 64099802-64099867 | 6 |
| ENST00000542911.1 | chr8:64098733-64100141 | + | chr8_fcluster3803 | 64099802-64099867 | 6 |
| ENST00000502083.2 | chr8:22863895-22865630 | - | chr8_rcluster1371 | 22865149-22865215 | 3 |
| ENST00000521674.1 | chr8:64122242-64125344 | + | chr8_fcluster3810 | 64125249-64125315 | 3 |
| ENST00000524135.1 | chr8:64122242-64125344 | + | chr8_fcluster3810 | 64125249-64125315 | 3 |
| ENST00000539294.1 | chr8:64122242-64125346 | + | chr8_fcluster3810 | 64125249-64125315 | 3 |
| ENST00000276681.6 | chr8:120255658-120257909 | + | chr8_fcluster6952 | 120256276-120256343 | 2 |
| ENST00000521748.1 | chr8:120255658-120257913 | + | chr8_fcluster6952 | 120256276-120256343 | 2 |
| ENST00000517675.1 | chr8:9757575-9760907 | - | chr8_rcluster563 | 9757782-9757850 | 2 |
| ENST00000500112.1 | chr8:128220112-128221962 | - | chr8_rcluster7375 | 128221099-128221169 | 4 |
| ENST00000514980.1 | chr8:22402700-22402918 | - | chr8_rcluster1327 | 22402725-22402796 | 2 |
| ENST00000507178.1 | chr8:143738875-143747746 | - | chr8_rcluster8362 | 143744151-143744223 | 2 |
| ENST00000514531.1 | chr8:143739807-143745257 | - | chr8_rcluster8362 | 143744151-143744223 | 2 |
| ENST00000519753.1 | chr8:30240645-30240878 | - | chr8_rcluster1794 | 30240799-30240872 | 2 |
| ENST00000523643.1 | chr8:30240645-30240878 | - | chr8_rcluster1794 | 30240799-30240872 | 2 |
| ENST00000521090.1 | chr8:137821986-137822280 | + | chr8_fcluster7988 | 137822178-137822252 | 3 |
| ENST00000560295.1 | chr8:141530256-141539600 | - | chr8_rcluster8174 | 141536259-141536333 | 2 |
| ENST00000522875.1 | chr8:129108765-129108902 | + | chr8_fcluster7537 | 129108826-129108901 | 2 |
| ENST00000523103.1 | chr8:20147321-20147969 | + | chr8_fcluster1224 | 20147684-20147759 | 11013 |
| ENST00000506428.1 | chr8:61315772-61317808 | - | chr8_rcluster3486 | 61317236-61317312 | 2 |
| ENST00000521428.1 | chr8:127512123-127512548 | + | chr8_fcluster7394 | 127512127-127512203 | 3 |
| ENST00000520944.1 | chr8:67837673-67837831 | - | chr8_rcluster3906 | 67837701-67837778 | 21 |
| ENST00000500118.2 | chr8:6263228-6264069 | - | chr8_rcluster296 | 6263834-6263912 | 2 |
| ENST00000517371.1 | chr8:64122242-64125003 | + | chr8_fcluster3809 | 64124338-64124417 | 6 |
| ENST00000521674.1 | chr8:64122242-64125344 | + | chr8_fcluster3809 | 64124338-64124417 | 6 |
| ENST00000524135.1 | chr8:64122242-64125344 | + | chr8_fcluster3809 | 64124338-64124417 | 6 |
| ENST00000539294.1 | chr8:64122242-64125346 | + | chr8_fcluster3809 | 64124338-64124417 | 6 |
| ENST00000524003.1 | chr8:92080140-92082050 | - | chr8_rcluster5196 | 92081090-92081175 | 2 |
| ENST00000526470.1 | chr8:61544115-61544466 | - | chr8_rcluster3506 | 61544379-61544465 | 4 |
| ENST00000532768.1 | chr8:61544069-61544466 | - | chr8_rcluster3506 | 61544379-61544465 | 4 |
| ENST00000560295.1 | chr8:141530256-141539600 | - | chr8_rcluster8169 | 141534270-141534357 | 3 |
| ENST00000519655.1 | chr8:90624014-90624218 | - | chr8_rcluster5088 | 90624014-90624102 | 4 |
| ENST00000524166.1 | chr8:90623586-90624218 | - | chr8_rcluster5088 | 90624014-90624102 | 4 |
| ENST00000523881.1 | chr8:73793731-73793830 | - | chr8_rcluster4251 | 73793735-73793824 | 3 |
| ENST00000517411.1 | chr8:144363653-144363830 | - | chr8_rcluster8416 | 144363703-144363793 | 3 |
| ENST00000518073.1 | chr8:144363653-144363860 | - | chr8_rcluster8416 | 144363703-144363793 | 3 |
| ENST00000524335.1 | chr8:144363653-144363835 | - | chr8_rcluster8416 | 144363703-144363793 | 3 |
| ENST00000522373.1 | chr8:140472306-140473210 | - | chr8_rcluster8096 | 140472510-140472601 | 6 |
| ENST00000560295.1 | chr8:141530256-141539600 | - | chr8_rcluster8172 | 141535203-141535294 | 5 |
| ENST00000506428.1 | chr8:61315772-61317808 | - | chr8_rcluster3485 | 61315778-61315870 | 2 |
| ENST00000221169.5 | chr8:24812987-24814624 | - | chr8_rcluster1490 | 24813604-24813699 | 2 |
| ENST00000519840.1 | chr8:72587536-72587770 | + | chr8_fcluster4278 | 72587625-72587720 | 7 |
| ENST00000521131.1 | chr8:72587613-72587770 | + | chr8_fcluster4278 | 72587625-72587720 | 7 |
| ENST00000560295.1 | chr8:141530256-141539600 | - | chr8_rcluster8170 | 141534673-141534769 | 3 |
| ENST00000446592.3 | chr8:130363938-130365226 | - | chr8_rcluster7519 | 130364861-130364958 | 2 |
| ENST00000520048.1 | chr8:130364676-130365226 | - | chr8_rcluster7519 | 130364861-130364958 | 2 |
| ENST00000523151.1 | chr8:130363938-130365226 | - | chr8_rcluster7519 | 130364861-130364958 | 2 |
| ENST00000507178.1 | chr8:143738875-143747746 | - | chr8_rcluster8363 | 143745585-143745683 | 2 |
| ENST00000521103.1 | chr8:143328949-143329227 | - | chr8_rcluster8332 | 143329002-143329100 | 91 |
| ENST00000500118.2 | chr8:6261073-6262641 | - | chr8_rcluster295 | 6262141-6262240 | 3 |
| ENST00000517371.1 | chr8:64081214-64081460 | + | chr8_fcluster3801 | 64081321-64081420 | 6 |
| ENST00000518438.1 | chr8:64081247-64081460 | + | chr8_fcluster3801 | 64081321-64081420 | 6 |
| ENST00000518763.1 | chr8:64081221-64081460 | + | chr8_fcluster3801 | 64081321-64081420 | 6 |
| ENST00000519428.1 | chr8:64081181-64081460 | + | chr8_fcluster3801 | 64081321-64081420 | 6 |
| ENST00000521674.1 | chr8:64081113-64081460 | + | chr8_fcluster3801 | 64081321-64081420 | 6 |
| ENST00000522282.1 | chr8:64081122-64081460 | + | chr8_fcluster3801 | 64081321-64081420 | 6 |
| ENST00000523455.1 | chr8:64081212-64081460 | + | chr8_fcluster3801 | 64081321-64081420 | 6 |
| ENST00000524135.1 | chr8:64081215-64081460 | + | chr8_fcluster3801 | 64081321-64081420 | 6 |
| ENST00000539294.1 | chr8:64081122-64081460 | + | chr8_fcluster3801 | 64081321-64081420 | 6 |
| ENST00000542911.1 | chr8:64081203-64081460 | + | chr8_fcluster3801 | 64081321-64081420 | 6 |
| ENST00000423918.1 | chr9:132275699-132275947 | + | chr9_fcluster5050 | 132275806-132275851 | 3 |
| ENST00000427548.1 | chr9:115759496-115761418 | - | chr9_rcluster4250 | 115760306-115760351 | 4 |
| ENST00000429493.1 | chr9:37086666-37090398 | + | chr9_fcluster1426 | 37088499-37088544 | 6 |
| ENST00000425633.1 | chr9:27278360-27278726 | - | chr9_rcluster994 | 27278613-27278660 | 2 |
| ENST00000436889.2 | chr9:70645994-70648248 | - | chr9_rcluster2249 | 70646720-70646767 | 4 |
| ENST00000422679.1 | chr9:27658-30445 | + | chr9_fcluster0 | 29054-29102 | 2 |
| ENST00000427548.1 | chr9:115759496-115761418 | - | chr9_rcluster4249 | 115760138-115760186 | 3 |
| ENST00000451160.1 | chr9:109685635-109685884 | + | chr9_fcluster3852 | 109685797-109685845 | 3 |
| ENST00000427246.1 | chr9:131448621-131449189 | + | chr9_fcluster4948 | 131448880-131448933 | 7 |
| ENST00000366109.2 | chr9:100000706-100000960 | - | chr9_rcluster3538 | 100000903-100000958 | 3 |
| ENST00000540557.1 | chr9:38067927-38068684 | - | chr9_rcluster1600 | 38067985-38068040 | 2 |
| ENST00000366322.2 | chr9:68408724-68410274 | + | chr9_fcluster2056 | 68408733-68408789 | 6 |
| ENST00000429493.1 | chr9:37086666-37090398 | + | chr9_fcluster1427 | 37088916-37088972 | 2 |
| ENST00000344538.4 | chr9:43091404-43091857 | - | chr9_rcluster1814 | 43091690-43091747 | 2 |
| ENST00000423380.1 | chr9:93867240-93867664 | - | chr9_rcluster3174 | 93867428-93867486 | 2 |
| ENST00000429493.1 | chr9:37086666-37090398 | + | chr9_fcluster1428 | 37089099-37089158 | 2 |
| ENST00000457558.1 | chr9:85043115-85043202 | + | chr9_fcluster2711 | 85043119-85043178 | 2 |
| ENST00000429562.2 | chr9:103115361-103115863 | + | chr9_fcluster3583 | 103115369-103115430 | 9 |
| ENST00000449175.1 | chr9:125871774-125877756 | - | chr9_rcluster4696 | 125873836-125873897 | 9 |
| ENST00000445604.1 | chr9:66553244-66553911 | - | chr9_rcluster2032 | 66553259-66553322 | 2 |
| ENST00000454645.1 | chr9:42018929-42019580 | - | chr9_rcluster1753 | 42018944-42019007 | 2 |
| ENST00000425533.1 | chr9:32552347-32552477 | + | chr9_fcluster1104 | 32552348-32552416 | 12 |
| ENST00000450093.1 | chr9:32552233-32552477 | + | chr9_fcluster1104 | 32552348-32552416 | 12 |
| ENST00000453396.1 | chr9:32552233-32552477 | + | chr9_fcluster1104 | 32552348-32552416 | 12 |
| ENST00000456198.1 | chr9:26956126-26956293 | - | chr9_rcluster982 | 26956165-26956233 | 4 |
| ENST00000458036.1 | chr9:32552328-32552477 | + | chr9_fcluster1104 | 32552348-32552416 | 12 |
| ENST00000540066.1 | chr9:32552345-32553005 | + | chr9_fcluster1104 | 32552348-32552416 | 12 |
| ENST00000427548.1 | chr9:115759496-115761418 | - | chr9_rcluster4248 | 115759832-115759903 | 3 |
| ENST00000315293.2 | chr9:67017401-67019064 | - | chr9_rcluster2048 | 67018663-67018738 | 7 |
| ENST00000426350.1 | chr9:67017401-67019064 | - | chr9_rcluster2048 | 67018663-67018738 | 7 |
| ENST00000354752.4 | chr9:99924038-99924248 | - | chr9_rcluster3519 | 99924108-99924184 | 3 |
| ENST00000430633.1 | chr9:136891331-136893224 | + | chr9_fcluster5390 | 136891607-136891684 | 3 |
| ENST00000432807.1 | chr9:136890968-136893224 | + | chr9_fcluster5390 | 136891607-136891684 | 3 |
| ENST00000413932.1 | chr9:71736401-71737148 | + | chr9_fcluster2230 | 71736430-71736509 | 2 |
| ENST00000434871.1 | chr9:37505575-37506561 | + | chr9_fcluster1475 | 37505811-37505893 | 3 |
| ENST00000449175.1 | chr9:125871774-125877756 | - | chr9_rcluster4695 | 125872455-125872537 | 24 |
| ENST00000545631.1 | chr9:125871781-125873444 | - | chr9_rcluster4695 | 125872455-125872537 | 24 |
| ENST00000540066.1 | chr9:32552345-32553005 | + | chr9_fcluster1105 | 32552713-32552797 | 5 |
| ENST00000442982.1 | chr9:123613517-123614881 | + | chr9_fcluster4407 | 123614117-123614202 | 2 |
| ENST00000447891.1 | chr9:123613517-123616651 | + | chr9_fcluster4407 | 123614117-123614202 | 2 |
| ENST00000450803.1 | chr9:123613517-123614245 | + | chr9_fcluster4407 | 123614117-123614202 | 2 |
| ENST00000366322.2 | chr9:68408724-68410274 | + | chr9_fcluster2058 | 68409188-68409274 | 2 |
| ENST00000425189.1 | chr9:136519709-136521659 | - | chr9_rcluster5466 | 136519767-136519855 | 3 |
| ENST00000500181.1 | chr9:1046080-1047957 | + | chr9_fcluster65 | 1046219-1046308 | 3 |
| ENST00000500555.1 | chr9:74381170-74383326 | + | chr9_fcluster2349 | 74383029-74383121 | 4 |
| ENST00000426157.1 | chr9:127021530-127021773 | - | chr9_rcluster4762 | 127021605-127021699 | 5 |
| ENST00000429493.1 | chr9:37086666-37090398 | + | chr9_fcluster1425 | 37087491-37087589 | 3 |
| ENST00000436786.1 | chr9:22823252-22824212 | + | chr9_fcluster846 | 22823856-22823956 | 2 |
| ENST00000424306.1 | chrX:136003404-136004935 | + | chrX_fcluster19237 | 136003890-136003935 | 2 |
| ENST00000414209.1 | chrX:73166835-73167209 | + | chrX_fcluster10500 | 73166882-73166929 | 3 |
| ENST00000415215.1 | chrX:73166835-73166931 | + | chrX_fcluster10500 | 73166882-73166929 | 3 |
| ENST00000455438.1 | chrX:36450416-36453520 | - | chrX_rcluster4083 | 36453006-36453054 | 6 |
| ENST00000538676.1 | chrX:65238667-65240264 | + | chrX_fcluster8811 | 65239504-65239552 | 6 |
| ENST00000540516.1 | chrX:65238667-65240264 | + | chrX_fcluster8811 | 65239504-65239552 | 6 |
| ENST00000553843.1 | chrX:119272906-119273026 | + | chrX_fcluster17126 | 119272926-119272974 | 1 |
| ENST00000452254.2 | chrX:119378620-119379088 | - | chrX_rcluster16701 | 119378986-119379035 | 5 |
| ENST00000536487.1 | chrX:119378620-119379122 | - | chrX_rcluster16701 | 119378986-119379035 | 5 |
| ENST00000554109.1 | chrX:119378620-119379122 | - | chrX_rcluster16701 | 119378986-119379035 | 5 |
| ENST00000399966.4 | chrX:155246328-155246502 | - | chrX_rcluster20991 | 155246394-155246446 | 3 |
| ENST00000417443.1 | chrX:134557559-134559682 | + | chrX_fcluster19026 | 134557697-134557749 | 7 |
| ENST00000429829.1 | chrX:73061218-73072588 | - | chrX_rcluster10024 | 73071436-73071488 | 2 |
| ENST00000433425.1 | chrX:134230878-134231151 | - | chrX_rcluster18631 | 134230960-134231012 | 7 |
| ENST00000433061.1 | chrX:62780713-62780929 | - | chrX_rcluster7826 | 62780739-62780792 | 3 |
| ENST00000399711.1 | chrX:68428096-68429767 | + | chrX_fcluster9348 | 68429530-68429584 | 3 |
| ENST00000446884.1 | chrX:46186987-46187080 | - | chrX_rcluster5134 | 46187014-46187068 | 3 |
| ENST00000429829.1 | chrX:73040492-73047819 | - | chrX_rcluster10013 | 73044219-73044278 | 2 |
| ENST00000429829.1 | chrX:73061218-73072588 | - | chrX_rcluster10020 | 73068927-73068986 | 2 |
| ENST00000430756.1 | chrX:115033605-115035482 | - | chrX_rcluster15906 | 115033941-115034002 | 3 |
| ENST00000449111.1 | chrX:149106847-149107004 | + | chrX_fcluster20514 | 149106861-149106924 | 4 |
| ENST00000429829.1 | chrX:73040492-73047819 | - | chrX_rcluster10011 | 73040992-73041057 | 2 |
| ENST00000366185.2 | chrX:53171031-53171889 | + | chrX_fcluster6155 | 53171677-53171743 | 3 |
| ENST00000465509.2 | chrX:83757351-83757461 | - | chrX_rcluster11832 | 83757392-83757458 | 3 |
| ENST00000399711.1 | chrX:68428096-68429767 | + | chrX_fcluster9347 | 68428101-68428169 | 4 |
| ENST00000427391.1 | chrX:130929838-130930359 | - | chrX_rcluster18119 | 130930240-130930308 | 3 |
| ENST00000374922.4 | chrX:56758497-56758632 | + | chrX_fcluster7297 | 56758501-56758571 | 4 |
| ENST00000442332.1 | chrX:122867414-122868144 | + | chrX_fcluster17531 | 122867831-122867901 | 2 |
| ENST00000451583.1 | chrX:56758497-56758632 | + | chrX_fcluster7297 | 56758501-56758571 | 4 |
| ENST00000427551.1 | chrX:24167673-24167771 | - | chrX_rcluster3101 | 24167686-24167757 | 4 |
| ENST00000361201.4 | chrX:151897083-151897278 | - | chrX_rcluster20419 | 151897122-151897194 | 3 |
| ENST00000424306.1 | chrX:136003404-136004935 | + | chrX_fcluster19236 | 136003699-136003774 | 2 |
| ENST00000370438.2 | chrX:148607262-148607803 | - | chrX_rcluster20086 | 148607537-148607614 | 6 |
| ENST00000424306.1 | chrX:136003404-136004935 | + | chrX_fcluster19239 | 136004536-136004620 | 3 |
| ENST00000435597.1 | chrX:135928859-135929956 | - | chrX_rcluster18854 | 135928983-135929068 | 2 |
| ENST00000429124.1 | chrX:73498210-73500329 | - | chrX_rcluster10138 | 73500158-73500245 | 3 |
| ENST00000429829.1 | chrX:73061218-73072588 | - | chrX_rcluster10021 | 73069935-73070023 | 2 |
| ENST00000433064.1 | chrX:146993161-146993748 | - | chrX_rcluster19967 | 146993217-146993307 | 4 |
| ENST00000545310.1 | chrX:146993161-146993335 | - | chrX_rcluster19967 | 146993217-146993307 | 4 |
| ENST00000419165.1 | chrX:103173480-103173875 | + | chrX_fcluster14754 | 103173765-103173856 | 3 |
| ENST00000429829.1 | chrX:73061218-73072588 | - | chrX_rcluster10022 | 73070984-73071077 | 4 |
| ENST00000431331.1 | chrX:75107995-75108278 | + | chrX_fcluster10903 | 75108100-75108193 | 2 |
| ENST00000508574.1 | chrX:70922523-70926305 | + | chrX_fcluster10042 | 70924999-70925092 | 5 |
| ENST00000515698.1 | chrX:70994971-70998753 | - | chrX_rcluster9585 | 70996182-70996275 | 5 |
| ENST00000416854.1 | chrX:153025946-153026067 | - | chrX_rcluster20572 | 153025968-153026065 | 3 |
| ENST00000429829.1 | chrX:73040492-73047819 | - | chrX_rcluster10012 | 73041445-73041542 | 22 |
| ENST00000412652.1 | chrX:34433299-34433654 | - | chrX_rcluster3944 | 34433555-34433653 | 16 |
| ENST00000428222.1 | chrX:118467014-118469573 | + | chrX_fcluster16890 | 118467689-118467787 | 3 |
| ENST00000429829.1 | chrX:73061218-73072588 | - | chrX_rcluster10023 | 73071201-73071300 | 2 |
| ENSTR0000399966.4 | chrY:59349334-59349508 | - | chrY_rcluster2495 | 59349400-59349452 | 3 |
| ENST00000440408.1 | chrY:14799394-14804162 | + | chrY_fcluster1489 | 14799522-14799583 | 2 |
| ENST00000324446.3 | chrY:21038955-21040114 | - | chrY_rcluster1777 | 21039459-21039558 | 2 |
