# Supplementary material for: Integrative transcriptome analysis suggest processing of a subset of long non-coding RNAs to small RNAs
Source: Biol Direct. 2012 Aug 7;7:25. doi: 10.1186/1745-6150-7-25 (PMC3477000; doi:10.1186/1745-6150-7-25)
Supplement: Additional file 4 — Table summarizing small RNA clusters discovered in our analysis, catalogued as miRNA, pasRNA and nasRNA; A) small RNA clusters mapped to lncRNA exons from lncRNAdb catalogued as different types of small RNAs B) small RNA clusters mapped to lncRNA exons from Gencode database catalogued as different types of small RNAs. [file 1745-6150-7-25-S4.doc]

**A) smallRNA clusters mapped to lncRNA exons from lncRNAdb catalogued as different types of smallRNAs.**

| **Type of smallRNAs** | **Mapped to deepbase cluster falling in lncRNA exons** | **No .of unique clusters** | **No of unique smallRNA type** |
| --- | --- | --- | --- |
| miRNA (deepBase) | 0 | 0 | 0 |
| miRNA (mirbase) | 1 | 1 | 1 |
| nasRNA (deepBase) | 0 | 0 | 0 |
| pasRNA (deepBase) | 51 | 9 | 41 |
| snoRNA (deepBase) | 0 | 0 | 0 |

**B) smallRNA clusters mapped to lncRNA exons from Gencode database catalogued as different types of smallRNAs.**

| **Type of smallRNAs** | **Mapped to deepbase cluster falling in lncRNA exons** | **No. of unique clusters** | **No of unique smallRNA type** |
| --- | --- | --- | --- |
| miRNA (deepBase) | 21 | 11 | 11 |
| miRNA (mirbase) | 1 | 1 | 1 |
| nasRNA (deepBase) | 1145 | 9 | 695 |
| pasRNA (deepBase) | 2450 | 150 | 1052 |
| snoRNA (deepBase) | 0 | 0 | 0 |
